# CDC7 inhibition induces replication stress-mediated aneuploid cells with an inflammatory phenotype sensitizing tumors to immune checkpoint blockade

**DOI:** 10.1038/s41467-023-43274-3

**Published:** 2023-11-18

**Authors:** Tomoko Yamamori Morita, Jie Yu, Yukie Kashima, Ryo Kamata, Gaku Yamamoto, Tatsunori Minamide, Chiaki Mashima, Miyuki Yoshiya, Yuta Sakae, Toyohiro Yamauchi, Yumi Hakozaki, Shun-ichiro Kageyama, Akito Nakamura, Eric Lightcap, Kosuke Tanaka, Huifeng Niu, Karuppiah Kannan, Akihiro Ohashi

**Affiliations:** 1 Division of Translational Genomics, Exploratory Oncology Research & Clinical Trial Center, National Cancer Center, Chiba, Japan; 2 Oncology Drug Discovery Unit, Takeda Development Center Americas (TDCA), Inc., Lexington, MA USA; 3 Department of Computational Biology and Medical Sciences, Graduate School of Frontier Sciences, The University of Tokyo, Chiba, Japan; 4 Department of Gastroenterology and Endoscopy, National Cancer Center Hospital East, Chiba, Japan; 5 Department of Integrated Bioscience, Graduate School of Frontier Sciences, The University of Tokyo, Tokyo, Japan; 6 Department of Radiation Oncology, National Cancer Center Hospital East, Chiba, Japan; 7 Oncology Translational Science., TDCA, Inc., Lexington, MA USA; 8 Oncology Therapeutic Area Unit, TDCA, Inc., Lexington, MA USA

**Keywords:** Cancer immunotherapy, Pharmacology, Cancer microenvironment

## Abstract

Serine/threonine kinase, cell division cycle 7 (CDC7) is critical for initiating DNA replication. TAK-931 is a specific CDC7 inhibitor, which is a next-generation replication stress (RS) inducer. This study preclinically investigates TAK-931 antitumor efficacy and immunity regulation. TAK-931 induce RS, generating senescence-like aneuploid cells, which highly expressed inflammatory cytokines and chemokines (senescence-associated secretory phenotype, SASP). In vivo multilayer-omics analyses in gene expression panel, immune panel, immunohistochemistry, RNA sequencing, and single-cell RNA sequencing reveal that the RS-mediated aneuploid cells generated by TAK-931 intensively activate inflammatory-related and senescence-associated pathways, resulting in accumulation of tumor-infiltrating immune cells and potent antitumor immunity and efficacy. Finally, the combination of TAK-931 and immune checkpoint inhibitors profoundly enhance antiproliferative activities. These findings suggest that TAK-931 has therapeutic antitumor properties and improved clinical benefits in combination with conventional immunotherapy.

## Abstract (web summary)

TAK-931 is a selective CDC7 inhibitor and can induce replication stress (RS)-mediated chromosomal instability with antitumoral activity. Here the authors show that TAK-931-induced RS generates aneuploid cells with an inflammatory phenotype, rendering tumors sensitive to immune-checkpoint blockade.

## Introduction

Replication stress (RS) is one of the central cancer hallmarks. It is defined as the inefficient DNA replication of the stalled fork progression, mechanistically generated by an incomplete fork structure due to reduced levels of nucleotides and/or replication factors, misincorporation of nucleotides, template DNA lesions, defects in unwinding, or perturbations in DNA structure^1,2^. In addition to these direct effects on the fork structure, several oncogenic activations can also generate RS: MYC amplification/activation, RB mutations, E2F activation, p53 mutations, and cyclin E overexpression^1,3–6^. Conversely, RS stimulates various stress response pathways. RS prominently involves chromosomal instability (CIN), resulting in aneuploidy, through chromosome missegregation, anaphase bridges, and micronuclei^7–9^, and this RS-induced CIN consequently asserts intensive stress responses to RS, mitotic stress, proteotoxic stress, metabolic stress, and others, termed CIN-mediated stress responses. Collectively, RS serves as a central player of cancer hallmarks and vulnerabilities; thus, RS-targeting cancer therapy has gained popularity^1,10^. Conventional chemotherapeutic drugs target RS as the current standard of care in a broad range of cancer types^1^. Novel therapies targeting replication regulatory molecules, such as PARP, ATR, CHK1, and CDC7, are being energetically developed for the next generation of RS-inducing cancer drugs^11–13^.

As a next-generation RS-inducer candidate, the first orally active cell division cycle 7 (CDC7)-selective inhibitor, TAK-931, has been successfully developed^14–17^. TAK-931 intensively induces RS-mediated CIN, consequently leading to apoptosis and cell death, and exhibited a broad spectrum of antiproliferation activities in various preclinical cancer models^14^. TAK-931 induces RS more intensively in *KRAS*-mutant cells, and spontaneous KRAS-driven oncogenic RS may exert synthetic lethality with TAK-931-induced external RS^14^. Recent studies, however, have revealed that the CDC7 inhibitor-induced RS can generate mTOR-driven senescence cells in p53-mutant liver cancers^18^. These findings indicate that CDC7 inhibition-induced RS can exert two distinct antiproliferative phenotypes depending on the cellular context: cell death/apoptotic phenotype and senescence-like phenotype. As senescence-associated secretory phenotype (SASP) is composed of high levels of a group of inflammatory cytokines and chemokines, termed inflammageing^19^, the CDC7 inhibitors may not only assert intracellular antitumor efficacy (cell death and/or apoptotic activity) but also boost antitumor immunity (inflammatory activity).

Immune-checkpoint inhibitors (ICIs), including monoclonal antibodies against programmed cell death protein 1 (PD-1), programmed cell death-ligand 1 (PD-L1), and cytotoxic T lymphocyte-associated protein 4 (CTLA-4), have achieved great advances in cancer therapies for immunologically activated tumors (also called inflamed or hot tumors) across multiple cancer types^20–23^. However, a large proportion of cancers as non-inflamed tumors (also called cold tumors) are still refractory or poorly responsive to ICIs^24^. Combination therapies are a promising strategy to clinically improve ICI therapies, as identification of the best possible combination partner potentially expands which tumors respond. As represented by PARP inhibitors, RS inducers are being evaluated preclinically and clinically for their therapeutic potential as ICI combination partners and are theoretically expected to stimulate immunological activity in tumors and the tumor microenvironment (TME)^13^.

In this study, we demonstrate that the CDC7 inhibitor TAK-931 generates immunologically activated aneuploid cells, which is validated by integrating multiple mechanistic and comprehensive transcriptome analyses. We also conduct multilayer-omics analyses in a syngeneic mouse model, which include flow cytometry (FACS)-based immune profiling panel studies, immunohistochemistry (IHC), bulk RNA sequencing (RNA-seq), and single-cell RNA-seq (scRNA-seq) to evaluate the inflammatory effects of TAK-931 on tumor-infiltrating immune cells (TIICs) in TME. Finally, combination treatment with TAK-931 and ICIs (anti-mPD-1, anti-mPD-L1, and anti-mCTLA-4 antibodies) is confirmed to enhance antiproliferative activities in the preclinical syngeneic mouse model. These findings suggest that TAK-931 can generate immunologically activated TME and potentiate ICIs in combination.

## Results

### CDC7 inhibitor-induced RS generates aneuploid cells with the senescence-associated secretory phenotype

**Fig. 1:**
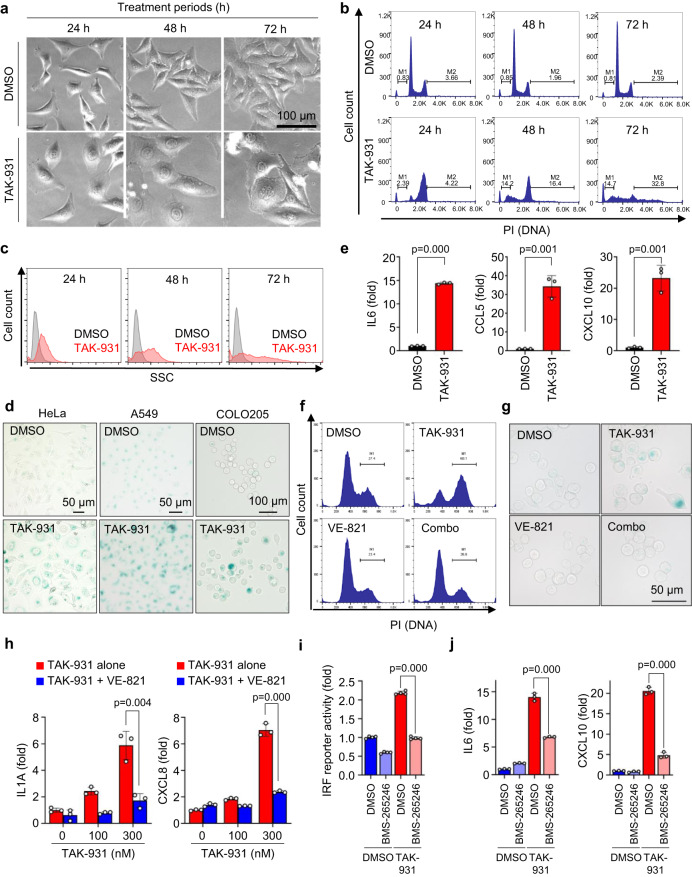
CDC7 inhibitor-mediated aneuploid cells exhibit senescence phenotype. **a** Phase-contrast microscopy images of TAK-931-treated HeLa cells (scale bar = 100 μm). Representative images from *n* = 3 independent experiments with *n* = 5 technical replicates for each experiment are shown. **b** Representative histograms from cell cycle analysis of TAK-931-treated HeLa cells. **c** Side-scatter histograms of TAK-931-treated HeLa cells. **d** Representative images of SA-βGAL staining of cells after 72 h treatment (HeLa, A549: scale bar = 50 μm, COLO205: scale bar = 100 μm). Representative images from *n* = 3 independent experiments with *n* = 5 technical replicates for each experiment are shown. **e** Quantitative reverse transcription–PCR (qRT–PCR) analysis of SASP genes in TAK-931-treated HeLa cells after 72 h of treatment. Data are presented as mean ± SD (*n* = 3 independent experiments), Two sided Student’s *t* test *p* = 0.000, *p* = 0.001, and *p* = 0.001, respectively. **f** Representative histograms from cell cycle analysis of combination TAK-931 and VE-821 treatment. COLO205 cells were treated with DMSO (upper left), TAK-931 at 300 nM (upper right), VE-821 at 1000 nM (lower left), or the combination (lower right) for 24 h. **g** Representative images of SA-βGAL staining after combination treatment. COLO205 cells were treated with DMSO (upper left), TAK-931 at 300 nM (upper right), VE-821 at 1000 nM (lower left), or the combination (lower right) for 24 h (scale bar = 50 μm). Representative images from *n* = 3 independent experiments with *n* = 5 technical replicates for each experiment are shown. **h** qRT–PCR analysis of SASP genes after combination treatment. COLO205 cells were treated with TAK-931 alone (red) or TAK-931 + VE-821 (blue) at the indicated concentrations for 24 h. Data are presented as mean ± SD (*n* = 3 independent experiments). Two-sided Student’s *t* test *p* = 0.004 and *p* = 0.000, respectively. **i** IRF reporter activity in TAK-931 and BMS-265246 treatments. A549 reporter cells were treated with indicated drug treatments and subjected to IRF-Luc reporter assays. Data are presented as mean ± SD (*n* = 4 independent experiments). Two-sided Student’s *t* test *p* = 0.000. **j** qRT–PCR analysis of IL6 and CXCL10 after combination treatment. A549 reporter cells were treated with the indicated drug treatments. Data are presented as mean ± SD (*n* = 3 independent experiments). Two-sided Student’s *t* test *p* = 0.000 and *p* = 0.000, respectively. Source data are provided as a Source Data file.

Recent studies have established the CDC7-specific small-molecule inhibitor, TAK-931, as a clinical candidate that exhibits RS-induced antiproliferation in a broad range of cancer cells^14,15^. To investigate the novel aspects of the RS-induced antiproliferative effects by TAK-931, we first evaluated the time-dependent morphological changes in TAK-931-treated HeLa cells, which were primarily used for the in vitro experiments owing to their ease of use in cell biology analyses^25^. The TAK-931-treated HeLa cells were flattened and enlarged 24 h after treatment, and their cytomegalic phenotype progressed until 72 h of treatment, with an enlarged single nucleus or a grape shape of the multinuclear cluster (Fig. 1a and Fig. S1a). The cell cycle analysis also revealed that, while dimethyl sulfoxide (DMSO; control) treatment exhibited the two-peaked 2N-4N DNA content histograms of cell cycling, the 24–48 h TAK-931 treatment shifted the DNA content to the one-peaked ~4 N DNA content histogram of stalled S and G2/M phases (Fig. 1b)^14,15^. Upon 72 h treatment, the 2-peaked histograms were remarkably disrupted, showing a broader DNA content spectrum outside the 2N–4 N range; the number of cells with a DNA content of 1N–2 N (M1) or 4N–8 N (M2), suggesting induction of aneuploidy, was significantly increased in a time-dependent manner (Fig. S1b)^14,15^.

Considering that senescent cells also exhibit a flattened cytomegalic phenotype, we further assessed whether TAK-931-induced aneuploidy resulted in cellular senescence^26^, which was indicated by internal complexity (i.e., granularity) by side-scatter (SSC), senescence-associated β-galactosidase (SA-βGAL) activity, or SASP-associated inflammatory gene expression. FACS revealed that SSC was higher in the TAK-931-treated cells and increased in a time-dependent manner (Fig. 1c and Fig. S1c). TAK-931 treatment also remarkably increased SA-βGAL activity in HeLa, A549, and COLO205 cells (Fig. 1d and Fig. S1d). According to real-time PCR, the SASP-associated inflammatory gene expression levels of the following were significantly elevated in the TAK-931-treated HeLa cells: *IL6*, *CCL5*, *CXCL10*, *CXCL8*, *CXCL1*, *IL1A*, *IL1B*, *TNFSF15*, and *TNFRSF1B* (Fig. 1e and Fig. S1e). Treatment with another CDC7 inhibitor XL413 also induced SA-βGAL-positive flattened and enlarged phenotype in A549 cells (Fig. S1f).

To confirm that TAK-931-induced upregulation of SASP-associated genes is RS-mediated, we next assessed the induction of the SASP-associated genes under a combination treatment with TAK-931 and the ATR inhibitor VE-821; the ATR–Chk1 DNA damage pathway is involved in RS-mediated antiproliferation in TAK-931 treatment^14^. COLO205 cells were used for the combination with VE-821 because these cells were previously validated in the combination studies^14,15^. VE-821 treatment significantly bypassed TAK-931-induced S/G2 accumulation, significantly recovering cell viability in COLO205 cells (Fig. 1f and Fig. S2a)^14^. Accordingly, TAK-931-induced SA-βGAL activity was also suppressed in the presence of VE-821 in these cells (Fig. 1g). Real-time PCR revealed that the combination treatment with VE-821 significantly suppressed expression levels of the SASP markers, such as IL1A, CXCL8, and CXCL10, in the TAK-931-treated COLO205 cells (Fig. 1h and Fig. S2b, c).

Next, we also assessed the involvement of chromosomal missegregation in TAK-931-induced inflammatory activation. A549-based interferon regulatory factor (IRF)-Lucia luciferase (Luc) reporter cells were used to monitor the activation of the inflammatory pathways in TAK-931 treatment. A CDK1/2 inhibitor, BMS-265246^27^, was used in combination with TAK-931, which is expected to suppress TAK-931-mediated chromosomal missegregation by breaking mitotic entry. Since chromosomal instability was elevated in 48 to 72 h TAK-931 treatment (Fig. 1b), sequential combination treatment was performed: 48 h single-agent TAK-931 treatment followed by 24 h combination treatment with BMS-265246 (Fig. S2d). In the IRF reporter assay, TAK-931 single-agent treatment elevated IRF-Luc activities by 2.2-fold, while combination treatment with BMS-265246 suppressed IRF-Luc activities down to the baseline level (0.99-fold) (Fig. 1i). Real-time PCR also revealed that TAK-931-induced IL6, CXCL10, CCL5, and INFB expressions were significantly suppressed in combination with BMS-265246 (Fig. 1j and Fig. S2e, f). The TAK-931-treated cells, however, did not significantly increase phospho-IRF3, phospho-TBK1, or the nuclear accumulation of cGAS, which are the markers for the cGAS-STING cytosolic DNA-sensing pathway (Fig. S3a, b). In A549-based NF_k_B and IRF dual reporter cells, furthermore, TAK-931 treatment equivalently elevated NF_k_B reporter activity between STING knock-out (KO), negative KO, and no CRISPR (Fig. S3c, d). These results suggest that TAK-931-induced inflammatory activation may not or may only limitedly involve the cGAS–STING pathway (Fig. S3d). Taken together, our findings strongly suggest that CDC7 inhibition by TAK-931 generates RS-mediated cellular senescent aneuploid cells, which highly express inflammatory cytokines and chemokines (SASP), and the ATR–Chk1 and CDK1/2 pathways appear to function as a gatekeeper to trigger TAK-931-induced inflammatory gene expression in these RS-mediated aneuploid cells.

It is still unclear whether TAK-931-induced aneuploid formation could drive inflammation independently of RS or not. We next assessed the aneuploid effects more directly using three distinct mitotic inhibitors as reference: Alisertib (Aurora-A inhibitor), AZD-1152 (Aurora-B inhibitor), and BAY-1217389 (TTK inhibitor), which should cause RS-independent aneuploidy by mitotic aberrations. In HeLa and dual reporter A549 cells, treatment with AZD-1152 and BAY-1217389 exhibited multiple micronuclei beside aberrant shapes of primary nuclei, while treatment with Alisertib did enlarged shapes of primary nuclei with less micronucleus, a similar morphology to TAK-931-treated cells (Fig. S4a, b). FACS analyses confirmed mitotic accumulation in Alisertib treatment (Fig. S4c, d). Alisertib treatment significantly elevated both IRF and NFkB activities, which were equivalent to TAK-931 treatment (Fig. S4e). Real-time PCR also revealed that Alisertib treatment significantly increased CCL5 and CXCL8 expressions (Fig. S4f). Our data demonstrate that RS-mediated aneuploid formation appears to be a key biological event linking to TAK-931-induced cellular senescence, while it is still unclear which, aneuploidy or RS, dominantly and/or more directly drives TAK-931-mediated senescence due to technical limitations: signaling pathways in aneuploidy and RS closely crosstalk each other. Further mechanistic analyses are needed to clarify key drivers of TAK-931-mediated cellular senescence.

Since PARP inhibitors have been reported to immunologically activate TME through innate immune response^28^, we also assessed TAK-931-induced inflammatory activation compared to a PARP inhibitor, Olaparib, as an RS-inducer benchmark. A549-based IRF-Luc reporter cells were used to monitor the activation of the inflammatory pathways. TAK-931 treatment at 100 and 1000 nM significantly elevated IRF-Luc activities by 2.1-fold and 2.9-fold, respectively (Fig. S5a), while Olaparib required a high-concentration treatment (10,000 nM) to reach abundant levels of IRF activity, revealing that TAK-931 induced inflammatory activation at 10-30 times lower concentration than Olaparib did in A-549 cell line model (Fig. S5d). Since BRCA2-deficient cells are highly sensitive to Olaparib^29,30^, we next evaluated TAK-931- and Olaparib-induced inflammatory effects in HeLa cells treated with BRCA2 siRNA (siBRCA2) or control siRNA (siNS). As shown in Supplementary Figure 5c, Olaparib treatment significantly elevated CXCL10 expression in siBRCA2 cells, but not in siNS cells. TAK-931 treatment, on the contrary, equivalently elevated CXCL10 expression in both siNS and siBRCA2 HeLa cells, suggesting that the spectrum of inflammatory TAK-931 would be distinct from that of the PARP inhibitor. Combination treatment with TAK-931, furthermore, enhanced Olaparib-induced IRF activation even at high-concentration Olaparib treatment (Fig. S3d).

### Inflammatory pathways are transcriptionally upregulated in TAK-931-induced aneuploid cells

**Fig. 2:**
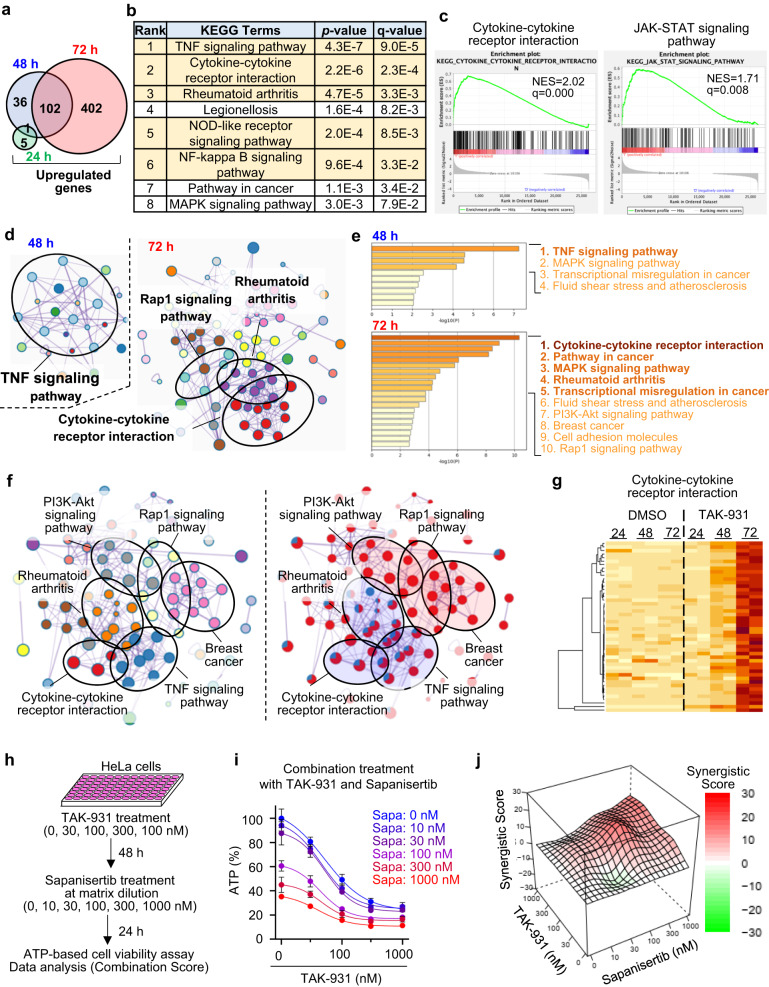
Transcriptome analyses in TAK-931-treated HeLa cells. **a** Venn diagrams of upregulated genes in TAK-931-treated cells. HeLa cells were treated with DMSO or TAK-931 for 24 h (green), 48 h (blue), or 72 h (red). The upregulated genes (>1.5 of log_2_[TAK-931/DMSO]) were determined at each time point. **b** Top eight enriched KEGG terms in 72 h TAK-931-treated HeLa cells. Orange indicates inflammatory cytokine-related pathways. KEGG pathway analysis using DAVID, one-sided p-values for Fisher’s Exact test is adopted to measure the gene-enrichment in annotation terms. **c** GSEAs of representative inflammation-related terms. The RNA-seq data of HeLa cells after 72 h treatment with DMSO or TAK-931 were used. **d** Subextractor network analysis of the upregulated genes in 48 h (left) and 72 h (right) TAK-931-treated HeLa cells. The network was visualized using Cytoscape (v3.1.2). **e** The enrichment network colored by the *p*-values of upregulated genes in 48 h (upper) and 72 h (lower) TAK-931 treatments. **f** Cross-network analysis of upregulated genes between 48 h and 72 h TAK-931 treatments. **g** Heatmaps of individual genes in the enriched cytokine-cytokine receptor interaction pathway. The cells are colored according to their TPM. **h** Experimental schemes for sequential combination treatment with TAK-931 and sapanisertib. **i** Growth inhibition curves in combination treatment with TAK-931 and sapanisertib in HeLa cells at the indicated concentrations. Data are presented as mean ± SD (*n* = 4 independent experiments). **j** 3D plots illustrating Loewe Additivity in combination treatment with TAK-931 and sapanisertib. Positive and negative synergistic scores were colored by red and green, respectively. Source data are provided as a Source Data file.

Transcriptome analyses were also conducted to capture the signaling networks comprehensively in the TAK-931-induced senescent aneuploid cells. HeLa cells were treated with DMSO or TAK-931 (300 nM) for 24 h, 48 h, or 72 h and then subjected to RNA sequencing (RNA-seq) by NovaSeq 6000 (Illumina Inc., San Diego, CA, USA). In comparison analyses at each time point, the expression of 6, 138, and 504 genes were upregulated in TAK-931-treated cells in the 24, 48, and 72 h treatments, respectively (log2[fold] >1.5); 1 and 102 upregulated genes overlapped between the 24 and 48 h and between the 48 and 72 h treatments, respectively (Fig. 2a). The 504 upregulated genes in the 72-h treatment group were subjected to enrichment analyses in the Kyoto Encyclopedia of Genes and Genomes (KEGG) database, revealing that the inflammatory cytokine and chemokine hallmarks were significantly and intensively enriched in the TAK-931-treated cells; five out of the top six KEGG pathways were inflammatory-related (Fig. 2b). The gene set enrichment analysis (GSEA) also confirmed significant enrichment of these inflammatory pathways upon 72 h TAK-931 treatment: cytokine-cytokine receptor interaction, NOD-like receptor signaling pathway, JAK-STAT signaling pathway, and cytosolic DNA-sensing pathway (Fig. 2c and Fig. S6a, b).

To visualize the enrichment network modules, Metascape enrichment analyses were performed at each time point (https://metascape.org/). The results showed that 48-h treatment upregulated 138 genes and 72-h treatment upregulated 504 genes. In the Metascape analyses, the TNF signaling pathway was a dominant network module in the 48-h treatment group, while the inflammatory-related modules were more intensively accumulated, and the signaling networks expanded to the surrounding various modules in the 72-h treatment, (Fig. 2d, e, and Fig. S6c, d). To visualize the time-dependent changes of the network modules, integration analyses on the genes upregulated at 48 and 72 h were also performed: the representative signaling modules (Fig. 2f, left), 48-h and 72-h pie charts (Fig. 2f, right), and enrichment scores (Fig. S6e). The integration analyses also demonstrated that the inflammatory modules, such as cytokine-cytokine receptor interaction, TNF signaling pathway, and rheumatoid arthritis, were progressively enriched in the TAK-931-treated cells. These time-dependent enrichments were also confirmed using the individual gene expression heatmap (Fig. 2g and Fig. S7a, b). A number of the additional surrounding modules, such as the mTOR-related signaling modules, were also significantly enriched in the group subjected to 72 h treatment (Fig. S7c). Considering that mTOR pathways are crucial in cellular senescence^31^, we also conducted in vitro combination studies with TAK-931 and a mTORC1/2 inhibitor sapanisertib^32^ at matrix dilutions: 48 h single-agent TAK-931 treatment followed by an additional 24 h combination treatment (Fig. 2h, i and Fig. S7d). Combination with sapanisertib enhanced antiproliferation in TAK-931-induced senescent cells (Fig. 2i, j), which is also consistent with the previous studies of senolytic activity of mTOR and CDC7 inhibitors^18^. Taken together, our signaling module results indicate that the TAK-931-induced cellular senescence progressed in a time-dependent manner, and mTOR pathways play important roles in viability for TAK-931-induced senescent cells^18^

### CDC7 inhibitor-induced aneuploid cells exhibited distinct changes in the transcriptional landscape in single-cell RNA sequencing

**Fig. 3:**
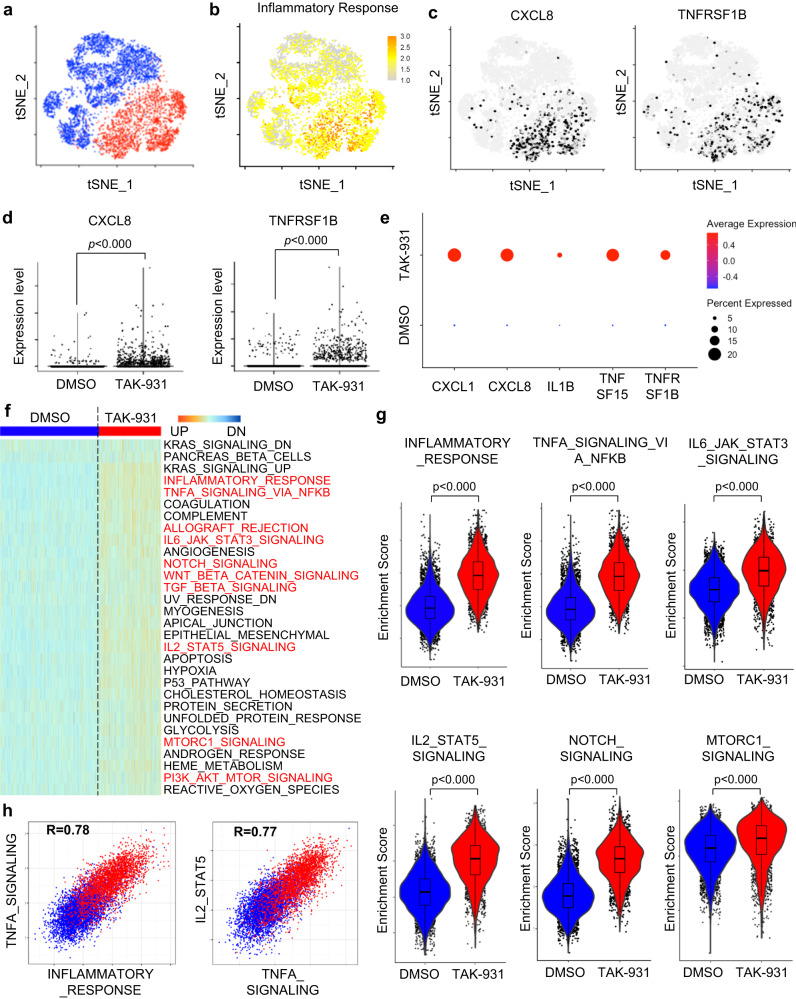
Single-cell transcriptional landscape in TAK-931-treated aneuploid cells. **a** tSNE plot based on the gene expression of DMSO- or TAK-931-treated HeLa cells. Clusters of 72 h DMSO-treated (blue) and TAK-931-treated (red) HeLa cells are shown. **b** Enrichment of inflammatory response hallmark in tSNE plots. Enrichment scores of the hallmark are colored yellow to orange. **c** Inflammatory gene expression in tSNE plots. Cells expressing CXCL8 (left) and TNFRSF1B (right) are colored in black (>1tag). **d** Dot plots of expression levels of *CXCL8* (left) and *TNFRSF1B* (right) in single cells administered DMSO (blue) or TAK-931 (red). Plots are two independent experiments. Two-sided Welch Two Sample *t* test *p* < 0.000, *p* < 0.000, respectively. **e** Circle plots of the expressions of inflammatory-related genes *CXCL1, CXCL8, IL1B, TNFSF15*, and *TNFRSF1B* in DMSO- or TAK-931-treated cells. The circle color and size indicate average expression and expression percentage, respectively. **f** Heatmap of the enrichment scores, based on ssGSEA, between DMSO-treated (left) and TAK-931-treated (right) cells. Red indicates inflammatory-related hallmarks. **g** Violin boxplot of enrichment scores of inflammatory response, TNFA signaling pathway via NFkB, IL6-JAK-STAT3 signaling, IL2-STAT5 signaling, Notch signaling, and mTORC1 signaling in DMSO-treated (blue) and TAK-931-treated (red) cells. Plots are two independent experiments. All violin boxplots are defined by center lines (medians), box limits (25th and 75th percentiles), and whiskers (minima and maxima; the smallest and largest data range). Two-sided Welch Two Sample *t* test *p* < 0.000, respectively. **h** Comparison of enrichment scores between TNFA signaling and inflammatory response (left), between IL2-STAT5 signaling and TNFA signaling (right). DMSO-treated and TAK-931-treated cells are shown in blue and red, respectively. Source data are provided as a Source Data file.

We also performed single-cell RNA sequencing (scRNA-seq) analyses to determine the transcriptional landscape of the TAK-931-induced aneuploid cells at the single-cell levels. HeLa cells were treated with DMSO or TAK-931 (300 nM) for 72 h and then subjected to the microdroplet-based platform (10X Genomics) of scRNA-seq by NovaSeq 6000 (Illumina Inc.). Passing the quality control check, 3998 cells in the DMSO treatment group and 2479 cells in the TAK-931 treatment group were subjected to the following analyses, where the mean numbers of unique genes detected were 7103 and 7787, respectively. In the T-distributed stochastic neighbor embedding (t-SNE) plots, the DMSO- and TAK-931-treated cells were classified in two distinct clusters (Fig. 3a, DMSO in blue and TAK-931 in red), indicating that the major population of the TAK-931-treated aneuploid HeLa cells was transformed to a cell contexture that was transcriptionally distinct from that of DMSO-treated control cells; this was concordant with the morphological changes to the cytomegalic SASP cells (Fig. 1). Supporting the results in bulk RNA-seq analyses (Fig. 2), the hallmark of an inflammatory response was remarkably enriched in the TAK-931 treatment cluster (Fig. 3b). In addition, the positive cells of inflammatory cytokine/chemokine-related genes, such as *CXCL8, IL1B, TNHSF14* and *TNFRSF1B*, were significantly accumulated in the TAK-931-treatment cluster (Fig. 3c, d and Fig. S8a, b), in which both average and percent expression were remarkably increased in the TAK-931-treated cells (Fig. 3e). The single-sample-based gene signature enrichment analyses (ssGSEA) also demonstrated that the numbers of inflammatory cytokine/chemokine-related hallmarks as well as the mTOR-associated SASP hallmarks (e.g., inflammatory response, TNFA signaling via NF-κB, allograft rejection, IL6-JAK STAT3 signaling, and IL2-STAT5 signaling, mTORC1 signaling, and PI3K AKT mTOR signaling) were enriched in the TAK-931-treated cells (Fig. 3f highlighted in red). The violin plots of the enrichment scores also confirmed that the inflammatory cytokine/chemokine-related hallmarks and mTOR-associated SASP hallmarks were significantly enriched in TAK-931-treated cells (Fig. 3g). The scatter plots revealed that these enriched pathways were collaterally upregulated in response to TAK-931 treatment (Fig. 3h and Fig. S8c), suggesting that the TAK-931-induced aneuploidy may intensively and/or simultaneously activate multiple lines of the inflammatory cytokine/chemokine-related and SASP-associated hallmarks.

**Fig. 4:**
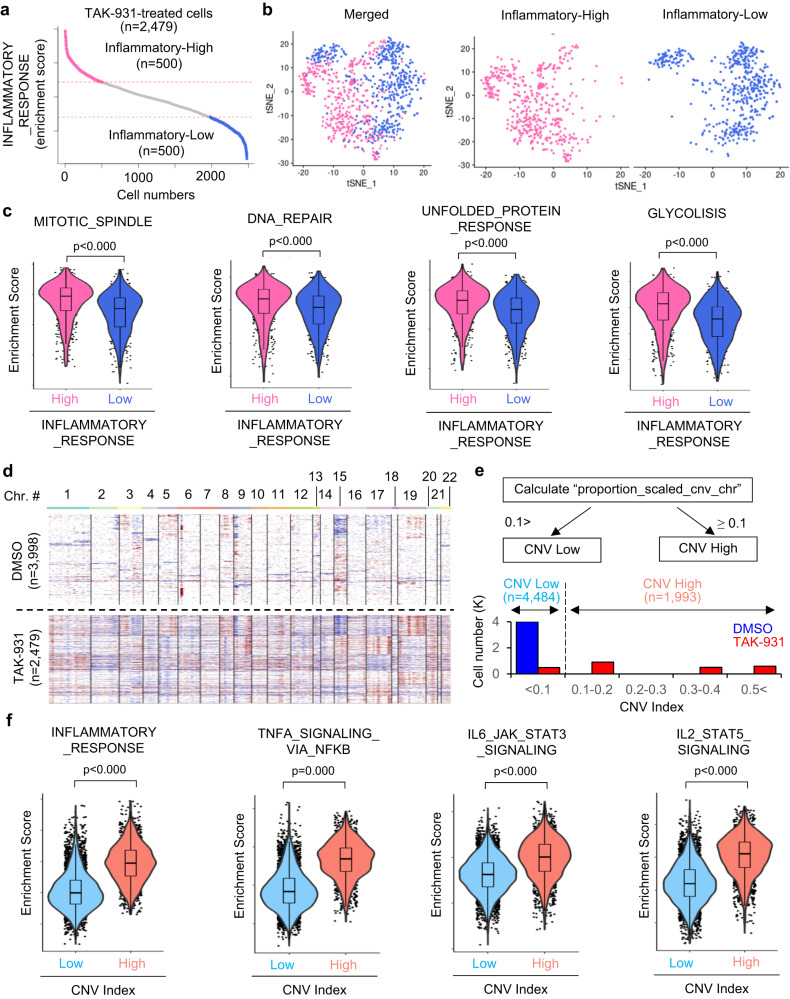
Comparison analyses between the inflammatory-high and -low populations in TAK-931-treated cells. **a** Schemes of comparative analyses between inflammatory-high and -low populations in TAK-931-treated cells. Inflammatory-high and -low cells in the TAK-931 treatment group are shown in pink and sky blue, respectively. **b** tSNE plot based on the gene expression of the inflammatory-high (pink) and -low (sky blue) cells administered TAK-931 treatment. **c** Violin boxplot of enrichment scores in the indicated aneuploid stress-related hallmarks (mitotic spindle, DNA repair, unfolded protein response, and glycolysis) are shown between inflammatory-high (pink) and inflammatory-low (sky blue) cell groups. Plots are two independent experiments. All violin boxplots are defined by center lines (medians), box limits (25th and 75th percentiles), and whiskers (minima and maxima; the smallest and largest data range). Two-sided Welch Two Sample *t* test *p* < 0.000, respectively. **d** InferCNV analyses in HeLa cells treated with DMSO or TAK-931. The heatmap of copy number variation (CNV) in DMSO-treated (upper) and TAK-931-treated (lower) cells is shown. Red and blue indicate increased and decreased chromosomal copy numbers, respectively. **e** Schemes of comparative analyses between CNV-high (light orange) and -low (light blue) populations. **f** Violin boxplot of enrichment scores in inflammatory response, TNFA signaling pathway via NF-κB, IL6-JAK-STAT3 signaling, and IL2-STAT5 signaling between CNV-high (light orange) and CNV-low (light blue) cell groups are shown. Plots are two independent experiments. All violin boxplots are defined by center lines (medians), box limits (25th and 75th percentiles), and whiskers (minima and maxima; the smallest and largest data range). Two-sided Welch Two Sample *t* test *p* < 0.000, respectively. Source data are provided as a Source Data file.

To characterize TAK-931-induced pathways associated with increased inflammation, we investigated differences in gene regulation in TAK-931-treated cells subdivided by the level of the inflammatory signature. Specifically, using scRNA-seq data, we classified TAK-931-treated HeLa cells into two groups based on the enrichment scores of the Inflammatory_Response KEGG term: the top-500 cells for the inflammatory-high group and the bottom-500 cells for the inflammatory-low group (Fig. 4a). In the t-SNE plots, the inflammatory-high and -low TAK-931-treated cells were classified into transcriptionally distinct clusters (Fig. 4b). In ssGSEA analyses, as expected, the multiple inflammatory-associated KEGG pathways, such as TNFα signaling via NFκB, IL6-JAK STAT3 signaling, and IL2-STAT5 signaling, were significantly enriched in the inflammatory-high TAK-931-treated cells. In addition to this expected enrichment in the inflammatory-related hallmarks, mitotic stress hallmarks (e.g., Mitotic Spindle), RS hallmarks (e.g., G2M Checkpoint and DNA Repair), proteotoxic stress hallmarks (e.g., Unfolded Protein Response), and metabolic-stress hallmarks (e.g., Glycolysis, Fatty Acid Metabolism, and Cholesterol Homeostasis) were significantly enriched in the inflammatory-high population (Fig. 4c and Fig. S9a, b). As mitotic stress, RS, proteotoxic stress, and metabolic stress have been reported to be four major hallmarks of aneuploid-associated cellular stress^33^, these results suggest that more extensive aneuploidy leads to enhanced inflammatory- and SASP-associated pathways in TAK-931-induced aneuploid cells.

We further investigated the involvement of TAK-931-induced aneuploidy in the activation of inflammatory- and SASP-associated pathways by employing transcriptome-based copy number variation (CNV) analyses of scRNA-seq data (InferCNV), which infers somatic large-scale CNV at a single-cell level: gains or deletions of entire chromosomes or large segments of chromosomes (https://github.com/broadinstitute/inferCNV)^34,35^. Supporting the broader spectrum of DNA content in FACS analyses (Fig. 1b), the heatmap of InferCNV also showed that the CNV were intensively promoted in TAK-931-treated HeLa cells (Fig. 4d). Next, the scaled proportion of CNV (CNV index) in each cell was calculated by Seurat, and then the cells were categorized into two groups based on CNV index scores: cells at <0.1 and ≥0.1 CNV indexes were categorized as CNV-low and CNV-high cells, respectively (Fig. 4e). Specific gain or loss of copy numbers in TAK-931-treated HeLa cells were identified at Chr-1, 3, 9, 17 and 19 in copy number gain, while Chr-9 and 15 in copy number loss (Fig. S10a, b). In the t-SNE plots, the CNV-low and CNV-high cells were localized into transcriptionally distinct clusters (Fig. S10c). The heatmaps of ssGSEA analyses revealed that the inflammatory-associated hallmarks were intensively enriched in the CNV-high cells (Fig. S10d), and the violin plots of the enrichment scores also confirmed that the following inflammatory cytokine/chemokine-related hallmarks were significantly enriched in the CNV-high cells compared with those in the CNV-low cells: inflammatory response, TNFα signaling via NFκB, and IL6-JAK-STAT Signaling, and IL2-STAT5 Signaling (Fig. 4f). Taken together, these findings indicate that TAK-931-induced RS generates immunologically activated aneuploid cells, which could potentially induce immunological cold-to-hot tumor conversion.

### Peripheral blood mononuclear cells are recruited to TAK-931-treated A549 cells in vitro

**Fig. 5:**
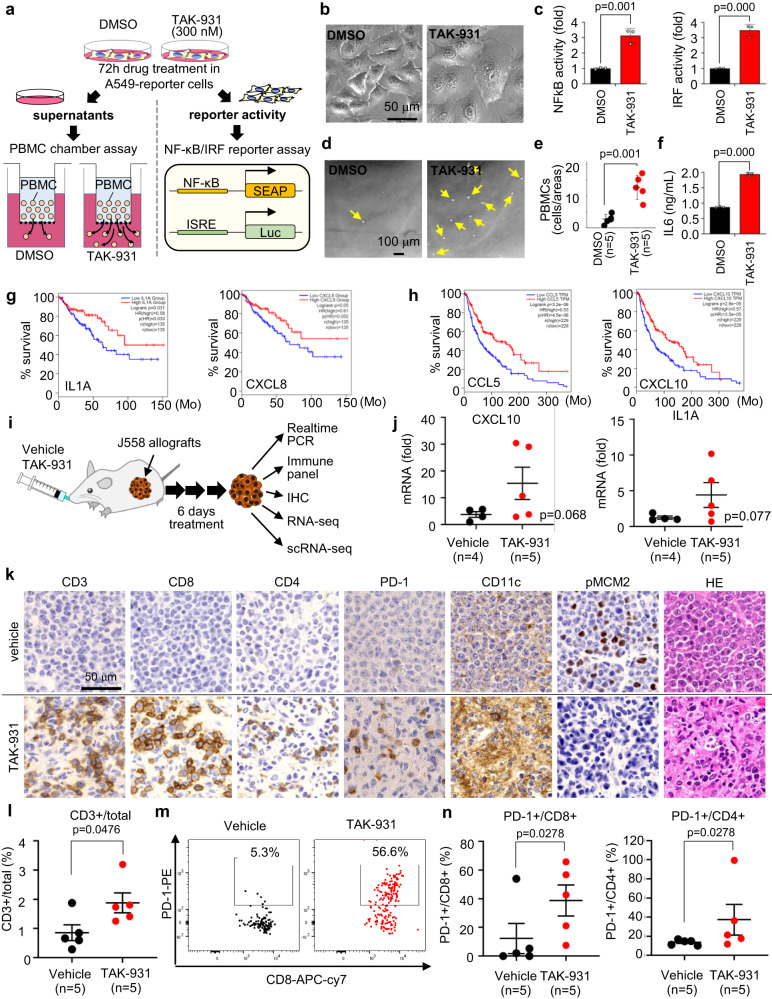
Effects of TAK-931 on tumor infiltration of immune cells. **a** Experimental schemes of PBMC migration assays and dual reporter assays. **b** Phase-contrast microscopy images of DMSO-treated (left) and TAK-931-treated (right) A549 cells (scale bar = 50 μm). Representative images from *n* = 3 independent experiments with *n* = 5 technical replicates for each experiment are shown. **c** A549 cells were treated with TAK-931 for 72 h and subjected to NF-κB-SEAP (left) and IRF-Luc (right) dual reporter assays. Black and red bars indicate DMSO and TAK-931 treatments, respectively. Data are presented as mean ± SD (*n* = 3 independent experiments). Two-sided Student’s *t* test *p* = 0.001 and *p* = 0.000, respectively. **d** Phase-contrast microscopy images of membrane-through PBMCs (indicated by yellow arrows) to the conditioned medium of DMSO-treated (left) or TAK-931-treated (right) A549 cells (scale bar = 100 μm). Representative images from *n* = 3 independent experiments with *n* = 5 technical replicates for each experiment are shown. **e** Quantitative analyses of membrane-through PBMCs to the conditioned medium of DMSO-treated (black) or TAK-931-treated (red) A549 cells. Data are presented as mean ± SD (*n* = 5 independent experiments). Two-sided Student’s *t* test *p* = 0.001. **f** IL-6 secretion from DMSO- (black) or TAK-931-treated (red) A549 cells. Data are presented as mean ± SD (*n* = 3 independent experiments). Two-sided Student’s *t*-test *p* = 0.000. **g**, **h** Overall survival based on inflammatory-related gene expression. Differences in overall survival between high (red) and low (blue) expression of the indicated inflammatory-related genes were analyzed in colon adenocarcinoma **g** and skin cutaneous melanoma **h**. **i** Experimental schemes of in vivo multilayer-omics analyses in J558-allograft model. **j** Quantitative reverse transcription–PCR analysis of CXCL10 and IL1A in the J558-allograft model in treatment with vehicle (black, *n* = 4) and TAK-931 (red, *n* = 5). Data are presented as mean ± SD. One-sided Student’s *t* test *p* = 0.068 and *p* = 0.077, respectively. **k** Immunohistochemistry of the immune markers in the tumor sections from J558-allograft mice after oral administration of vehicle (upper) or TAK-931 (lower) for 6 days (scale bar = 50 μm). Immunohistochemistry images are a representative example of 4 individual J558-allograft tumors from one experiment. **l** Quantitative analyses of CD45^+^CD3^+^ T cells in J558 allografts. Percentages of CD45^+^CD3^+^ T cells of the total cells are shown. Vehicle (*n* = 5) and TAK-931 (*n* = 5) treatments are shown in black and red, respectively. Data are presented as mean ± SD. One-sided non-parametric Wilcoxon–Mann–Whitney test *p* = 0.0476. **m** Representative FACS staining of PD-1 + CD8+ cytotoxic T cells in vehicle-treated (left) and TAK-931-treated (right) J558 allografts. **n** Quantitative analyses of PD-1^+^CD8^+^ cytotoxic T cells and PD-1^+^CD4^+^ helper T cells in J558 allografts. Vehicle and TAK-931 treatments are shown in black and red, respectively. Data are presented as mean ± SD (*n* = 5 independent experiments). One-sided non-parametric Wilcoxon–Mann–Whitney test *p* = 0.0278, *p* = 0.0278, respectively. Source data are provided as a Source Data file.

The effects of TAK-931 on the inflammatory cytokine/chemokine pathways were functionally assessed in peripheral blood mononuclear cell (PBMC) migration assays. NF-κB/ IRF dual reporter assays were also performed in parallel to confirm the activation of the inflammatory pathways in TAK-931-treated cancer cells (Fig. 5a). A549-based dual NF-κB-secreted embryonic alkaline phosphatase (SEAP) and IRF-Lucia luciferase (Luc) reporter cells were used for the reporter assays (InvivoGen, USA). The TAK-931-treated A549 cells also exhibited the flat-shaped cytomegalic phenotype with SA-βGAL activity (Figs. 5b and  1). In the reporter assays, NF-κB-SEAP and IRF-Luc activities were significantly increased by 3.1-fold and 3.5-fold, respectively, in these TAK-931-treated cells (Figs. 1 and 5c), confirming transcriptional activation of the inflammatory pathways of NF-κB and IRF signaling. Next, to assess the in vitro PBMC migration activity, the chamber assay was performed using the conditioned medium of these TAK-931-induced senescent cells (Fig. 5a). The upper and lower compartments in the migration chamber were separated by a porous membrane through which the PBMCs in the upper compartment passed into the lower compartment via recruitment by chemoattractants of inflammatory cytokines/chemokines in the conditioned medium. The chemoattractant activity of the conditioned medium was evaluated by the number of membrane-through PBMCs in the lower chamber. The number of membrane-through PBMCs in the bottom chamber was significantly increased with the conditioned medium of the TAK-931-treated A549 cells (Fig. 5d, e, p < 0.05). The concentration of IL-6 protein in the TAK-931-treated conditional medium was significantly higher than that in the DMSO-treated conditional medium (Fig. 5f). This finding indicated that the TAK-931-induced aneuploid cells appear to be immunologically activated (inflamed), which would attract the PBMCs through the secretory chemoattractants.

The clinical database of Gene Expression Profiling of Interactive Analysis (GEPIA) also revealed that, in the case of colon adenocarcinoma (COAD), the cancers (Fig. 5g, red lines) highly expressing the inflammatory-related genes, *IL1A, IL1B*, and *CXCL8*, significantly exhibited better overall survival ratios compared to that of cancers of low expression of these genes (Fig. 5g, blue lines and Table S1). In skin cutaneous melanoma (SKCM), cancers with high expression of *IL1B*, *CCL5*, *CXCL10*, and *TNFSF15* exhibited better prognoses (Fig. 5h and Table S1). In addition, breast cancers (BRCA) with high expression of CCL5 and TNFSF15 and ovarian cancers (OV) with high expression of CXCL10 exhibited better prognoses (Table S1). Although bulk RNA-seq data did not determine the cellular components expressing these inflammatory-related genes, these results suggest that inflamed tumor microenvironment appears to have better prognoses in these tumor types; thus, TAK-931-induced immunological activation may potentially improve the currently used cancer therapeutics.

### TAK-931 promotes tumor-infiltrating immune cells and cold-to-hot tumor conversion in vivo

To comprehensively evaluate the in vivo inflammatory effects of TAK-931 in TME, multilayer-omics analyses were conducted in the J558 plasma cell myeloma syngeneic mouse model: gene expression by real-time PCR, FCM-based immune profiling panel, IHC, and transcriptome analyses by bulk RNA-seq and scRNA-seq (Fig. 5i). Initially, we evaluated the proliferative effect of TAK-931 in hematopoietic cells (CD45^+^ cells) of peripheral blood mononuclear cells (PBMC) and bone marrow (BM). BALB/c female mice were orally administered with vehicle or TAK-931 at 60 mg/kg, once daily (QD) for 5 days, and CD45+ cells in PBMC or BM were sorted to count % CD45^+^ in each treatment. In FACS analyses, no significant difference in % of CD45^+^ cells in PBMC or BM was detected between vehicle- and TAK-931 administered BALB/c female mice, indicating less proliferative effects of TAK-931 in hematopoietic cells in PBMC or BM (Fig. S11a–d).

The J558-allograft model was primarily used for subsequent in vivo experiments in which efficacy studies are technically valid for evaluating ICI combinations^36,37^. The in vitro assay confirmed that J558 cells were less sensitive to TAK-931, with an IC50 value of >1000 nM (Fig. S11e), while SSC was higher in the TAK-931-treated J558 cells compared to DMSO-treated cells (Fig. S11f, g). The J558-allografted BALB/c female mice were orally administered vehicle or TAK-931 at 60 mg/kg, QD for 6 days for the indicated multilayer-omics analyses (Fig. 5i). The results showed moderate, but significant, antitumor efficacy in the 5- or 6-day TAK-931 treatment (Fig. S12a–c, T/C = 49.7% at day 5). Real-time PCR revealed that CXCL10 and IL1A levels were remarkably elevated in the J558 allografts of TAK-931-treated mice (*n* = 5) compared with those in vehicle-treated mice (*n* = 4) (Fig. 5j). Next, IHC studies were performed in the allografts of TAK-931-treated and vehicle-treated mice. As representatives, allografts of vehicle-1 and TAK-931-1 were used for the following IHC, bulk RNA-seq, and scRNA-seq analyses (Fig. S13). IHC with extracellular immune markers to the classified immune cell types revealed that CD3^+^ cells (T cells), CD8^+^ cells (cytotoxic T cells), CD4^+^ cells (helper T cells), PD1^+^ cells (activated/exhausted T cells), and CD11C^+^ cells (dendric cells) were prominently accumulated in the TAK-931-treated allografts (Fig. 5k). pMCM2, a substrate of CDC7 kinase, was used as a pharmacodynamics marker for TAK-931. To confirm the effects of TAK-931 on tumor infiltration of immune cells in more detail, the dissociated allografts were also subjected to FCM-based immune profiling panel studies, which classified various immune cell types based on extracellular or intracellular immune markers (Fig. S12c): CD45^+^ for hematopoietic cells (except for mature erythrocytes and platelets), CD45^+^CD3^+^ for T cells, CD45^+^CD3^+^CD8^+^ for cytotoxic T cells, and CD45^+^CD3^+^CD4^+^ for helper T cells. The immune panel studies revealed that the percentage of CD45^+^ cells was significantly increased in the TAK-931-treated allograft tumors compared with that in the vehicle-treated tumors (*p* < 0.05, Fig. S12d, e), confirming the recruitment activity of the TAK-931-treated tumors for hematopoietic cells in the TME of the in vivo mouse allografts. The percentage of CD45^+^CD3^+^ tumor-infiltrating lymphocytes (TILs) was significantly increased in the TAK-931-treated allografts as well (*p* < 0.05, Fig. 5l). Furthermore, percentage of activated PD-1^+^CD8^+^ and PD-1^+^CD4^+^ T cells (Fig. 5m, n) were significantly elevated in the TAK-931-treated allografts, indicating that the recruited CD8^+^ and CD4^+^ T cells were subsequently activated in the TAK-931-treated TME. However, the percentage of CD45+ myeloid-derived suppressor cells (MDSCs), which are immunosuppressive, was significantly decreased in the TAK-931-treated allografts (Fig. S12f–i). These findings suggest that TAK-931 could potentially convert a non-inflamed TME to an inflamed TME.

**Fig. 6:**
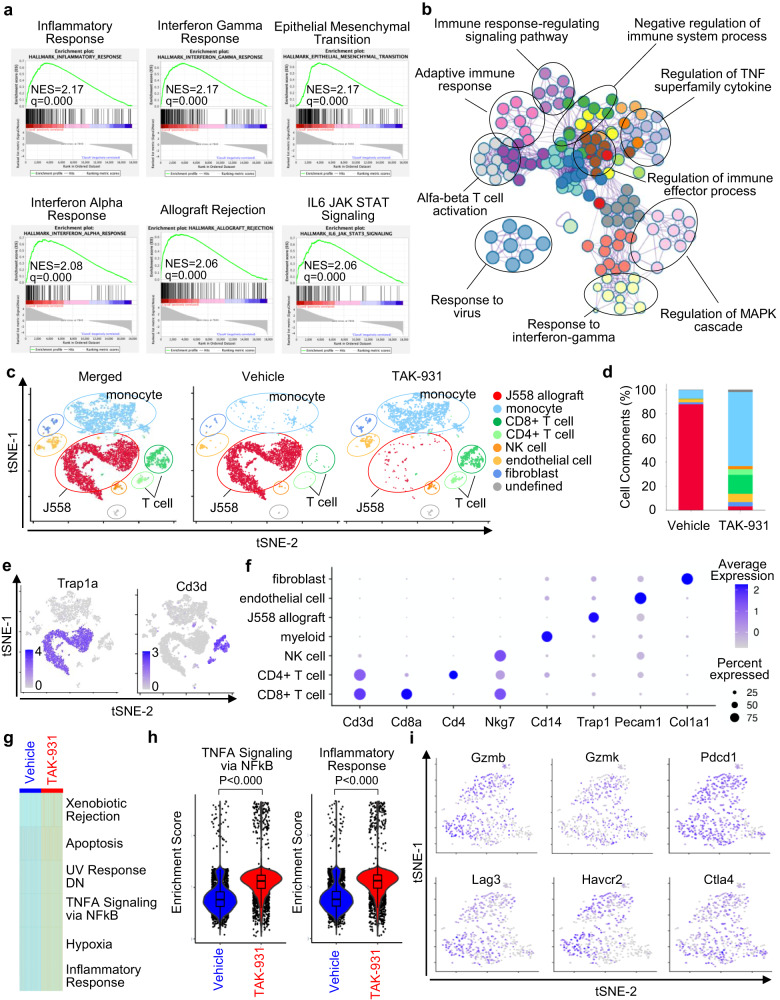
Effects of TAK-931 on the transcriptional landscape of TME in J558 syngeneic mouse model. **a** GSEAs of representative inflammation-related terms in J558 allografts. The RNA-seq data of J558 allografts after 6-days treatment with vehicle (vehicle-1) and TAK-931 (TAK-931-1) were used. **b** Subextractor network analysis of the upregulated genes in TAK-931-treated allografts. The network was visualized using Cytoscape (v3.1.2). **c** tSNE plot based on the gene expression of the allografts after 6 days treatment with vehicle (vehicle-1) and TAK-931 (TAK-931-1). Clusters were classified into the cellular components by the marker genes described in Supplementary Figure 12c. **d** Quantitative data of each cellular component in the allografts in treatment with vehicle and TAK-931. **e** Gene expression plots of the representative marker genes. **f** Circle plots of the expressions of the representative marker genes. The circle color and size indicate average expression and expression percentage, respectively. **g** Heatmap of the enrichment scores, based on ssGSEA, between vehicle-treated (left) and TAK-931-treated (right) allografts. **h** Violin boxplot of enrichment scores of TNFA signaling pathway via NFkB and inflammatory response in vehicle-treated (blue) and TAK-931-treated (red) allografts. Plots are two independent experiments. All violin boxplots are defined by center lines (medians), box limits (25th and 75th percentiles) and whiskers (minima and maxima; the smallest and largest data range). Two sided Welch Two Sample *t*-test *p* < 0.000, respectively. **i** Gene expression tSNE plots in CD8^+^ T cells. CD8^+^ T cells expressing the indicated marker genes are colored in purple. Source data are provided as a Source Data file.

To evaluate the effects of TAK-931 on the transcriptional landscape of TME more comprehensively, transcriptome analyses of both bulk RNA-seq and scRNA-seq were conducted in allografts compared between vehicle-treated and TAK-931-treated mice. The GSEA revealed significant enrichment of inflammatory hallmarks in TAK-931-treated allografts: inflammatory response, interferon-gamma response, epithelial-mesenchymal transition, interferon alfa response, allograft rejection, and IL6-JAK-STAT signaling (Fig. 6a and Fig. S13a). Metascape analyses also visualized that the network modules of the inflammatory signaling pathways were predominantly enriched, such as immune-response-regulating signaling pathway, adaptive immune response, alfa-beta T cell activation, regulation of immune effector process, and response to interferon-gamma (Fig. 6b and Fig. S13b–d). In scRNA-seq, the tSNE plots and the expression dot plots showed cellular components in the allografts classified by the indicated TME markers: cancer cell (J558-allograft), monocyte, CD8 + T cell, CD4 + T cell, NK cell, endothelial cell, fibroblast, and undefined (Fig. 6c–f and Fig. S14a–c), demonstrating that TAK-931 treatment prominently promoted tumor infiltration of both adaptive (CD8^+^ T cell and CD4^+^ T cell) and innate (NK cells and monocytes) immune cells in the allografts. The ssGSEA revealed that the inflammatory cytokine/chemokine-related hallmarks were significantly enriched in TAK-931-treated allografts (Fig. 6g). The violin plots of the enrichment scores also confirmed that the hallmarks of TNFA signaling via NFkB and inflammatory response were significantly upregulated in TAK-931-treated allografts (Fig. 6h). Furthermore, tumor-infiltrated CD8^+^ T and CD4^+^ T cells in the allografts highly expressed the activation/exhaustion markers, such as GZMB, GZMK, PDCD1, LAG3, HAVCR2, and CTLA-4 (Fig. 6i and Fig. S14d). The multilayer-omics analyses strongly suggest that TAK-931-induced RS in cancer cells can convert to the inflamed TME to activate both innate and adaptive immune systems. Further mechanistic analyses need to be conducted to understand more completely the immunological effects of TAK-931 in the TME.

### Single-agent treatments with TAK-931 demonstrate antitumor immunity and efficacy in vivo

**Fig. 7:**
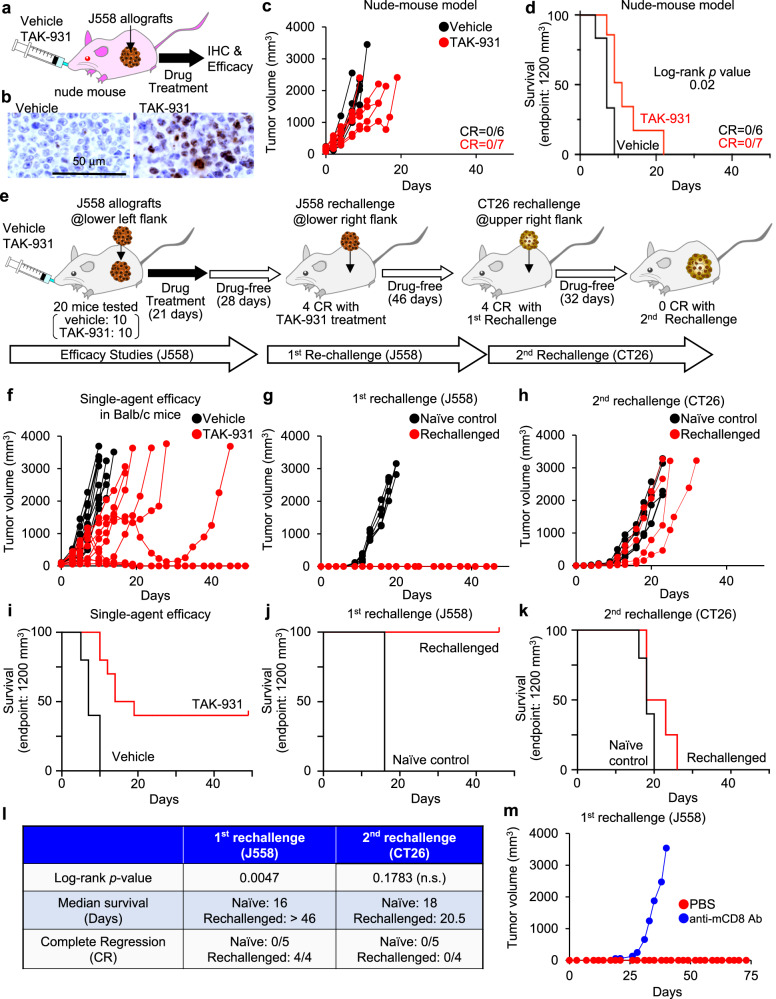
Antitumor efficacy studies using single-agent TAK-931 and tumor-rechallenge studies in the J558 mouse syngeneic model. **a** Experimental schemes of antitumor efficacy studies using single-agent TAK-931 in the J558 nude mouse model. **b** Immunohistochemistry of γH2A/X in the tumor sections from J558-allograft mice after efficacy studies, vehicle (left) for 4 days or TAK-931 (right) for 11 days. Black bars indicate 50 μm. Immunohistochemistry images are a representative example of 8 individual J558-allograft tumors from one experiment. **c** Antitumor efficacy of TAK-931 orally administered at the indicated regimen in the J558-allograft nude mouse model. Black and red indicate vehicle and TAK-931 treatments, respectively. The efficacy data are shown as tumor volumes (mm^3^). **d** Preclinical Kaplan–Meier survival curves at 1200 mm^3^ of the endpoint tumor volume. **e** Experimental schemes of antitumor efficacy studies using single-agent TAK-931 and tumor-rechallenge studies in the J558 mouse syngeneic model. **f** Antitumor efficacy of TAK-931 orally administered at the indicated regimen in the J558-allograft mouse model. Black and red indicate vehicle and TAK-931 treatments, respectively. The efficacy data are shown as tumor volumes (mm^3^); *n* = 10. Vehicle: CR = 0/10, TAK-931: CR = 4/10. **g**–**h** First **g** and second **h** tumor-rechallenge studies in J558 and CT26 mouse syngeneic models, respectively. Black and red indicate naive mice (*n* = 5) and rechallenged CRs (*n* = 4), respectively. Vehicle: CR = 0/5, TAK-931: CR = 4/4 **g**, Vehicle: CR = 0/5, TAK-931: CR = 0/4 **h**. **i**–**k** Preclinical Kaplan–Meier survival curves at 1200 mm^3^ of the endpoint tumor volume. Antitumor efficacy results of TAK-931 in J558-allograft **i**, first-rechallenge studies in J558 **j**, and second-rechallenge studies in CT26 **k** are shown. **l** Summary of rechallenge studies in the J558-allograft model, naive mice (*n* = 5) and rechallenged CRs (*n* = 4). Two sided Log-rank test *p* = 0.0047 (1st rechallenge), *p* = 0.1783 (2nd rechallenge), respectively. **m** Tumor-rechallenge studies with anti-CD8a antibody in J558 mouse syngeneic model. The rechallenged CRs given single-agent TAK-931 treatment were used (*n* = 2). Red and blue lines indicate phosphate-buffered saline (*n* = 1) and anti-CD8a antibody (*n* = 1) treatments, respectively. Data are shown as tumor volumes (mm^3^). Source data are provided as a Source Data file.

We assessed in vivo efficacy of TAK-931 at single-agent treatment compared between immune-deficient nude mouse models and immune-competent BALB/c mouse models. TAK-931 at 60 mg/kg (*n* = 7) or 0.5% methylcellulose (vehicle control, *n* = 6) was orally administered to J558-allografted nude mice once a day until the humane endpoint (Fig. 7a). IHC of pMCM2 and γH2A.X was performed for target-engagement and functional (RS and DNA damage) PD biomarkers, respectively. In TAK-931-treated J558 allografts, pMCM2-positive cells were substantially decreased down to the undetected level, while γH2A.X-positive cells were profoundly increased, indicating that TAK-931-treated J558 allografts were considerably exposed by RS and DNA damage (Fig. 7b and Fig. S15a). In addition, the cells in TAK-931-treated allografts were enlarged compared to those in vehicle control, which appeared to be similar morphology observed in vitro studies (Fig. S15a). The preclinical Kaplan–Meier survival analysis was also conducted with an endpoint tumor volume of 1200 mm^3^ as a surrogate for mortality, revealing that all 6 mice in the vehicle control reached the 1200 mm^3^ endpoint within 9 days (Fig. 7c, d and Fig. S15b, c). In TAK-931 treatment, although significant antiproliferative activity was observed, all 7 mice in TAK-931 treatment also reached the 1200 mm^3^ endpoint in 21 days. All mice tested were confirmed to reach the humate endpoints in 21 days as well (Fig. 7c). These results demonstrated that the antiproliferative activity of TAK-931 against J558 allografts was statistically significant, but effectively limited, in the immune-deficient model.

We next performed in vivo antitumor efficacy studies using single-agent TAK-931 in J558 allografts of the immuno-competent syngeneic model. TAK-931 was orally administered to J558-allografted BALB/c mice (*n* = 10) at 60 mg/kg, once a day, for 21 days, and tumor volumes were monitored for 49 days or until the humane endpoint (Fig. 7e–h). The preclinical Kaplan–Meier survival analysis was also conducted with an endpoint tumor volume of 1200 mm^3^ as a surrogate for mortality (Fig. 7i–k and Fig. S15d). In the vehicle control of 0.5% methylcellulose (*n* = 10), all 10 mice reached the humane endpoint within 14 days. In TAK-931 treatment, in contrast, no mouse reached the endpoint within 14 days of the treatment, and growth rate inhibition (GRI) was 48% on Day 10 (*p* < 0.01), demonstrating the significant antiproliferative activity of TAK-931 in the immuno-competent syngeneic model (Fig. 7f). Additionally, 4 of 10 mice in TAK-931 treatment group were complete responders (CRs). No severe body weight loss (BWL) was observed; the maximal mean %BWL was 0.6% on Day 3 (Fig. S15e–g). The preclinical Kaplan–Meier survival analysis also revealed significant survival benefits of TAK-931 treatment (Fig. 7f, i).

To evaluate tumor rejection effects of TAK-931-induced antitumor efficacy, we conducted tumor-rechallenge studies using four CRs of the single-agent TAK-931 efficacy studies (TAK-931-CRs). Five naive female BALB/c mice (naive control) and four TAK-931-CRs were inoculated subcutaneously with 1.0 × 10^5^ J558 cells in the lower left flank, and the tumor volumes were monitored until the humane endpoint. In the naive controls, all the inoculated J558 allografts grew to reach the endpoint on Day 20 or earlier (5/5) (Fig. 7g, black lines). In contrast, the TAK-931-CRs (4/4) exhibited tumor-free survival (TFS) for 46 days, when the study ended (Fig. 7g, red lines). These results indicate that the TAK-931-CRs acquired tumor rejection against J558 cells. The preclinical Kaplan–Meier survival analysis also confirmed the significant survival benefit against J558 allografts in the TAK-931-CRs (Fig. 7j, l).

To confirm the on-target immunogenicity against J558 cells, we subsequently conducted a second rechallenge study by inoculating another cell line, CT26. Five naive control mice and four TFS mice of the first rechallenge were inoculated subcutaneously with 1.0 × 10^5^ CT26 cells in the left upper flank, and the tumor volumes were monitored until the humane endpoint was reached (Fig. 7h, k, l). All naive controls reached the humane endpoint on Day 23 or earlier: one on Day 20 and four on Day 23 (Fig. 7h, black lines). The first-rechallenge CRs also reached a humane endpoint: two on Day 23, one on Day 26, and one on Day 33 (Fig. 7h, red lines). In the preclinical Kaplan–Meier survival analysis, no significant survival benefit in the second rechallenge was observed (Fig. 7k, l). Although the sample size was limited, the anti-m-CD8 antibodies remarkably attenuated immunogenicity against the J558 cells (Fig. 7m).

### Combination TAK-931 and ICI treatment enhances antitumor efficacy in vivo

**Fig. 8:**
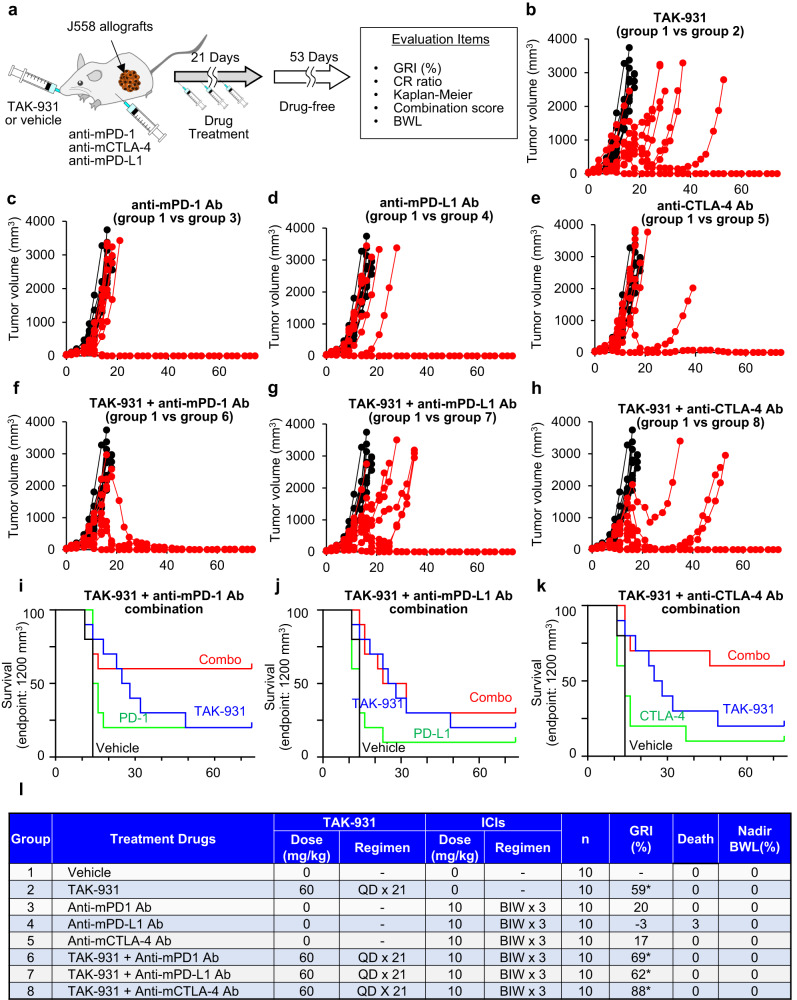
Antitumor efficacy of combination TAK-931 and ICI treatment in the J558 mouse allograft model. **a** Experimental schemes of antitumor efficacy studies with combination TAK-931 and ICI treatment in J558 mouse syngeneic model. **b**–**e** Antitumor efficacy of single-agent TAK-931 or ICI treatment in J558-allograft model. The J558-allograft mice were orally administered with TAK-931, Vehicle: CR = 0/10, TAK-931: CR = 2/10 **b** or intraperitoneally with m-PD-1, Vehicle: CR = 0/10, TAK-931: CR = 2/10 **c**, m-PD-L1, Vehicle: CR = 0/10, TAK-931: CR = 1/10 **d**, m-CTLA-4, Vehicle: CR = 0/10, TAK-931: CR = 1/10 **e** at the indicated regimen. Black and red indicate vehicle and the indicated drug treatments, respectively. Data are shown as tumor volumes (mm^3^); *n* = 10. **f**–**h** Antitumor efficacy of combination treatment with TAK-931 and ICIs in J558-allograft model. The J558-allograft mice were administered with m-PD-1, Vehicle: CR = 0/10, TAK-931: CR = 6/10 **f**, m-PD-L1, Vehicle: CR = 0/10, TAK-931: CR = 3/10 **g**, or m-CTLA-4, Vehicle: CR = 0/10, TAK-931: CR = 7/10 **h** in combination with TAK-931 at the indicated regimen. Black and red indicate vehicle and the indicated drug treatments, respectively. The efficacy data are plotted as tumor volumes (mm^3^); *n* = 10. **i**–**k** Preclinical Kaplan–Meier survival curves at 1200 mm^3^ of the endpoint tumor volume. Antitumor efficacy results of m-PD-1 and TAK-931 **i**, m-PD-L1 and TAK-931 **j**, and m-CTLA-4 and TAK-931 **k** in the J558-allograft model are shown. **l** Summary of the antitumor efficacy studies in combination treatment in J558-allograft model. Source data are provided as a Source Data file.

Finally, we conducted in vivo antitumor efficacy studies for the combination of TAK-931 with ICIs, which were anti-mPD-1, anti-mPD-L1, and anti-mCTLA-4 antibodies, in the immune-competent J558-allografted BALB/c mouse model at the indicated dosing regimens (Fig. 8a). The GRIs in single-agent treatments on Day 74 were 0% (CR = 0) in vehicle control (Fig. 8b–l, group 1 [black lines]), 59% (*p* < 0.001, CR = 2) in TAK-931 (Fig. 8b, group 2 [red lines]), 20% (*p* = 0.143, CR = 2) in anti-mPD-1 (Fig. 8c, group 3 [red lines]), −3% (*p* = 0.779, CR = 1) in anti-mPD-L1 (Fig. 8d, group 4 [red lines]), and 17% (*p* = 0.226, CR = 1) in anti-mCTLA-4 (Fig. 8e, group 5 [red lines]). Significant antiproliferative activity on GRI was observed in the TAK-931 treatment but not in single-agent ICI treatments. Combination ICIs and TAK-931 treatment, however, enhanced antiproliferative activity (Fig. 8f–l): GRIs were 69% (*p* < 0.001, CR = 6) in the anti-mPD-1 and TAK-931 combination (Fig. 8f, group 6 [red lines]), 62% (*p* < 0.001, CR = 3) in the anti-mPD-L1 and TAK-931 combination (Fig. 8g, group 7 [red lines]), and 88% (*p* < 0.001, CR = 7) in the anti-mCTLA-4 and TAK-931 combination (Fig. 8h, group 8 [red lines]). No significant BWL was observed in any treatment group (Fig. S16a–c). Statistical analyses of the synergy scores also revealed an additive effect for each combination. The combination scores for synergy analysis during Days 0–74 were 10, −5, and −13 in the anti-mPD-1 and TAK-931, anti-mPD-L1 and TAK-931, and anti-mCTLA-4 and TAK-931 combinations, respectively. The preclinical Kaplan–Meier survival of 1200 mm^3^ tumor volume endpoint also demonstrated the combinational survival benefit compared to the benefit of each single-agent treatment, while the effects from combination with anti-mPD-L1 appeared to be relatively moderate (Fig. 8i–l). These findings indicate that TAK-931-induced RS immunologically activates the TME, wherein the activated T cells are more accumulated, leading to enhanced antitumor activity of ICIs.

## Discussion

RS is a central pillar of cancer hallmarks, broadly and intensively affecting various oncogenic signaling pathways^1,2^. The CDC7-specific inhibitor, TAK-931, was successfully developed as a next-generation RS-inducer for cancer drug candidates^14–17^. In this study, using multilayer-omics analyses for in vitro and in vivo models, we preclinically investigated the novel aspects of the CDC7 inhibitor TAK-931 on RS-mediated antitumor efficacy and immunity to evaluate the therapeutic potential of TAK-931 for combination treatment with ICIs.

With emerging ICIs as game-changers, cancer therapeutics have drastically improved in the past decade to exhibit great therapeutic advances in a subset of inflamed tumors (hot tumors)^20^. ICIs are currently used as a standard of care in a broad range of cancer types, including melanoma, non-small cell lung cancer, renal cell carcinoma, urothelial carcinoma, head and neck squamous cell carcinoma (HNSCC), Merkel cell carcinoma, gastric carcinoma, hepatocellular carcinoma, Hodgkin’s lymphoma, and microsatellite instability (MSI)-positive tumors^20–23,38^. However, non-inflamed tumors (cold tumors), which constitute a large proportion of all tumors, are still refractory or poorly responsive to ICIs^24^. To improve therapeutic responses in cold tumors, various combination therapies with ICIs and small-molecule compounds are being explored, such as sting agonists^39–41^, BTK inhibitors^42^, PI3K inhibitors^43^, MPS1/TTK inhibitors^44^, CDK4/6 inhibitors^45,46^, and CDK7 inhibitors^47^. Notably, RS inducers, such as PARP inhibitors and ATR inhibitors, have recently attracted attention as combination partners for ICIs in both preclinical and clinical studies^13,48–50^. Based on accumulating evidence, RS inducers are expected to immunologically activate TME through enhanced genomic instability in cancer and consequent innate immune pathway activation. Currently, clinical potencies of these molecules in combination with ICIs are being evaluated in multiple lines of clinical trials^13,38,51^.

Accumulating evidence in preclinical studies also suggests the therapeutic potential of the CDC7 inhibitors, which are likely to be more specific as they directly target the replication fork, in RS-mediated antitumor immunity^14,18,52,53^. In this study, we demonstrated that TAK-931-induced RS generated the inflamed senescent aneuploid cells, which most intensively upregulated the inflammatory cytokine-related and chemokine-related pathways both in vitro and in vivo. The scRNA-seq analyses revealed that inflammatory-high segmentation in the TAK-931-treated cells significantly upregulated the aneuploidy-related stress hallmarks^33^ (Fig. 4). Reversely, InferCNV analyses of scRNA-seq also demonstrated that the inflammatory-related hallmarks were significantly enriched in the CNV-high segmentation (Fig. 4); thus, advanced aneuploidy appears to be a key biological event in RS-induced inflammatory activation in TAK-931 treatment. Supporting the mechanisms of TAK-931 in inflammatory activation in vitro, the ability of TAK-931 to impact tumor infiltration of immune cells, antitumor immunity, and antitumor efficacy were preclinically determined in the multilayer-omics analyses of the mouse syngeneic allograft model. Accordingly, another CDC7 inhibitor, XL413, has also been reported to promote tumor infiltration of immune cells in genetically engineered mouse small-cell lung cancer models^18^. Furthermore, we preclinically validated that combination TAK-931 and ICI treatments significantly improved antitumor activities in the mouse allograft models. Based on a series of these preclinical pharmacology studies, the therapeutic potential of the CDC7 inhibitors is greatly expected to be of improved benefit relative to that of currently used immunotherapies.

Although the therapeutic potential of the CDC7 inhibitor was preclinically validated in this study, the detailed molecular mechanisms of how TAK-931-induced RS and its consequent aneuploidy elevate inflammatory cytokine and chemokine pathways remain to be elucidated. The cGAS–STING pathway would be the first candidate to explain the functional interactions of TAK-931-induced RS and/or CIN with inflammatory signaling, since the cGAS-STING pathway plays an important role in the cytosolic DNA-sensing machinery to activate the innate immune pathway^54–56^. Once cytosolic DNAs are detected by cGAS, STING activates TBK1 to promote the IRF3 and NFkB signaling pathways, leading to inflammatory pathway activation. The TAK-931-treated inflamed aneuploid cells, however, did not significantly increase phospho-IRF3, phospho-TBK1, or cGAS nuclear accumulation (Fig. S3a, b). TAK-931 treatment equivalently elevated NF_k_B reporter activity between STING KO, negative KO, and no CRISPR (Fig. S3c, d). Moreover, in the case of RS-induced by a CDK7 inhibitor (YKL-5-124), which suppresses a CDC7 downstream substrate MCM2 to generate RS, the immunostimulatory effects caused by the CDK inhibitor have also been reported to be independent of the cGAS-STING cytosolic DNA-sensing pathway^47^. Although the molecular mechanisms are still unclear, CDC7-MCM2 pathway-mediated RS may exert inflammatory activation independently of the canonical cGAS-STING cytosolic DNA-sensing pathway. Further mechanistic analyses need to be conducted to clarify the functional interaction between CDC7 inhibition-induced RS and inflammatory activation.

In conclusion, we demonstrated that TAK-931-induced RS generates inflamed aneuploid cells that highly express inflammatory cytokines and chemokines. Our preclinical models validate that TAK-931 promotes tumor infiltration of immune cells, antitumor immunity, and antitumor efficacy. The combinatorial treatment of TAK-931 with ICIs significantly improved antitumor activities in the preclinical models. These findings expand the therapeutic potential of TAK-931, supporting the exploration of its clinical benefit in combination with current immunotherapies.

## Methods

### Ethical statement

The protocol and any amendments or procedures involving the care and use of animals in this study were reviewed and approved by the IACUC of Crown Bioscience Inc. and National Cancer Center Japan (approval number: K21-015) before study initiation. The care and use of animals were conducted in accordance with the regulations of the Association for Assessment and Accreditation of Laboratory Animal Care (AAALAC). Female BALB/c mice and female BALB/c nude mice were used. Tumor volumes and body weights were regularly measured three times a week according to the study protocol.

### The following criteria (humane endpoints) are used to determine if the animals are to be removed from study and euthanized

Weight loss—the body weight of all animals will be monitored throughout the study and animals will be euthanized if they loss 20% of their body weight relative to the start of the study, or if the animals loss 15% of their body weight in any 24-hour time frame.

Tumor size—if the tumor volume reaches greater than 10% of animals body weight or tumor length reaches 2 cm, have an ulcerated appearance, interfere with eating drinking, urinating, defecating or walking.

### Paralysis–loss of function in either forelimbs or hind limbs

Tumor appearance—necrosis and ulceration can be a function of either or both the growth rate and the treatment. The appearance of large or open ulceration in the xenograft will result in euthanasia for the animal.

Animal appearance/behavior—criteria that will initiate euthanasia if not responsive to palliative treatment include: dehydration, hypothermia, abnormal breathing, low activity level, obvious pain, general poor body condition, diarrhea, and skin lesions.

### Compounds

TAK-931 was synthesized by Takeda Pharmaceutical Company Ltd^14,16^. Olaparib, VE-821, BMS-265246, Alisertib, BAY-1217389, and XL413 were purchased from Selleck Chemicals (Houston, USA). AZD-1152 was purchased from Sigma-Aldrich.

### Cell lines

HeLa and A549 cells were purchased from RIKEN BRC. COLO205, J558, and CT26 cells were purchased from ATCC. PBMC was purchased from COSMO BIO CO., LTD. A549-Dual Cells were purchased from InvivoGen.

### Dual reporter assay

A549-Dual cells (InvivoGen, #a549d-nfis) were seeded in 96-well plates at a density of 10,000 cells per well. Cells were stimulated for 72 h with compound. The supernatant was then subjected to a colorimetric enzyme assay to measure alkaline phosphatase (AP) activity using the QUANTI-Blue solution (InvivoGen, #rep-qbs). The supernatant was then incubated at 37 °C for 2 h, and the optical density was read at 650 nm in SpectraMax Paradigm (Molecular devices). Luciferase activity was measured using the QUANTI-Luc (InvivoGen, #rep-qlc). The light signal produced was then quantified using SpectraMax Paradigm (Molecular devices).

### Cell growth assays

The cell growth assays were performed as described previously^25^. Cell growth was evaluated by intracellular ATP concentrations using the CellTiter-Glo® 2.0 luminescent cell viability assay (Promega Corp., Madison, WI, USA). The luminescence was measured using SpectraMax Paradigm (Molecular Devices, LLC., San Jose, CA, USA). The combination effect was calculated by SynergyFinder (https://synergyfinder.org).

### Immunofluorescence assay

The immunofluorescence assay was performed as described previously^25,57^. HeLa and A549 cells were fixed for 15 min with 4% paraformaldehyde in phosphate-buffered saline (PBS), followed by permeabilization for 15 min with a Triton X-100-containing buffer. Anti-α tubulin (DM1A) (T9026; Sigma-Aldrich), anti-cGAS (D1D3G) (# 15102; Cell Signaling Technology), anti-Lamin B (C-20) (sc-6216; Santa Cruz Biotechnology) antibodies were used at concentrations of 1–2 μg/mL for the immunofluorescence assays. Images were captured with an Axio Vert.A1 microscope (Carl Zeiss) fitted with an EC Plan-Neofluar ×20 lens and BZ-810 (KEYENCE).

### Immunoblotting

Immunoblotting was performed as described previously^25^. The following antibodies were used at concentrations of 0.1–0.5 μg/mL: anti-pMCM2 (EPR4170(2)) (ab133243; Abcam), anti-MCM2 (E-8) (sc-373702; Santa Cruz Biotechnology), anti-pTBK1 (D52C2) (# 5483; Cell Signaling Technology), anti-TBK1 (D1B4) (# 3504; Cell Signaling Technology), anti-pSTING (D8K6H) (# 40818; Cell Signaling Technology), anti-STING (D2P2F) (# 13647; Cell Signaling Technology), anti-pIRF3 (4D4G) (#4947; Cell Signaling Technology), anti-IRF3 (D83B9) (# 4302; Cell Signaling Technology), anti-BRCA2 (D9S6V) (#10741; Cell Signaling Technology), and anti-GAPDH (14C10) (# 2118; Cell Signaling Technology). Immunoblotted proteins were visualized by chemiluminescence.

### RNA preparation and TaqMan quantitative reverse transcription–PCR analysis

Total RNA was extracted using the RNeasy Miniprep kit (Qiagen). cDNAs were synthesized from 2.5 mg of the total RNA using the SuperScript™ VILO™ Master Mix (Thermo Fisher Scientific, Waltham, MA, USA). Real-time PCR was performed using an Applied Biosystems 7500 Fast Real-Time PCR System according to the manufacturer’s protocol (Applied Biosystems). The 6-carboxyfluorescein (FAM) fluorescence released from each sample was measured as a function of the PCR cycle number (Ct) using the Applied Biosystems 7500 Fast Real-Time PCR System. Gene expression was calculated using the comparative Ct method. The expression ratios of the indicated genes were quantified using the GAPDH expression in each cell line. The TaqMan probe product IDs were human GAPDH (Hs99999905_m1), human IL6 (Hs00174131_m1), human CCL5 (Hs00982282_m1), human CXCL10 (Hs00171042_m1), mouse GAPDH (Mm99999915_g1), mouse CXCL10 (Mm00445235_m1), and mouse IL1A (Mm00439620_m1).

### SA-βGAL activity assay

The indicated cell lines were treated with 300 nM TAK-931 for 72 h and fixed with a fixative solution (Cat#11674, Cell Signaling Technology). Cytochemical staining for SA-β-galactosidase was performed using a Senescence β-Galactosidase Staining Kit (Cat#9860, Cell Signaling Technology) at pH 6.0. Images were captured with a BIOREVO BZ-9000 microscope (KEYENCE, Osaka, Japan).

### FACS analysis

The cells were fixed with 70% ethanol. After washing with PBS containing 4% fetal bovine serum, the cells were incubated in PBS containing 4% fetal bovine serum, 20 μg/mL propidium iodide (Thermo Fisher Scientific), and 100 μg/mL RNase A (NIPPON GENE). Ten thousand cells were analyzed using the FACSCanto™ II flow cytometer (Becton-Dickinson, Franklin Lakes, NJ, USA).

### Phospho-Histone H3 staining

The cells were fixed with 70% ethanol. After washing with PBS containing 4% fetal bovine serum, the cells were incubated in PBS containing 0.25% Triton at 4  °C for 15 min. Cells were then washed in PBS containing 4% fetal bovine serum and resuspend in 100 μL of Phospho-Histone H3 (Ser 10) (D2C8) (Alexa Fluor® 488 Conjugate) antibody (#3465; Cell Signaling Technology, 1:50 dilution) for 20 min at room temperature. Cells were then washed with PBS containing 4% fetal bovine serum and resuspend in PBS containing 4% fetal bovine serum 20 μg/mL propidium iodide (Thermo Fisher Scientific), and 100 μg/mL RNase A (NIPPON GENE). Cells were analyzed using the FACSCanto™ II flow cytometer (Becton-Dickinson, Franklin Lakes, NJ, USA). The data was analyzed using FlowJo software.

### siRNA knockdown experiment

HeLa cells split into 6-well plates were cultured in a medium without antibiotics. The cells were transfected with 25 pmol siRNA (Negative Control siRNA, #, 1022076 Qiagen, siGENOME Human BRCA siRNA, SMARTPool, # M-003462-01-0005, Horizon) using Lipofectamine RNAiMAX Reagent (Invitrogen). At 6 hours after transfection, the culture medium was replaced with fresh medium without antibiotics, and the cells were subsequently cultured for 72 hours.

### Statistics and reproducibility

Immunohistochemistry images in vivo in Fig. 5k are a representative example of four individual J558-allograft tumors from one experiment. Immunohistochemistry images in vivo in Fig. 7b are a representative example of eight individual J558-allograft tumors from one experiment. All bright-field images of microscopy show typical data reproduced in multiple experiments. Parametric statistical analysis by Student’s *t* test was used for the comparison of the in vitro antiproliferation and in vivo antitumor efficacy data, if both normal distribution and variance equality of the sample data were verified by Shapiro–Wilk normality and Gaussian distribution tests, respectively. If either normal distribution or variance equality was not determined, non-parametric statistical analysis by the Wilcoxon–Mann–Whitney test was used. Statistical analysis was performed using GraphPad Prism (GraphPad Software, Inc., San Diego, CA, USA), Excel (Microsoft) software, or R version 3.3.0, and differences were considered significant at *p* < 0.05.

### Network analysis for bulk RNA sequencing

Bioinformatics analysis for upregulated genes identified in the transcriptome analysis was performed as described previously^15^. In brief, the 139 48-h upregulated genes and 504 72-h upregulated genes were submitted to Metascape (metascape.org.) to identify the statistically enriched terms. The terms with the best *p*-values within each cluster were selected as representative terms and displayed in a dendrogram. The heatmap cells were colored according to their *p*-values: white cells indicate the lack of enrichment for that term. A subset of representative terms from the complete cluster was selected to convert them into a network layout. More specifically, each term was represented by a circular node, the size of which is proportional to the number of input genes falling into that term and the color of which represents its cluster identity (i.e., nodes of the same color belong to the same cluster). Terms with a similarity score of >0.3 are linked by an edge (the thickness of the edge represents the similarity score). The network was visualized using Cytoscape (v3.1.2) with force-directed layout and edge bundled for clarity. One term from each cluster was selected, and its term description was shown as a label. The same enrichment network had its nodes colored according to *p*-value: the darker the color, the more statistically significant the node is (see legend for *p* value ranges). The nodes for the same enrichment network were displayed as pies. Each pie sector is proportional to the number of hits originated from a gene list.

Subextractor network analysis with the 48-h and 72-h upregulated genes was performed. The networks of the 139 48-h upregulated genes and 504 72-h upregulated genes were visualized using Cytoscape (v3.1.2). Each term is represented by a circular node, the size of which is proportional to the number of input genes falling into that term and the color of which represents its cluster identity (i.e., nodes of the same color belong to the same cluster). Terms with a similarity score of >0.3 are linked by an edge (the thickness of the edge represents the similarity score). One term from each cluster was selected to have its term description shown as a label. The cross-network of the merged 48-h and 72-h upregulated genes was visualized using Cytoscape (v3.1.2).

### scRNA-seq experiment procedure

scRNA-seq was performed as described previously^58^. Briefly, DMSO- or TAK-931-treated HeLa cells (in vitro samples), and vehicle- or TAK-931-treated J558 allografts (in vivo samples) were processed using the Chromium Single Cell 3′ Solution (v3.1 Chemistry; 10× Genomics, Pleasanton, CA, USA) per the manufacturer’s recommendations. The cells were resuspended at 1 × 10^6^ cells per mL. To generate gel bead-in emulsions (GEMs), the master mix was mixed with the cell suspension, and gel beads and partitioning oils were loaded on a Chromium chip. Next, GEM-reverse transcription (RT) reaction, cDNA amplification, and gene expression library generation were performed using Chromium kits and reagents. QC of constructed libraries was conducted with Agilent Bioanalyzer (Agilent technologies).

### scRNA-seq data processing with Seurat

After library generation, sequencing was performed using a NovaSeq 6000 (Illumina Inc.). The fastq files were generated from the bcl files in Cell Ranger (version 6.0, 10× Genomics). The sequence reads were aligned to UCSC hg38, and Unique Molecular Identifiers were counted for each gene in each cell barcode using Cell Ranger count. The data were then processed by R package Seurat using Cell Ranger output files, and barcodes.tsv, genes.tsv, and matrix.mtx (Seurat version 4.0)(10.1016/j.cell.2021.04.048). In HeLa cell study, cells were filtered based on unique feature counts (*nFeature_RNA* > *5000*) and mitochondrial counts (*percent.mt* < *10*) for quality control. In mouse model scRNA-seq analysis, cells were filtered based on unique feature counts (*nFeature_RNA* > *2000*) and mitochondrial counts (*percent.mt* < *15*) for quality control.

### GSEA analysis using scRNA-seq dataset

To calculate gene set enrichment score, ssGSEA analysis were performed using R packages (R package “escape”, version 1.0.0^59^) (R package “dittoSeq”, version 1.2.5^60^). Gene set c “Human MSigDB Collections H:hallmark” was selected for analysis.

### scRNA-seq-based copy number variation analyses

R package InferCNV (https://github.com/broadinstitute/inferCNV) was used to predict somatic large-scale CNV (gains or deletions of entire chromosomes or large segments of chromosomes) for scRNA-seq data in HeLa cells treated with DMSO or TAK-931(R version 4.1.0 “Camp Pontanezen”, InferCNV version 1.9.1)^34,35^. Analyses were performed according to the instruction shown in inferCNV wiki “Using 10x data”. First, raw count matrix of single-cell RNA-seq gene expression was extracted from Seurat object. In addition to the raw counts matrix, annotation file and gene/chromosome position files are prepared as input. Using those inputs, object was created using command “CreateInfercnvObject”(parameters were default). CNV scores were calculated using “infercnv::run” (cutoff score was 0.1, other parameters were default). Scores are returned to Seurat object and following analysis were conducted. A six-state model for HMM-based-CNV prediction, i6 HMM, (https://github.com/broadinstitute/infercnv/wiki/inferCNV-HMM-based-CNV-Prediction-Methods) was used to predicts the CNV levels at the chromosome regions.

### PBMC migration assay

The PBMC migration assay was performed using a ThinCert™ 24-well cell-culture insert with a 3.0-μm pore membrane (Cat#662631, Greiner Bio-One, Germany)^61^. PBMCs at 3.0 × 10^5^/well were placed in the upper chambers, and the conditioned medium from DMSO- or TAK-931-treated A549 cells was used to fill in the lower chambers. After 3 h incubation, the number of membrane-through migratory PBMCs in the lower chamber was counted microscopically in five different fields.

### Immune panel studies

Immune panel studies were performed at Shanghai Medicilon Inc (Shanghai, China). The J558-allografted tumor samples (40–1000 mg tumor mass) were sliced into small pieces (2–4 mm^3^) and then isolated into single cells using a gentle MACS Dissociator (Miltenyi Biotec, Bergisch Gladbach, Germany) at 37 °C for 40 min using the mouse tumor dissociation kit (Miltenyi Biotech, 130-096-730). The digested tumors were filtered through a 70-μm strainer to remove cell aggregates. The cells were resuspended with 30% Percoll and were carefully layered on 70% Percoll. The cell suspension was centrifuged at 400 × *g* for 30 min at room temperature, and then the cell layer between 30% and 70% Percoll was harvested. After being washed with Dulbecco’s PBS, the cells were resuspended in BD staining buffer, and the cell numbers were counted on a counting plate under a microscope. The isolated cells were aliquoted into a 96-well V-bottom plate, and after the indicated antibody mixture was added, the samples were incubated for 30–60 min at 4 °C in the dark. After fixation with BD fixation buffer at 4 °C for 30 min in the dark, the samples were resuspended in 200–400 μL of staining buffer and incubated at 4 °C for 0–24 h in the dark until the analyses with BD FACSCelesta. The antibodies used are listed in the Source Data file.

### Immunohistochemistry

Samples were formalin-fixed and embedded in paraffin, subjected to hematoxylin and eosin (HE) staining or immunohistochemistry (performed by Morphotechnology, Co. Ltd., Hokkaido, Japan) using primary antibodies against anti-CD3 (SP7) (GeneTex, #GTX16669), anti-CD4 (D7D2Z) (#25229, Cell Signaling Technology), anti-CD8 (D4W2Z) (#98941, Cell Signaling Technology), anti-CD11c (N418) (GeneTex, #GTX74940), anti-pMCM2 (EPR4170(2)) (Abcam, #ab133243), and anti-γH2AX (Ser139) (20E3) (#9718, Cell Signaling Technology). Immunohistochemical analysis of PD-1 was performed using formalin-fixed, paraffin-embedded tumor samples with anti-PD-1 (D7D5W) (#84651, Cell Signaling Technology) by GenoStaff, Co. Ltd., (Tokyo, Japan).

### Antitumor efficacy studies of combination TAK-931 and ICI treatment in the J558 syngeneic mouse model

Antitumor efficacy studies for TAK-931 in combination with ICIs were performed in the J558 syngeneic mouse model at Shanghai Medicilon Inc. The female BALB/c mice were inoculated subcutaneously with 1 × 10^5^ J558 cells each (in 0.1 mL, no Matrigel) in the right flank. Tumor growth was monitored with vernier calipers, and the mean tumor volume (MTV) was calculated using the formula (0.5 × [length × width^2^]). When the MTV reached approximately 30 mm^3^, the animals were randomized into the indicated treatment groups (*n* = 10/group) on Day 0 of the study, and dosing was initiated on the same day. The antibodies used are listed in the Source Data file. Tumor size and body weight were measured three times weekly until the humane endpoint according to Takeda’s Institutional Animal Care and Use Committee (IACUC) guideline, and the study was completed following the last measurement on Day 74. The mean BWL was determined for each group using the mean body weight data from the treatment period (versus pre-dose body weights on Day 0), and the maximal mean percent BWL (%BWL) was also calculated based on pre-dose body weights. Inhibition of tumor growth was determined by calculating the percent GRI on Day 16 using the following formula:

GRI (%) = 100% × (mean growth rate of control – mean growth rate of treatment)/(mean growth rate of vehicle).

The Kaplan–Meier survival analysis was performed using an endpoint tumor volume of 1200 mm^3^ as a surrogate for mortality.

The differences in the tumor growth trends over time between pairs of treatment groups were assessed by fitting each animal’s data to a simple exponential growth model and comparing the mean growth rates of the two groups. The difference in the growth rates was summarized by the GRI, which is the reduction in the growth rate experienced by the treatment group relative to that of the reference group, expressed as a fraction of the vehicle growth rate. A positive GRI indicates that the tumors in the treatment group grew at a reduced rate relative to that of the reference group. A statistically significant *p*-value suggested that the trends over time for the two treatment groups were different.

### Combination analysis

Combination analysis was performed to determine the combinational drug treatment benefit, which was based on the estimated tumor growth. The statistically significant negative synergy scores indicate a synergistic combination. The statistically significant positive synergy scores indicate a sub-additive combination when the combination performs better (i.e., has a lower growth rate) than the best-performing single agent. The statistically significant positive synergy scores indicate an antagonistic combination when the combination performs worse than the best-performing single agent. The scores that are not statistically significant should be considered additive.

The synergy score was calculated using the following formula:

$${{{{{\rm{Synergy\; score}}}}}}=(\mu {AB}-\mu A-\mu B+\mu {control})/\mu V\times 100\%$$

where *μ*_*AB*_, *μ*_*A*,_ *μ*_*B*,_ and *μ*_control_ are the mean growth rates for the combination, drug A, drug B, and control groups, respectively. *μ*_*V*_ is the mean tumor growth rate for the vehicle group, which in most cases is the same as the control group. The standard error of the synergy score was calculated as the squared standard error across the four groups. The degrees of freedom were estimated using the Welch–Satterthwaite equation. A hypothesis test was performed to determine if the synergy score differed from 0. The *p* values were calculated by dividing the synergy score by its standard error and tested against a *t*-distribution (two-tailed) with the above-calculated degrees of freedom.

### Tumor rechallenge of J558 syngeneic model in CR post-treatment with TAK-931 1)

#### 1) First rechallenge with J558 cells

naive female BALB/c mice and the CRs of female BALB/c mice previously treated with TAK-931 alone were inoculated subcutaneously with 1 × 10^5^ J558 cells each (in 0.1 mL, no Matrigel) in the left flank on Day 0. Four TAK-931-pretreated CR mice were rechallenged on Day 49 after the start of the treatment of the initial study and 29 days after the end of the treatment of the initial study, when no tumor was palpated (tumor volume = 0 mm^3^). For each of the selected mice, the tumor growth rates at both the primary inoculation site and the rechallenge site were monitored. Only the tumor growth at the rechallenge site in the left flank was reported in this study. The tumor growth was monitored with vernier calipers, and the MTV was calculated using the formula (0.5 × [length × width^2^]). The tumor size and body weight were measured three times weekly starting on Day 3 until the humane endpoint according to Takeda’s Institutional Animal Care and Use Committee (IACUC) guideline, and the study was terminated following the last measurement on Day 46 after rechallenge.

#### 2) Second rechallenge with CT26 WT cells

naive female BALB/c mice and female BALB/c mice with TFS previously rechallenged with J558 cells were inoculated subcutaneously with 2 × 10^5^ CT26 WT cells each (in 0.1 mL, no Matrigel) in the lower left flank area on Day 0. Four TFS mice were rechallenged on Day 46 after the previous rechallenge when the tumor growth of each mouse was in CR (tumor volume = 0 mm^3^). For each of the selected mice, the tumor growths at both the primary inoculation site and the rechallenge sites were monitored. Only the tumor growth at the second rechallenge site in the lower left flank was measured in this study. The tumor growth was monitored with vernier calipers, and the MTV was calculated using the formula (0.5 × [length × width^2^]). Tumor size and body weight were measured three times weekly starting on Day 2 until the humane endpoint according to Takeda’s Institutional Animal Care and Use Committee (IACUC) guideline, and the study was terminated following the last measurement on Day 33 after the rechallenge.

The protocol and any amendments or procedures involving the care and use of animals in this study were reviewed and approved by the IACUC of Crown Bioscience Inc. before study initiation. The care and use of animals were conducted in accordance with the regulations of the AAALAC.

### Antitumor efficacy studies of TAK-931 single-agent treatment in the J558 nude mouse model

Antitumor efficacy studies for TAK-931 were performed in the J558 nude mouse model at the National Cancer Center in Japan. The female BALB/c nude mice were inoculated subcutaneously with 1 × 10^5^ J558 cells each (in 0.1 mL, no Matrigel) in the right flank. Tumor growth was monitored with vernier calipers, and the mean tumor volume (MTV) was calculated using the formula (0.5 × [length × width^2^]). Tumor size and body weight were measured three times weekly until the humane endpoint according to the Institutional Animal Care and Use Committee (IACUC) guideline of the National Cancer Center Japan. The mean BWL was determined for each group using the mean body weight data from the treatment period (versus pre-dose body weights on Day 0). The Kaplan–Meier survival analysis was performed using an endpoint tumor volume of 1200 mm^3^ as a surrogate for mortality.

The protocol and any amendments or procedures involving the care and use of animals in this study were reviewed and approved by the National Cancer Center Japan before study initiation. The care and use of animals were conducted in accordance with the regulations of the Association for Assessment and Accreditation of Laboratory Animal Care (AAALAC).

### Reporting summary

Further information on research design is available in the Nature Portfolio Reporting Summary linked to this article.

### Supplementary information

Supplementary information

Reporting Summary

### Source data

Source Data

## Data Availability

The RNA-seq dataset of TAK-931 treated HeLa cells is available in the GEO database under accession number GSE245063. The single-cell RNA-seq dataset of TAK-931 treated HeLa cells is available in the GEO database under accession number GSE244987. The RNA-seq dataset of the J558-allograft mouse model treated with TAK-931 is available in the GEO database under accession number GSE244988. The Single-cell RNA-seq dataset of the J558-allograft mouse model treated with TAK-931 is available in the GEO database under accession number GSE244986. The remaining data are available within the Article, Supplementary Information, or Source Data file. Data and materials will be provided by Takeda pending scientific review and a completed material transfer agreement. Request for the data and materials should be submitted to A.O. as a point of contact. Source data are provided in this paper.

## Code availability

Main code sets are given in the Source Data file. Other statistical codes required to reanalyze the data are available upon request.

## References

[1] Dobbelstein M, Sorensen CS (2015). Exploiting replicative stress to treat cancer. Nat. Rev. Drug Discov..

[2] Zeman MK, Cimprich KA (2014). Causes and consequences of replication stress. Nat. Cell Biol..

[3] Kotsantis P (2016). Increased global transcription activity as a mechanism of replication stress in cancer. Nat. Commun..

[4] Gorgoulis VG (2005). Activation of the DNA damage checkpoint and genomic instability in human precancerous lesions. Nature.

[5] Halazonetis TD, Gorgoulis VG, Bartek J (2008). An oncogene-induced DNA damage model for cancer development. Science.

[6] Bartkova J (2005). DNA damage response as a candidate anti-cancer barrier in early human tumorigenesis. Nature.

[7] Masamsetti VP (2019). Replication stress induces mitotic death through parallel pathways regulated by WAPL and telomere deprotection. Nat. Commun..

[8] Lukas C (2011). 53BP1 nuclear bodies form around DNA lesions generated by mitotic transmission of chromosomes under replication stress. Nat. Cell Biol..

[9] Chan KL, Palmai-Pallag T, Ying S, Hickson ID (2009). Replication stress induces sister-chromatid bridging at fragile site loci in mitosis. Nat. Cell Biol..

[10] Hanahan D, Weinberg RA (2011). Hallmarks of cancer: the next generation. Cell.

[11] O’Connor MJ (2015). Targeting the DNA damage response in cancer. Mol. Cell.

[12] Gaillard H, Garcia-Muse T, Aguilera A (2015). Replication stress and cancer. Nat. Rev. Cancer.

[13] Pilger D, Seymour LW, Jackson SP (2021). Interfaces between cellular responses to DNA damage and cancer immunotherapy. Genes Dev..

[14] Iwai K (2019). Molecular mechanism and potential target indication of TAK-931, a novel CDC7-selective inhibitor. Sci. Adv..

[15] Iwai K (2021). A CDC7 inhibitor sensitizes DNA-damaging chemotherapies by suppressing homologous recombination repair to delay DNA damage recovery. Sci. Adv..

[16] Kurasawa O (2020). Discovery of a novel, highly potent, and selective thieno[3,2-d]pyrimidinone-based Cdc7 inhibitor with a quinuclidine moiety (TAK-931) as an orally active investigational antitumor agent. J. Med. Chem..

[17] Kuboki Y (2022). Safety, tolerability, and pharmacokinetics of TAK-931, a cell division cycle 7 inhibitor, in patients with advanced solid tumors: a phase I first-in-human study. Cancer Res. Commun..

[18] Wang C (2019). Inducing and exploiting vulnerabilities for the treatment of liver cancer. Nature.

[19] Faget DV, Ren Q, Stewart SA (2019). Unmasking senescence: context-dependent effects of SASP in cancer. Nat. Rev. Cancer.

[20] Sharma P, Allison JP (2015). The future of immune checkpoint therapy. Science.

[21] Topalian SL (2012). Safety, activity, and immune correlates of anti-PD-1 antibody in cancer. N. Engl. J. Med..

[22] Brahmer JR (2012). Safety and activity of anti-PD-L1 antibody in patients with advanced cancer. N. Engl. J. Med..

[23] Hodi FS (2010). Improved survival with ipilimumab in patients with metastatic melanoma. N. Engl. J. Med..

[24] Borcherding N (2018). Keeping tumors in check: a mechanistic review of clinical response and resistance to immune checkpoint blockade in cancer. J. Mol. Biol..

[25] Ohashi A (2015). Aneuploidy generates proteotoxic stress and DNA damage concurrently with p53-mediated post-mitotic apoptosis in SAC-impaired cells. Nat. Commun..

[26] Ewald JA, Desotelle JA, Wilding G, Jarrard DF (2010). Therapy-induced senescence in cancer. J. Natl. Cancer Inst..

[27] Misra RN (2003). 1H-Pyrazolo[3,4-b]pyridine inhibitors of cyclin-dependent kinases: highly potent 2,6-Difluorophenacyl analogues. Bioorg. Med. Chem. Lett..

[28] Pantelidou C (2019). PARP inhibitor efficacy depends on CD8( + ) T-cell recruitment via intratumoral STING pathway activation in BRCA-deficient models of triple-negative. Breast Cancer Cancer Discov..

[29] Bryant HE (2005). Specific killing of BRCA2-deficient tumours with inhibitors of poly(ADP-ribose) polymerase. Nature.

[30] Farmer H (2005). Targeting the DNA repair defect in BRCA mutant cells as a therapeutic strategy. Nature.

[31] Herranz N (2015). mTOR regulates MAPKAPK2 translation to control the senescence-associated secretory phenotype. Nat. Cell Biol..

[32] Garcia-Garcia C (2012). Dual mTORC1/2 and HER2 blockade results in antitumor activity in preclinical models of breast cancer resistant to anti-HER2 therapy. Clin. Cancer Res..

[33] Zhu J, Tsai HJ, Gordon MR, Li R (2018). Cellular stress associated with aneuploidy. Dev. Cell.

[34] Tirosh I (2016). Dissecting the multicellular ecosystem of metastatic melanoma by single-cell RNA-seq. Science.

[35] Patel AP (2014). Single-cell RNA-seq highlights intratumoral heterogeneity in primary glioblastoma. Science.

[36] Verkleij CPM (2020). Preclinical rationale for targeting the PD-1/PD-L1 axis in combination with a CD38 antibody in multiple myeloma and other CD38-positive malignancies. Cancers (Basel).

[37] Natalie A (2018). Antitumor activity associated with dual targeting of CD38 and programmed death-1 (PD-1) pathways in preclinical models. Cancer Res..

[38] Peyraud F, Italiano A (2020). Combined PARP inhibition and immune checkpoint therapy in solid tumors. Cancers (Basel).

[39] Ramanjulu JM (2018). Design of amidobenzimidazole STING receptor agonists with systemic activity. Nature.

[40] Pan BS (2020). An orally available non-nucleotide STING agonist with antitumor activity. Science.

[41] Chin EN (2020). Antitumor activity of a systemic STING-activating non-nucleotide cGAMP mimetic. Science.

[42] Jain, N. et al. A phase 2 study of nivolumab combined with ibrutinib in patients with diffuse large B-cell richter transformation of CLL. *Blood Adv*. 10.1182/bloodadvances.2022008790 (2022). 10.1182/bloodadvances.2022008790 PMC10189379 36287248

[43] Maharaj K (2020). The dual PI3Kdelta/CK1epsilon inhibitor umbralisib exhibits unique immunomodulatory effects on CLL T cells. Blood Adv..

[44] Kitajima S (2022). MPS1 inhibition primes immunogenicity of KRAS-LKB1 mutant lung cancer. Cancer Cell.

[45] Bai X (2023). CDK4/6 inhibition triggers ICAM1-driven immune response and sensitizes LKB1 mutant lung cancer to immunotherapy. Nat. Commun..

[46] Patterson-Fortin J (2023). Polymerase theta inhibition activates the cGAS-STING pathway and cooperates with immune checkpoint blockade in models of BRCA-deficient cancer. Nat. Commun..

[47] Zhang H (2020). CDK7 inhibition potentiates genome instability triggering anti-tumor immunity in small cell lung cancer. Cancer Cell.

[48] Ragu S, Matos-Rodrigues G, Lopez BS (2020). Replication stress, DNA damage, inflammatory cytokines and innate immune response. Genes (Basel).

[49] Hsieh RC (2022). ATR-mediated CD47 and PD-L1 up-regulation restricts radiotherapy-induced immune priming and abscopal responses in colorectal cancer. Sci. Immunol..

[50] Weng W (2023). Antibody-exatecan conjugates with a novel self-immolative moiety overcome resistance in colon and lung cancer. Cancer Discov..

[51] Pham MM, Ngoi NYL, Peng G, Tan DSP, Yap TA (2021). Development of poly(ADP-ribose) polymerase inhibitor and immunotherapy combinations: progress, pitfalls, and promises. Trends Cancer.

[52] Montagnoli A, Moll J, Colotta F (2010). Targeting cell division cycle 7 kinase: a new approach for cancer therapy. Clin. Cancer Res..

[53] Montagnoli A (2008). A Cdc7 kinase inhibitor restricts initiation of DNA replication and has antitumor activity. Nat. Chem. Biol..

[54] Mackenzie KJ (2017). cGAS surveillance of micronuclei links genome instability to innate immunity. Nature.

[55] Bakhoum SF, Cantley LC (2018). The multifaceted role of chromosomal instability in cancer and its microenvironment. Cell.

[56] Bakhoum SF (2018). Chromosomal instability drives metastasis through a cytosolic DNA response. Nature.

[57] Ohashi A, Ohori M, Iwai K (2016). Motor activity of centromere-associated protein-E contributes to its localization at the center of the midbody to regulate cytokinetic abscission. Oncotarget.

[58] Kashima Y (2021). Potentiality of multiple modalities for single-cell analyses to evaluate the tumor microenvironment in clinical specimens. Sci. Rep..

[59] Borcherding N (2021). Mapping the immune environment in clear cell renal carcinoma by single-cell genomics. Commun. Biol..

[60] Bunis DG, Andrews J, Fragiadakis GK, Burt TD, Sirota M (2020). dittoSeq: universal user-friendly single-cell and bulk RNA sequencing visualization toolkit. Bioinformatics.

[61] Zang YC (2000). Aberrant T cell migration toward RANTES and MIP-1 alpha in patients with multiple sclerosis. Overexpression of chemokine receptor CCR5. Brain.

