# Supplementary material for: CDC7 inhibition induces replication stress-mediated aneuploid cells with an inflammatory phenotype sensitizing tumors to immune checkpoint blockade
Source: Nat Commun. 2023 Nov 18;14:7490. doi: 10.1038/s41467-023-43274-3 (PMC10657413; doi:10.1038/s41467-023-43274-3)
Supplement: Supplementary file 1 — Supplementary information [file 41467_2023_43274_MOESM1_ESM.pdf]

Supplementary Information.

Title: CDC7 inhibition induces replication stress-mediated aneuploid cells with an inflammatory phenotype sensitizing tumors to immune checkpoint blockade

Authors: Tomoko Yamamori Morita[1]†, Jie Yu[2]†, Yukie Kashima[1,3]†, Ryo Kamata[1], Gaku Yamamoto[1], Tatsunori Minamide[1,4], Chiaki Mashima[1], Miyuki Yoshiya[1], Yuta Sakae[1], Toyohiro Yamauchi[1,5], Yumi Hakozaki[1], Shun-ichiro Kageyama[6], Akito Nakamura[2], Eric Lightcap[2], Kosuke Tanaka[1], Huifeng Niu[7], Karuppiah Kannan[8], and Akihiro Ohashi[1,2,5,]*

Inventory of Supplementary Figures**.**

Supplementary Figure. 1. (Related to Fig. 1)

Supplementary Figure. 2. (Related to Fig. 1)

Supplementary Figure. 3. (Related to Fig. 1)

Supplementary Figure. 4. (Related to Fig. 1)

Supplementary Figure. 5. (Related to Fig. 1)

Supplementary Figure. 6. (Related to Fig. 2)

Supplementary Figure. 7. (Related to Fig. 2)

Supplementary Figure. 8. (Related to Fig. 3)

Supplementary Figure. 9. (Related to Fig. 4)

Supplementary Figure. 10. (Related to Fig. 4)

Supplementary Figure. 11. (Related to Fig. 5)

Supplementary Figure. 12. (Related to Fig. 5)

Supplementary Figure. 13. (Related to Fig. 6)

Supplementary Figure. 14. (Related to Fig. 6)

Supplementary Figure. 15. (Related to Fig. 7)

Supplementary Figure. 16. (Related to Fig. 8)

Supplementary Table. 1. (Related to Fig. 5)

Supplementary Figure. 1

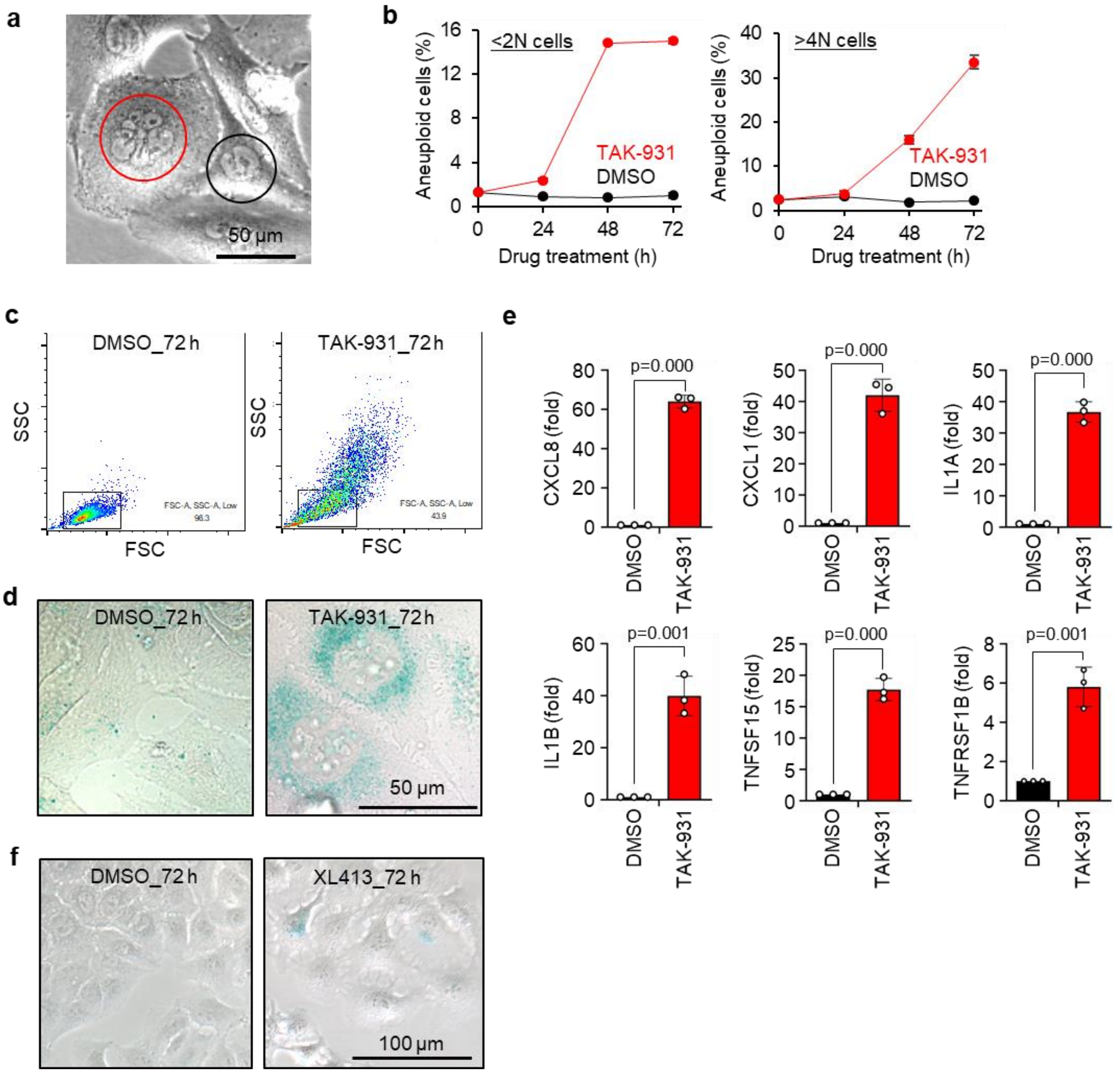

Supplementary Fig. 1. CDC7 inhibitor-mediated aneuploid cells exhibit senescence phenotype.

(a) Phase-contrast microscopy images of TAK-931-treated HeLa cells. Images were acquired 72 h after treatment with TAK-931 (300 nM). Red and black circles indicate grape-shaped multi-nucleoli and enlarged single nucleus, respectively. Black bar indicates 50 μm. (b) Time-dependent accumulation of aneuploid cells treated with TAK-931. The graph indicates the quantified cells of <2N (left) and 4N< (right) DNA contents. Black and red lines indicate DMSO and TAK-931 treatments, respectively. (c) Forward-scatter (FSC) and side-scatter (SSC) histograms in TAK-931-treated HeLa cells. HeLa cells were treated with DMSO (left) or TAK-931 (right) for 72 h. (d) Representative high-magnification images of SA-βGAL staining. HeLa cells were treated with DMSO (left) or TAK-931 (right) for 72 h. Black bars indicate 50 μm. (e) Quantitative reverse transcription–PCR (qRT-PCR) analysis of SASP genes in TAK-931-treated HeLa cells after 72 h of treatment. Data are presented as mean ± SD (n = 3). Two sided Student's t-test $p = 0.000$, $p = 0.000$, $p = 0.000$, $p = 0.001$, $p = 0.000$, $p = 0.0001$, respectively. (f) Representative images of SA-βGAL staining. A549 cells were treated with DMSO (left) or 10 μM XL413 (right) for 72 h. Black bars indicate 100 μm. Source data are provided as a Source Data file.

Supplementary Figure. 2

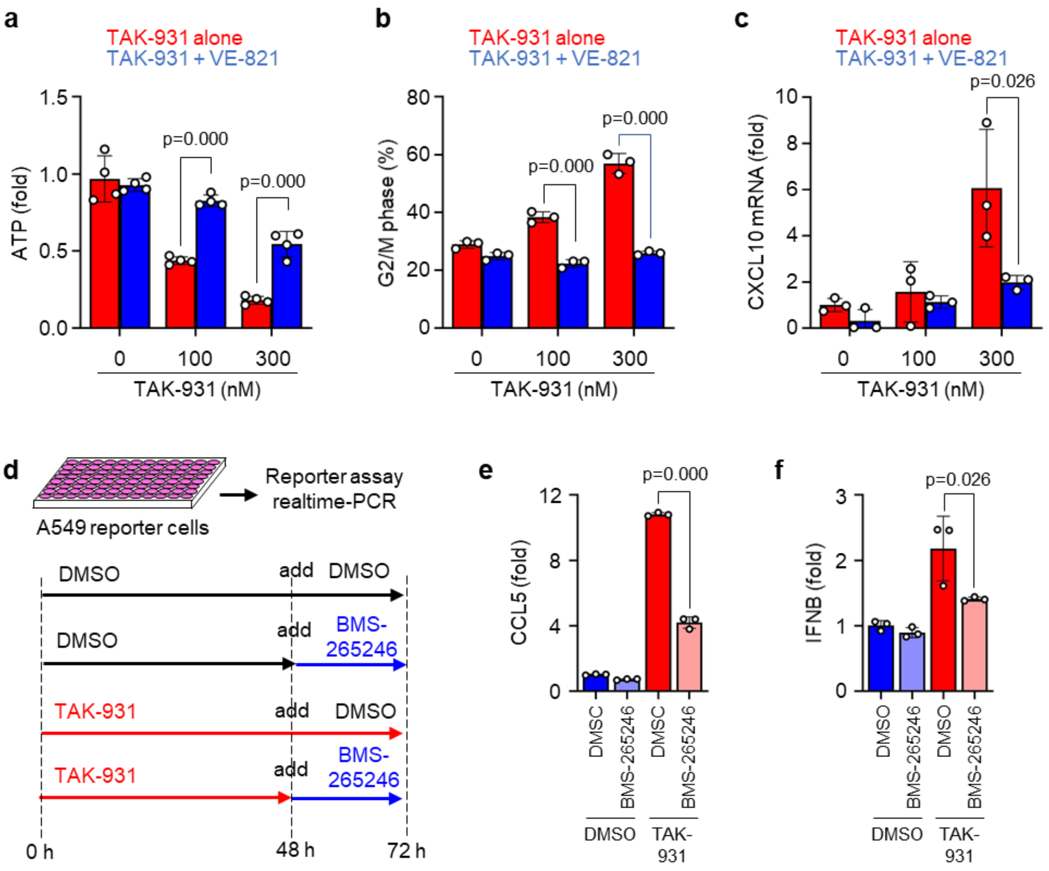

Supplementary Fig. 2. TAK-931-induced inflammatory activation is involved in ATR- and CDK1/2-mediated signaling pathways

(a) Growth assays of combination TAK-931 and VE-821 treatment. COLO205 cells were treated with TAK-931 alone (red) or TAK-931+VE-821 (1000 nM; blue) at the indicated concentrations for 72 h (mean ± SD [n = 4]). Two sided Student's t-test $p = 0.000$ (TAK-931 100 nM, 300 nM, respectively). (b) Cell cycle analysis in combination treatment with TAK-931 and VE-821. COLO205 cells were treated with DMSO, TAK-931 at 300 nM, VE-821 at 1000 nM, or the combination for 24 h. The graph indicates the quantified cells of 4N DNA contents. Data are presented as mean ± SD (n = 3). Two sided Student's t-test $p = 0.000$ (TAK-931 100 nM, 300 nM, respectively). (c) Quantitative reverse transcription–PCR analysis of CXCL10 in TAK-931-treated HeLa cells. HeLa cells were treated with DMSO or TAK-931 for 72 h. Data are presented as mean ± SD (n = 3). Two sided Student's t-test $p = 0.026$. (d) Experimental schemes of *in vitro* combination studies with TAK-931 and the CDK1/2 inhibitor BMS-265246. (e) (f) Quantitative reverse transcription–PCR analysis of CCL5 (e) and INFB (f) in TAK-931-treated A549-reporter cells. A549-reporter cells were treated with the indicated drugs as described in (d). Data are presented as mean ± SD (n = 3). One sided Student's t-test $p = 0.000$, $p = 0.026$, respectively. Source data are provided as a Source Data file.

Supplementary Figure. 3

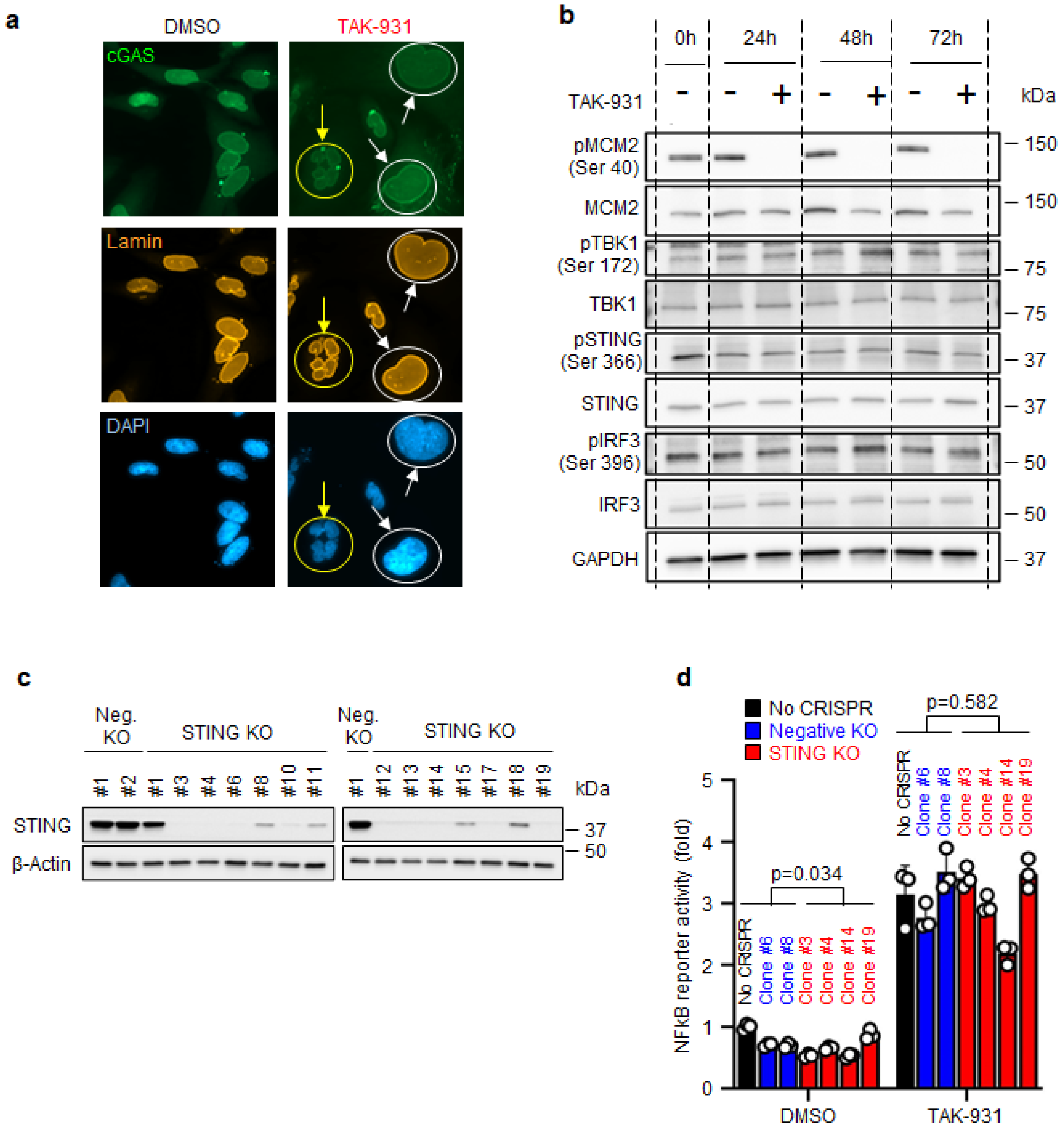

Supplementary Fig. 3. TAK-931-induced inflammatory activation may only limitedly involve the cGAS–STING pathway

(a) Effects of TAK-931 on cGAS nuclear localization. Green, orange, and blue signals indicate cGAS, Lamin, and DAPI (DNA), respectively. HeLa cells were treated with TAK-931 (300 nM) for 72 h. (b) Immunoblotting of cGAS-STING pathway-associated proteins in TAK-931-treated cells. HeLa cells were treated with DMSO or TAK-931 (300 nM) for the indicated periods. pMCM2, MCM2, pTBK1, TBK1, pSTING, STING, pIRF3, IRF3 were used as cGAS-STING pathway activating markers. GAPDH was used for loading controls. (c) Immunoblotting of STING in STING-kockout (KO) A549-reporter clones. Fourteen STING-KO and two negative control KO clones were subjected to STING expression screening. (d) NFkB reproter activity in STING-KO A549-reporter cells. The STING KO clones (red), negative KO closes (blue), and no-CRISPR cells (black) were treated with TAK-931 or DMSO for 72 h and subjected to NFkB reporter assays. Data are presented as mean ± SD (n = 3). Two sided Student's t-test $p = 0.034$ (DMSO), $p = 0.582$ (TAK-931), respectively. Source data are provided as a Source Data file.

Supplementary Figure. 4

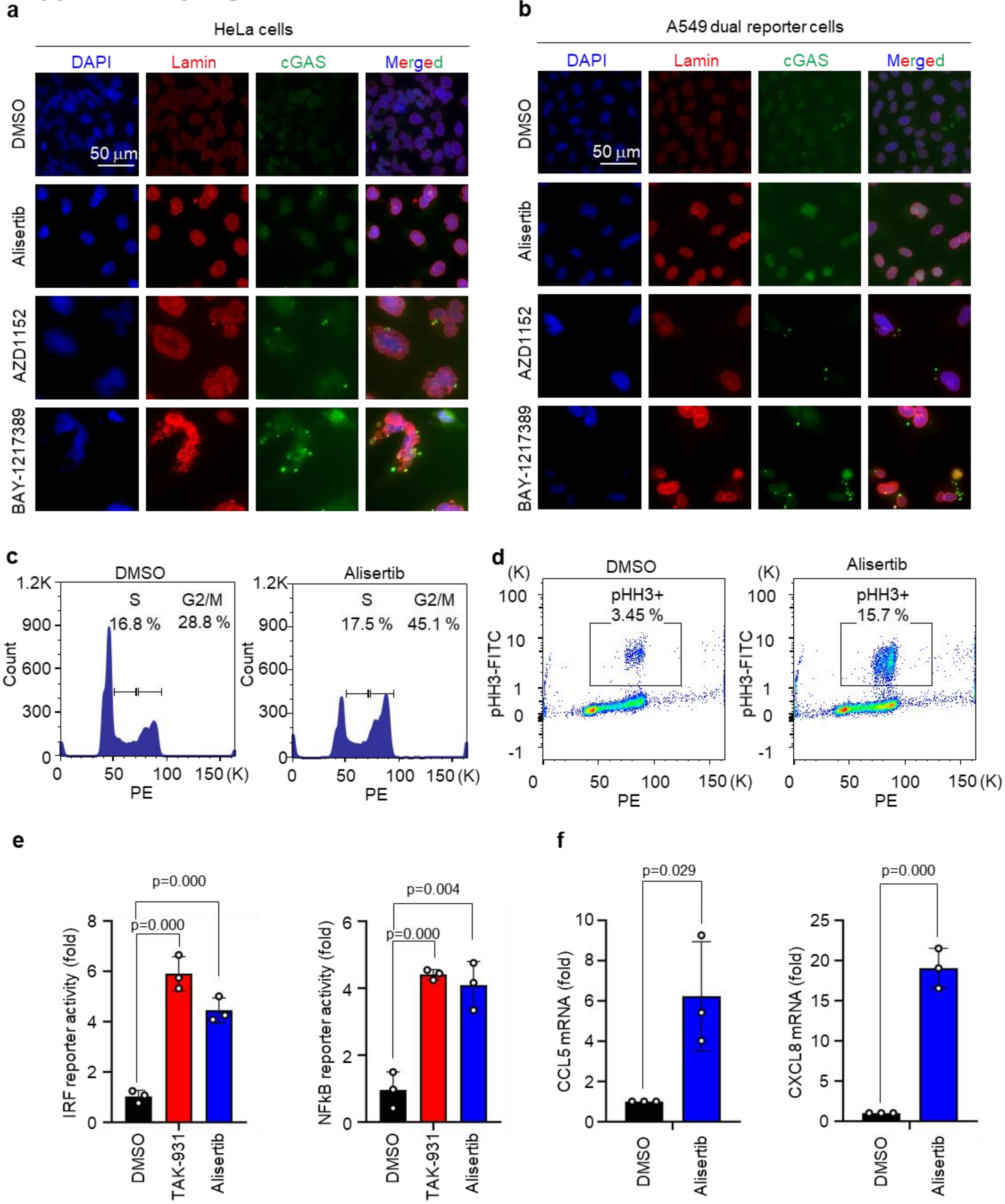

Supplementary Fig. 4. Alisertib-induced inflammatory activation in HeLa and A549-dual reproter cells

(a) (b) Effects of Alisertib, AZD-1152, and BAY-1217389 on micronucleus formation and cGAS nuclear localization. Green, red, and blue signals indicate cGAS, Lamin, and DAPI (DNA), respectively. HeLa cells (a) and A549 reporter cells (b) were treated with the indicated inhibitors (100 nM) for 72 h. White bars indicate 50 μm. (c) Representative histograms from cell cycle analysis of DMSO- (left) and Alisertib- (right) treated HeLa cells. HeLa cells were treated with DMSO or Alisertib at 100 nM for 24h. (d) Representative dot plots of pHH3 in DMSO- (left) and Alisertib- (right) treated HeLa cells. pHH3 was used as a mitotic index. (e) IRF and NFkB reporter activities in TAK-931- or Alisertib-treated cells. A549 cells were treated with TAK-931 (300 nM) or Alisertib (100 nM) at for 72 h and subjected to IRF-Luc and NFkB-SEAP reporter assays. Data are presented as mean ± SD (n = 3). Two sided Student's t-test $p = 0.000$ (DMSO vs TAK-931), $p = 0.000$ (DMSO vs Alisertib) (IRF reporter), $p = 0.000$ (DMSO vs TAK-931), $p = 0.004$ (DMSO vs Alisertib) (NFkB reporter), respectively. (f) qRT-PCR analysis of CCL5 (left) and CXCL8 (right) in DMSO- or Alisertib-treated HeLa cells Data are presented as mean ± SD (n = 3). Two sided Student's t-test $p = 0.029$, p = 0.000, respectively. Source data are provided as a Source Data file.

Supplementary Figure. 5

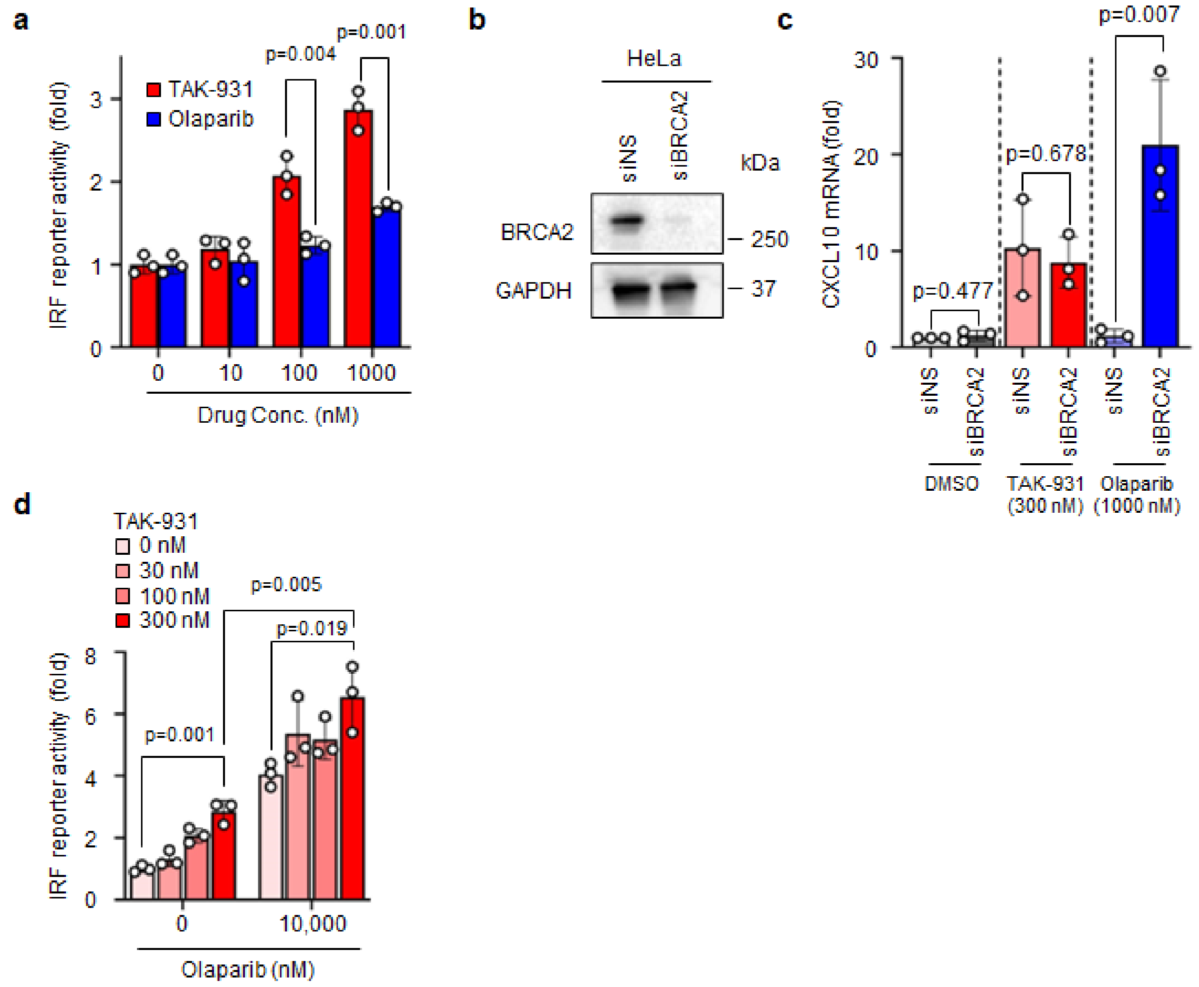

Supplementary Fig. 5. TAK-931- and olaparib induced inflammatory activation in BRCA2 knockdown cells

(a) IRF reporter activity in TAK-931 and olaparib treatments. A549 cells were treated with TAK-931 or olaparib at the indicated concentrations for 72 h and subjected to IRF-Luc reporter assays. Red and blue bars indicate TAK-931 and olaparib treatments, respectively. Data are presented as mean ± SD (n = 3). Two sided Student's t-test $p = 0.004$ (100 nM), $p = 0.001$ (1000 nM), respectively. (b) Immunoblotting of BRCA2 in HeLa cells transfected with siBRCA2 and siNS (negative siRNA). (c) qRT-PCR analysis of CXCL10 in siNS and siBRCA2 HeLa cells treated with DMSO, TAK-931 (300 nM) or olaparib (1000 nM). Data are presented as mean ± SD (n = 3). Two sided Student's t-test $p = 0.477$ (DMSO), $p = 0.678$ (TAK-931), p=0.007 (Olaparib), respectively. (d) Combination effects of TAK-931 and olaparib on IRF activity. A549 cells were treated with TAK-931 and olaparib at the indicated concentrations for 72 h and subjected to IRF-Luc reporter assays. Data are presented as mean ± SD (n = 3). Two sided Student's t-test $p = 0.001$ (TAK-931 0 nM vs TAK-931 300 nM), $p = 0.019$ (TAK-931 0 nM, Olaparib 10000 nM vs TAK-931 300 nM, Olaparib 10000 nM), p=0.005 (TAK-931 300 nM, Olaparib 0 nM vs TAK-931 300 nM, Olaparib 10000 nM), respectively. Source data are provided as a Source Data file.

Supplementary Figure. 6

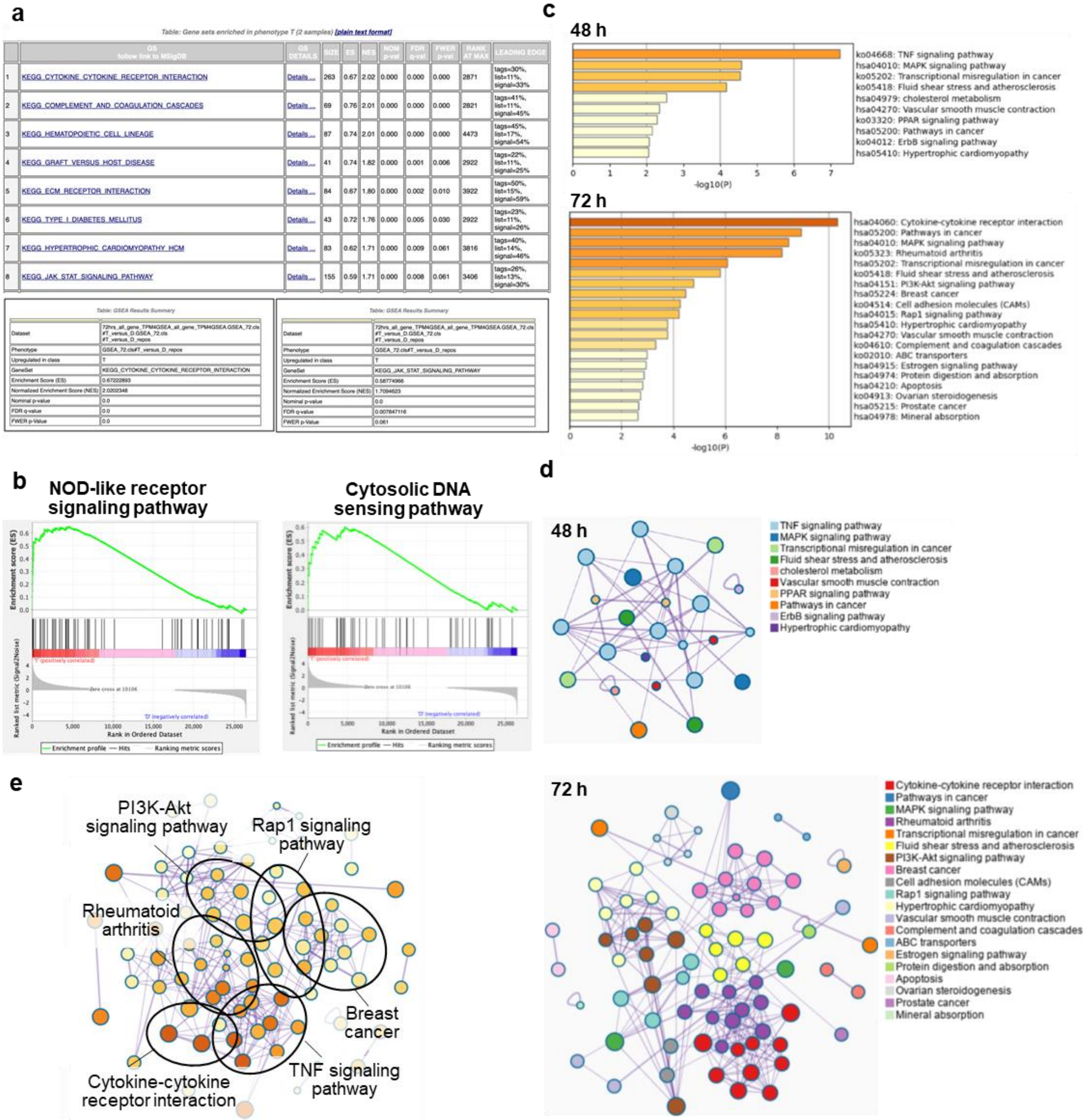

Supplementary Fig. 6. GSEAs in TAK-931-treated HeLa cells.

(a) Adjusted *p*-values of enriched KEGG hallmarks of GSEAs in TAK-931- or DMSO-treated HeLa cells (b) GSEAs of NOD-like receptor signaling pathway and cytosolic DNA sensing pathway are shown. RNA-seq data of HeLa cells in 72-h treatment with DMSO or TAK-931 were used. (c) The enrichment network colored by p-value of upregulating genes in 48-h (upper) and 72-h (lower) TAK-931 treatments. The KEGG IDs are also described. (d) Subextractor network analysis with the upregulated genes in TAK-931-treated HeLa cells. Upregulated genes in 48-h (upper) and 72-h (lower) TAK-931 treated HeLa cells were used. The network is visualized using Cytoscape (v3.1.2) by the 138 and 504 upregulated genes at 48 h (upper) and 72 h (lower) following TAK-931 treatment, respectively. Each term is represented by a circle node, where its size is proportional to the number of input genes fall into that term, and its color represent its cluster identity (i.e., nodes of the same color belong to the same cluster). (d) Cross-network analysis of upregulated genes between 48 h and 72 h TAK-931 treatments. The enrichment network is colored by p-value.

Supplementary Figure. 7

a

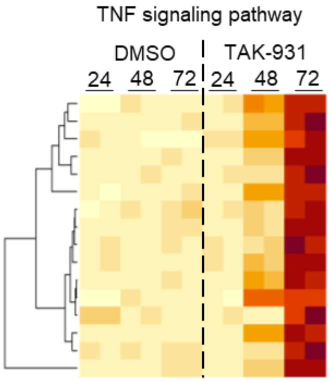

c

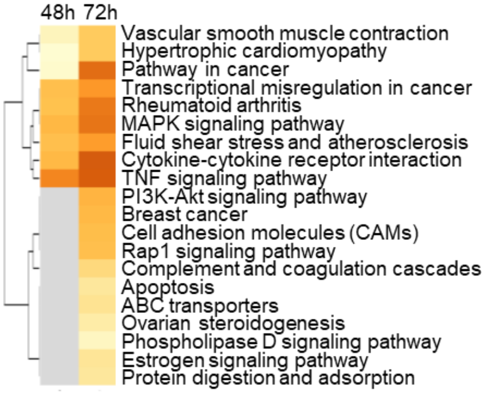

b

Cytokine-cytokine receptor interaction	TNF signaling pathways
JAG1	TNFAIP3
IL1R1	ICAM1
FOSL1	JAG1
PTGS2	PTGS2
CXCL1	CXCL1
ICAM1	LIF
CXCL8	CXCL3
NFKB2	CCL20
TNFAIP3	IL1B
LIF	BIRC3
OAS1	IL18R1
EDN1	TNFRSF1B
IL11	CCL5
CXCL2	EDN1
BIRC3	CXCL2
CXCL16	IL6
DDX58	
INHBA	
TNFSF15	
TNFRSF10C	
NGFR	
PDGFB	
FOSB	
MEFV	
OAS2	
SHC4	
ITGB2	
TNFRSF8	
CSF2RA	
MMP1	
CCL5	
ELMO1	
CARD11	
GDF9	
IL18R1	
TNFRSF1B	
IL24	
HSPA6	
IL7	
LCN2	
NGF	
IL1A	
IL1B	
CXCL3	
CCL20	
IL6	
ITPR1	
GDF15	

d

		TAK-931 (nM)				
		0	30	100	300	1000
Sapanisertib (nM)	0	100 ± 8	81 ± 4	49 ± 5	31 ± 3	25 ± 3
	10	94 ± 5	75 ± 4	42 ± 4	26 ± 2	27 ± 3
	30	88 ± 10	72 ± 5	39 ± 2	24 ± 1	24 ± 2
	100	61 ± 4	48 ± 5	25 ± 2	17 ± 1	18 ± 1
	300	45 ± 6	37 ± 3	21 ± 3	15 ± 1	17 ± 2
	1000	35 ± 2	27 ± 1	16 ± 1	11 ± 1	11 ± 1

Supplementary Fig. 7. Gene expression heatmaps in TAK-931-treated HeLa cells.

(a) Heatmaps of individual genes in the enriched TNF signaling pathway. The cells are colored according to their TPM. (b) List of genes in the heatmap of Cytokine-cytokine receptor interaction in Fig. 2g and TNF signaling pathway. (c) Heatmaps of the enriched pathways in TAK-931-treated HeLa cells. The heatmap cells are colored according to their TPM. White cells indicate the lack of enrichment for that term in the corresponding gene list. (d) *In vitr*o combination studies with TAK-931 and Sapanisertib in HeLa cells. HeLa cells were treated with TAK-931 and Sapanisertib at matrix dilution as described in Fig. 2h.

Supplementary Figure. 8

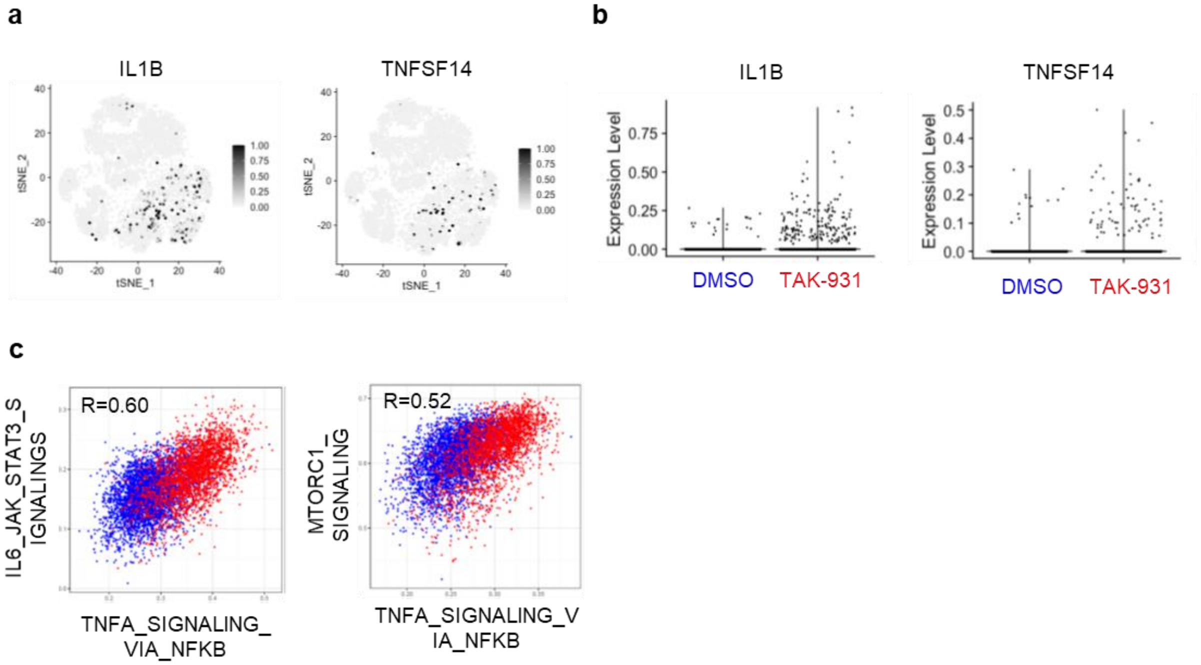

Supplementary Fig. 8. Single-cell transcriptional landscape in TAK-931-treated aneuploid cells.

(a) tSNE plots of inflammatory gene expression. Cells expressing *IL1B* (left) and *TNFSF14* (right) are colored in black (>1tag). (b) Dot plots of *IL1B* (left) and *TNFSF14*(right) expression levels in single cells treated with DMSO or TAK-931. (c) Comparison of enrichment scores in inflammatory-related hallmarks. The left and right panels indicate comparison of enrichment scores between TNFA signaling and IL6_JAK_STAT signaling, and TNFA signaling and mTORC1 signaling respectively. DMSO-treated and TAK-931-treated cells are shown in blue and red, respectively.

Supplementary Figure. 9

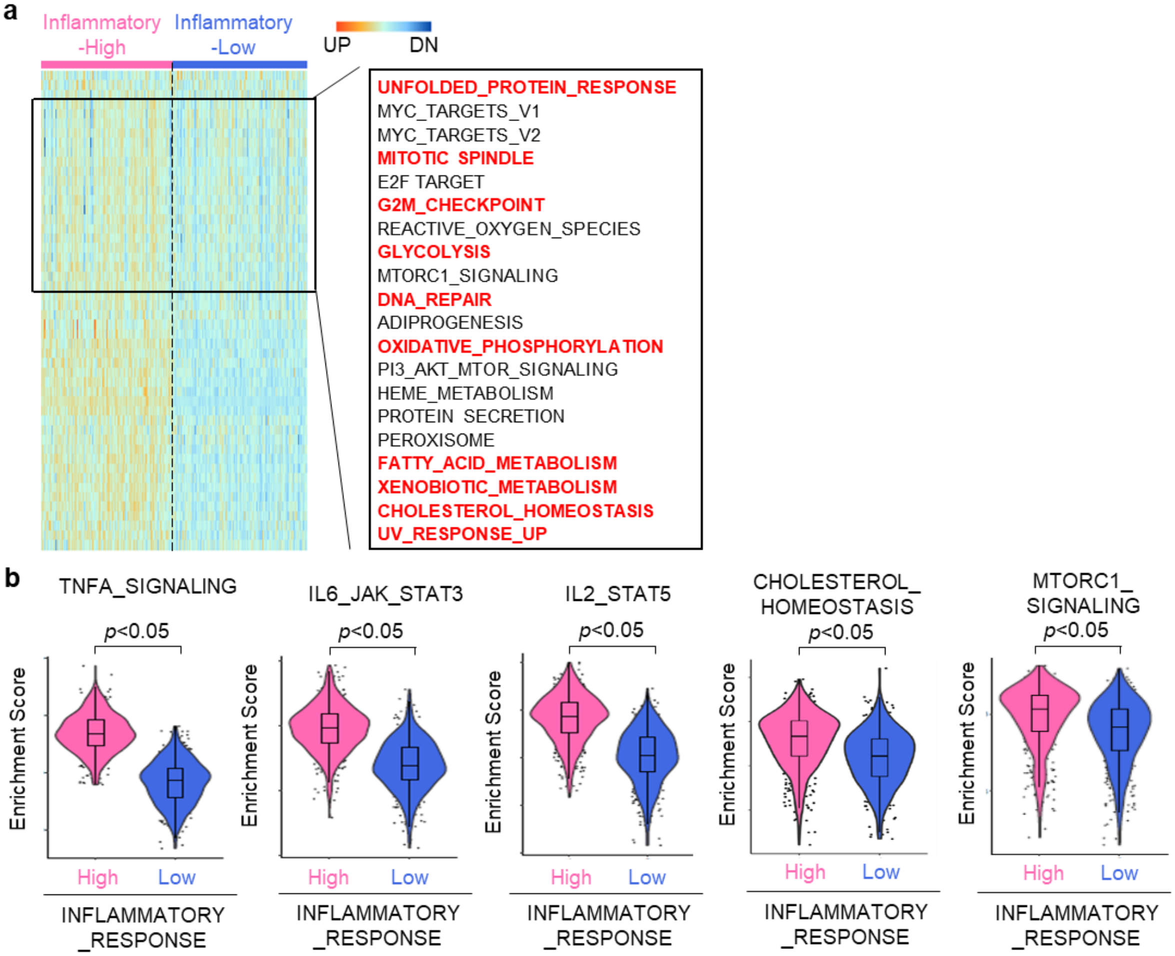

Suppelementary Fig. 9. Comparison analyses in scRNA-seq between inflammatory-high and low populations

(a) ssGSEA between inflammatory-high (pink) and low (sky blue) cells. Heatmap of the enrichment score of hallmark pathways is shown. The hallmarks related to aneuploid-associated stresses are highlighted in red. (b) Violin plot of enrichment score in inflammatory-related hallmarks. Violin plots of enrichment scores of TNFA, IL6_JAK_STAT3, IL2_STAT5, cholesterol homeostasis, and mTORC1 signaling are shown between inflammatory-high (pink) and inflammatory-low (sky blue) cell groups.

Supplementary Figure. 10

a

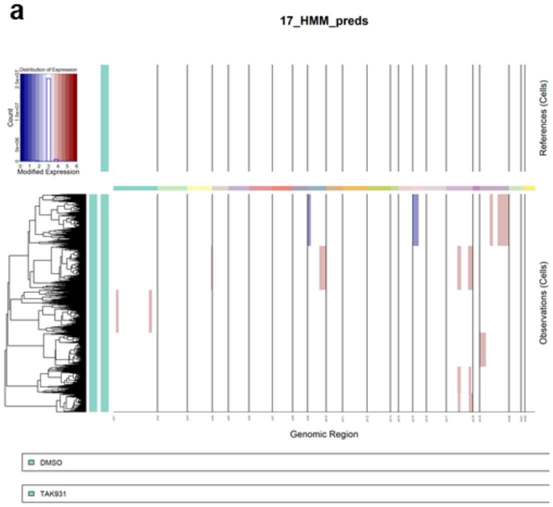

b

cell_group_name	cnv_name	state	chr	start	end
TAK931.TAK931_s1	chr9-region_9	2	chr9	121041	34637806
TAK931.TAK931_s1	chr15-region_16	2	chr15	22833395	59389618
TAK931.TAK931_s1	chr19-region_22	4	chr19	14800613	19754476
TAK931.TAK931_s1	chr19-region_24	4	chr19	40315990	59084942
TAK931.TAK931_s2	chr3-region_31	4	chr3	194308402	197770591
TAK931.TAK931_s2	chr9-region_38	4	chr9	125703112	140764468
TAK931.TAK931_s2	chr17-region_47	4	chr17	36956687	42767147
TAK931.TAK931_s2	chr17-region_49	4	chr17	73083822	81052864
TAK931.TAK931_s3	chr1-region_56	4	chr1	8064464	18024369
TAK931.TAK931_s3	chr1-region_58	4	chr1	185087220	209957904
TAK931.TAK931_s4	chr19-region_99	4	chr19	305575	9420514
TAK931.TAK931_s5	chr17-region_121	4	chr17	37026112	42277481
TAK931.TAK931_s5	chr17-region_123	4	chr17	73512141	79640934
TAK931.TAK931_s6	chr17-region_147	4	chr17	37884749	41910538
TAK931.TAK931_s6	chr17-region_149	4	chr17	73747675	81052864

c

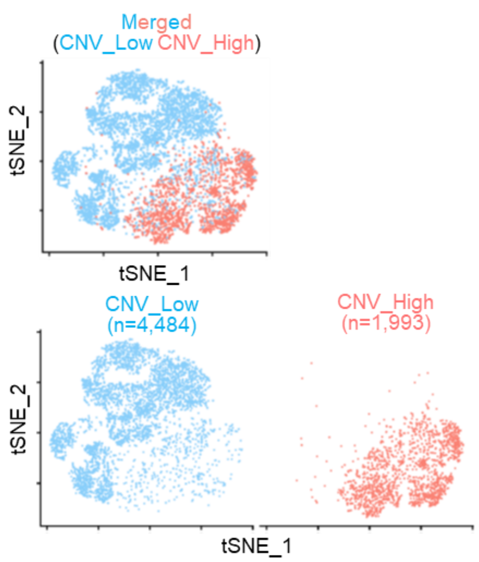

d

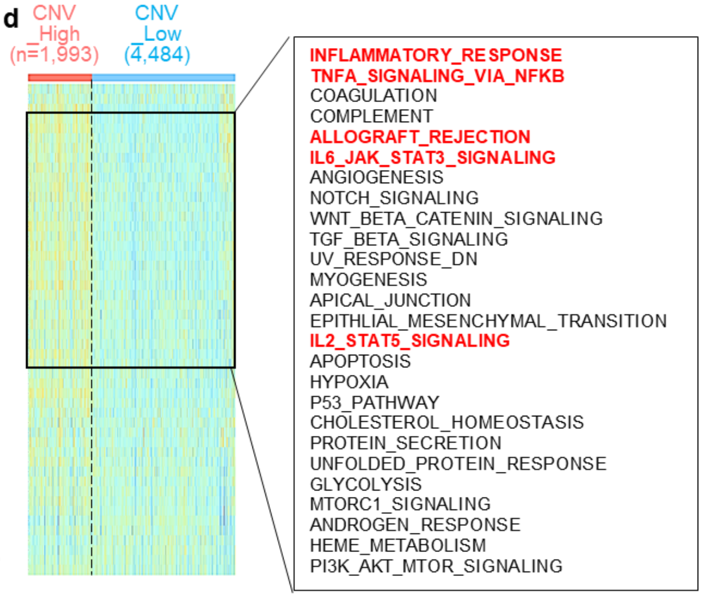

Supplementary Fig. 10. Comparison analyses in scRNA-seq between CNV-high and -low populations

(a) A six-state model for HMM-based CNV prediction (i6 HMM) in HeLa cells treated with DMSO or TAK-931. The heatmap of CNV regions in DMSO-treated (upper) and TAK-931-treated (lower) cells are shown. Red and blue indicate increased and decreased chromosomal copy numbers, respectively. (b) Summary table of specific CNV regions in TAK-931-treated HeLa cells. (c) tSNE plot based on the gene expression levels in copy number variation (CNV)-high (light orange) and -low (light blue) cells. (d) ssGSEA between CNV-high (light orange) and -low (light blue) cells. Heatmaps of the enrichment scores of hallmark pathways are shown. The inflammatory-related hallmarks are highlighted in red.

Supplementary Figure. 11

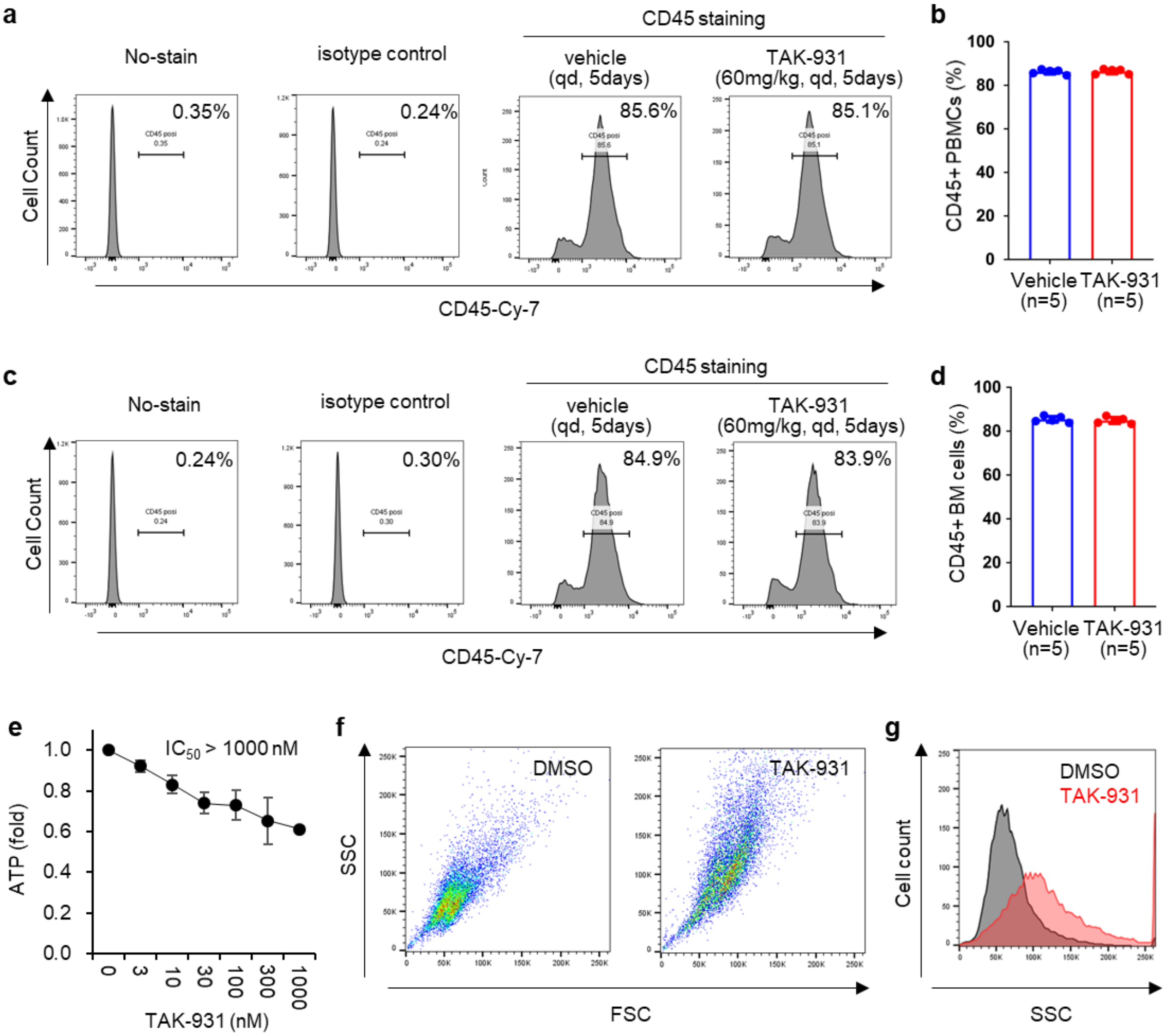

Supplementary Fig. 11. Effects of TAK-931 on hematopoietic cells in immune competent mouse model.

(a) Flow cytometry (FCM) for CD45 in peripheral blood mononuclear cells (PBMCs). PBMCs were collected from BALB/c mice orally administered with TAK-931 (60mg/kg, qd, 5days) or vehilce control. Single cell suspension of PBMCs was added Cy-7-conjugated anti-CD45 antibodies with 1×10^6 cells/tube. No-staining and isotype control were used as negarive controls. (b) Quantitative analyses of CD45+ PBMCs in vehicle (n=5, blue) and TAK-931 (n=5, red). Percentages of CD45+ PBMCs were shown. Statistical analysis was performed using parametric Student's *t*-test. (c) FCM for CD45 in bone marrow (BM) cells. BM cells were collected from BALB/c mice orally administered with TAK-931 (60mg/kg, qd, 5days) or vehilce control. Single cell suspension of BM cells was added Cy-7-conjugated anti-CD45 antibodies with 1×10^6 cells/tube. No-staining and isotype control were used as negarive controls. (d) Quantitative analyses of CD45+ BM cells in vehicle (n=5, blue) and TAK-931 (n=5, red). Percentages of CD45+ BM cells were shown. Statistical analysis was performed using parametric Student's *t*-test. (e) Growth assays of J558 cells treated with TAK-931 at the indicated concentrations for 72 h. Relative ATP amounts calculated with chemiluminescence assay and compared with the chemiluminescence value of 0 nM treatment are plotted (mean ± SD (n = 4)). (f) FSC and SSC histograms in TAK-931-treated J558 cells. J558 cells were treated with DMSO (left) or TAK-931 (right) for 72 h. (g) Side-scatter histograms of TAK-931-treated J558 cells. Source data are provided as a Source Data file.

Supplementary Figure. 12

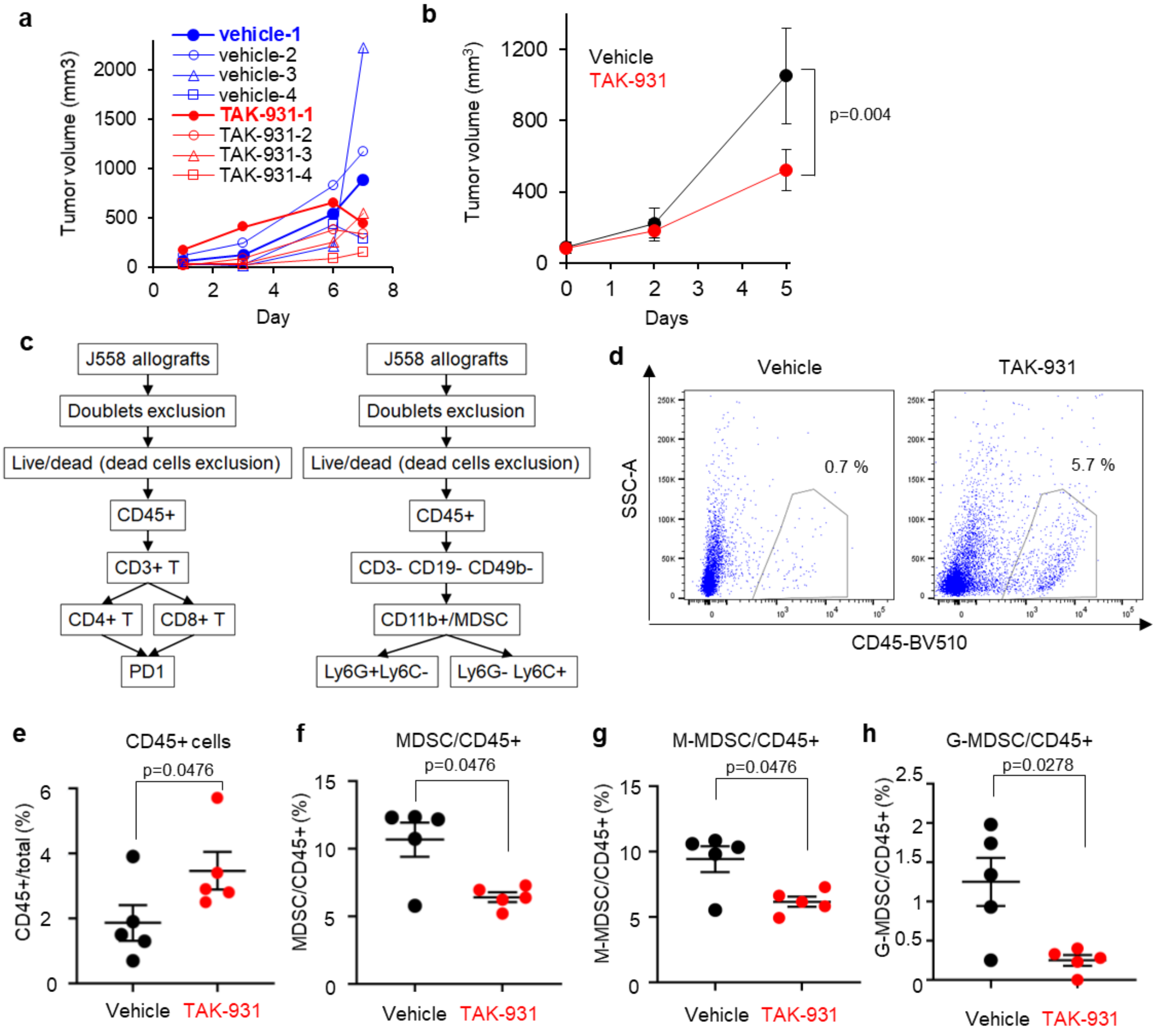

i

TIICs	Vehicle (% mean ± SD)	TAK-931 (% mean ± SD)	Fold change
CD45+/total	1.9 ± 1.2	3.5 ± 1.3	1.8*
CD3+/total	0.9 ± 0.6	1.9 ± 0.8	2.1*
PD1+/CD8+	12.2 ± 23.4	38.7 ± 24.2	3.2*
PD1+/CD4+	13.6 ± 2.8	37.2 ± 35.8	2.7*
MDSCs/CD45+	10.7 ± 2.8	6.4 ± 0.8	0.6*
M-MDSCs/CD45+	9.4 ± 2.2	6.2 ± 0.9	0.7*
G-MDSCs/CD45+	1.3 ± 0.7	0.3 ± 0.2	0.2*

Supplementary Fig. 12. FCM-based immune profiling panel studies in immune competent J558 syngeneic mouse models

(a) Efficacy results of 6-day TAK-931 treatment in J558 allograft model. Tumor volumes in individual mice are plotted for vehicle control (blue) and TAK-931 (red) treatments. Allografts form vehicle-1 and TAK-931-1 were used for IHC, bulk RNA-seq, scRNA-seq analyses as representative samples. (b) Efficacy results of 5-day TAK-931 treatment in J558 allograft model. Mean tumor volumes (mm^3 ± SD; n = 5) are plotted for vehicle control (black) and TAK-931 (red) treatments. Two sided Student's t-test p = 0.004. (c) Flow cytometry (FCM) immune panel design in J558 allograft model. Every obtained single cell suspension sample was added antibodies respectively with 1×10^6 cells/tube in 96-v-bottom plate according to the indicated FCM panel design. If the total cells number was not enough, divided them equally for ten tubes in which cells were no less than 5×10^5 cells. (d) Representative FACS staining of $CD45^+$ hematopoietic cells in vehicle-treated (left) and TAK-931-treated (right) J558 allografts collected at 24 h after the last (5th) dose. (e)-(h) Quantitative analyses of $CD45^+$ hematopoietic cells, $CD45^+$ MDSC cells, $CD45^+$ M-MDSCcells, and G-MDSC cells in J558 allografts. Percentages of $CD45^+$ hematopoietic cells (e), $CD45^+$ MDSCs (f), $CD45^+$ M-MDSCs (g), and $CD45^+$ G-MDSCs (h) are shown. Vehicle and TAK-931 treatments are described in black and red, respectively. Statistical analysis was performed using one sided non-parametric Wilcoxon-Mann-Whitney test p=0.0476, p=0.0476, p=0.0476, p=0.0278, respectively. (i) Summary of FCM-based immune profiling panel studies in J558 allograft TIICs. * indicates statistically significant differences at $p < 0.05$. Source data are provided as a Source Data file.

Supplementary Figure. 13

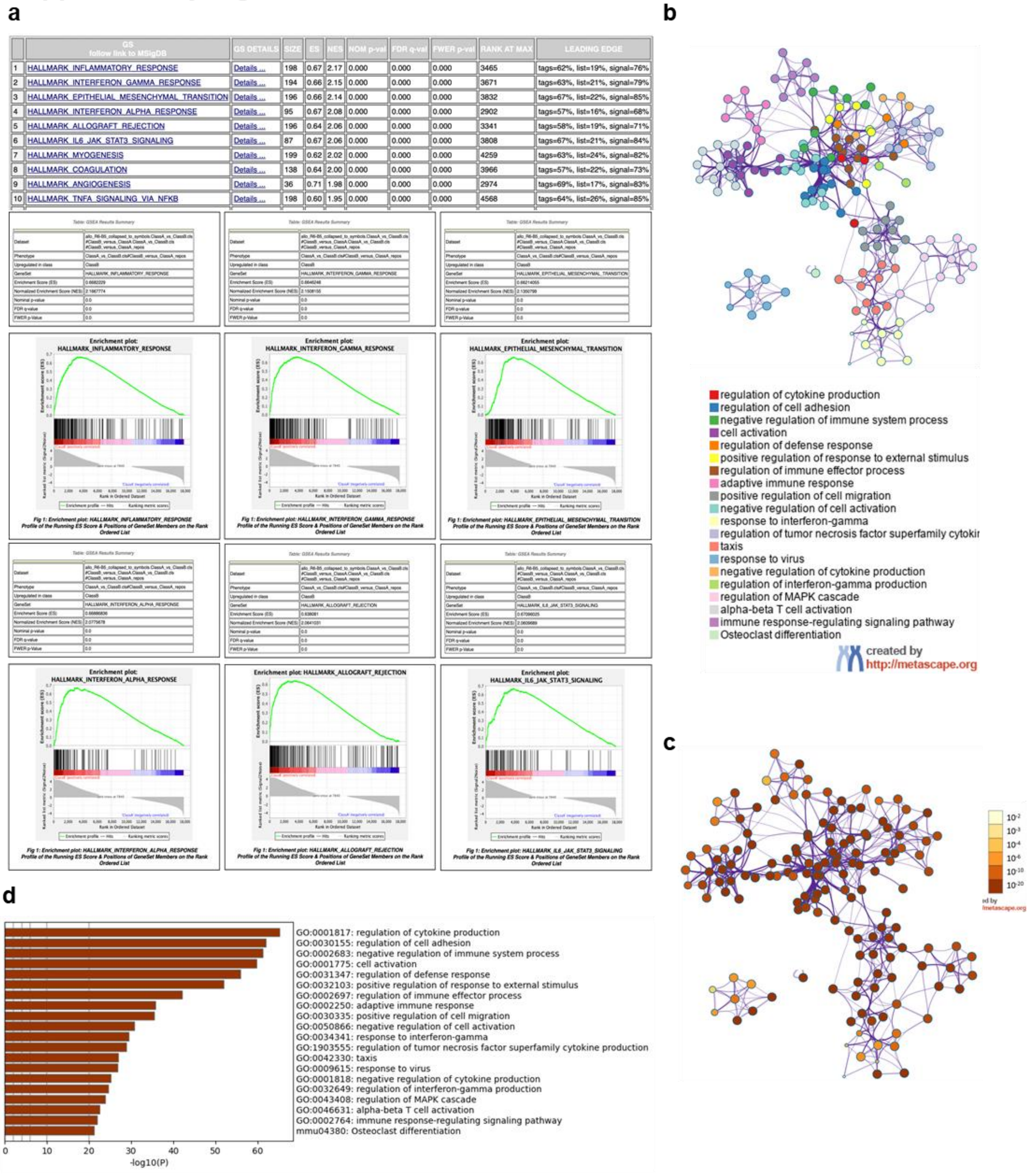

	GS follow link to MSigDB	GS DETAILS	SIZE	ES	NES	NOM p-val	FDR q-val	FWER p-val	RANK AT MAX	LEADING EDGE
1	HALLMARK_INFLAMMATORY_RESPONSE	Details ...	198	0.67	2.17	0.000	0.000	0.000	3465	tags=62%, list=19%, signal=76%
2	HALLMARK_INTERFERON_GAMMA_RESPONSE	Details ...	194	0.66	2.15	0.000	0.000	0.000	3671	tags=63%, list=21%, signal=79%
3	HALLMARK_EPITHELIAL_MESENCHYMAL_TRANSITION	Details ...	196	0.66	2.14	0.000	0.000	0.000	3832	tags=67%, list=22%, signal=85%
4	HALLMARK_INTERFERON_ALPHA_RESPONSE	Details ...	95	0.67	2.08	0.000	0.000	0.000	2902	tags=57%, list=16%, signal=68%
5	HALLMARK_ALLOGRAFT_REJECTION	Details ...	196	0.64	2.06	0.000	0.000	0.000	3341	tags=58%, list=19%, signal=71%
6	HALLMARK_IL6_JAK_STAT3_SIGNALING	Details ...	87	0.67	2.06	0.000	0.000	0.000	3808	tags=67%, list=21%, signal=84%
7	HALLMARK_MYOGENESIS	Details ...	199	0.62	2.02	0.000	0.000	0.000	4259	tags=63%, list=24%, signal=82%
8	HALLMARK_COAGULATION	Details ...	138	0.64	2.00	0.000	0.000	0.000	3966	tags=57%, list=22%, signal=73%
9	HALLMARK_ANGIOGENESIS	Details ...	36	0.71	1.98	0.000	0.000	0.000	2974	tags=69%, list=17%, signal=83%
10	HALLMARK_TNFA_SIGNALING_VIA_NFKB	Details ...	198	0.60	1.95	0.000	0.000	0.000	4568	tags=64%, list=26%, signal=85%

Supplementary Fig. 13. Transcriptome analyses of bulk RNA-seq in J558 syngeneic mouse model

(a) GSEAs of representative inflammation-related terms in J558 allografts. The RNA-seq data of J558 allografts after 6-days treatment with vehicle (vehicle-1) and TAK-931 (TAK-931-1) were used. (b)-(c) Subextractor network analysis of the upregulated genes in TAK-931-treated allografts. The network was visualized using Cytoscape (v3.1.2). Each term is represented by a circle node, where its size is proportional to the number of input genes fall into that term, and its color represent its cluster identity (i.e., nodes of the same color belong to the same cluster) (b). The enrichment network is colored by p-value (c). (d) The enrichment network colored by *p*-value of upregulating genes in TAK-931-treated allografts. The GO term IDs are also described.

Supplementary Figure. 14

a

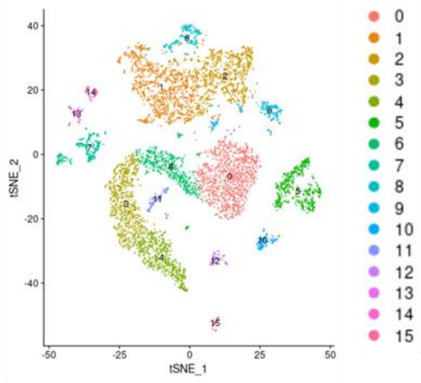

b

Cls	marker	Annotation
0	Trap1a	Plasmacytoma
1	CD14	Monocyte
2	CD14	Monocyte
3	Trap1a	Plasmacytoma
4	Trap1a	Plasmacytoma
5	CD3D, CD8A	CD8+ T cell
6	Trap1a	Plasmacytoma
7	Pecam1	Endothelial cell
8	CD14	Monocyte
9	CD14	Monocyte
10	CD3D, CD4	CD4+ T cell
11	Trap1a	Plasmacytoma
12	PTPRC, NKG7	NK cell
13	Col1a	Fibroblast
14	Col1a	Fibroblast
15	PTPRC	Unidentified

c

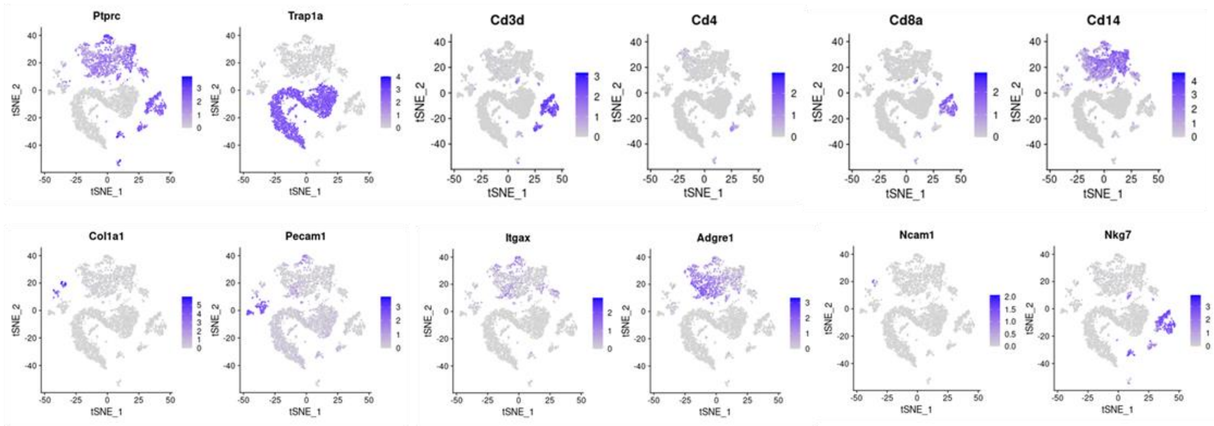

d

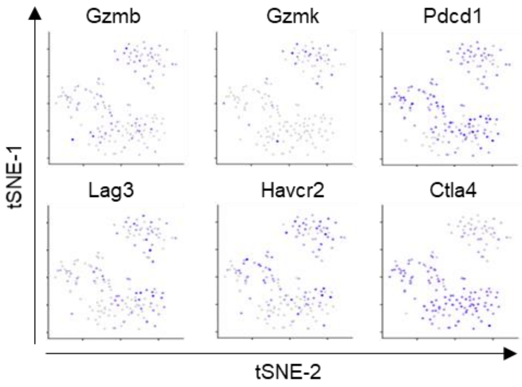

Supplementary Fig. 14. Transcriptome analyses of scRNA-seq in J558 syngeneic mouse model

(a) tSNE plot based on the gene expression of the allografts after 6-days treatment with vehicle (vehicle-1) and TAK-931 (TAK-931-1). Clusters were classified into the cellular components by the marker gens described in Fig. S14b. (b) Summary table of the markers for the cellular components in TME of J558 allograft. (c) Gene expression plots of the representative marker genes. (d) Gene expression tSNE plots in $CD4^+$ T cells. $CD4^+$ T cells expressing the indicated marker genes are colored in purple.

Supplementary Figure. 15

a

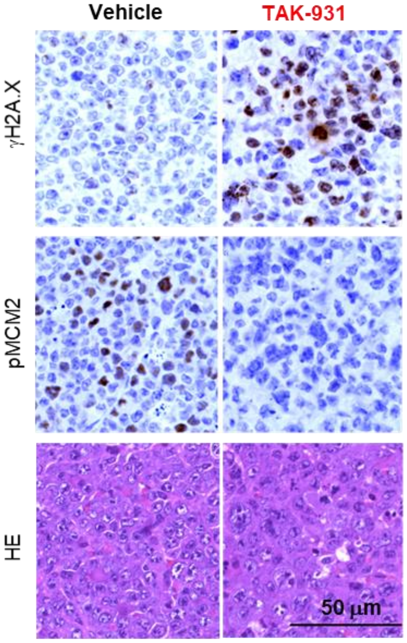

b

	J558 allograft (nude mouse model)
Log-rank *p*-value	*p*< 0.05 (0.02)
Median survival (Days)	Vehicle: 7<br>TAK-931 (60 mg/kg, QD): 9
Complete Regression (CR)	Vehicle: 0/6<br>TAK-931 (60 mg/kg, QD): 0/7

c

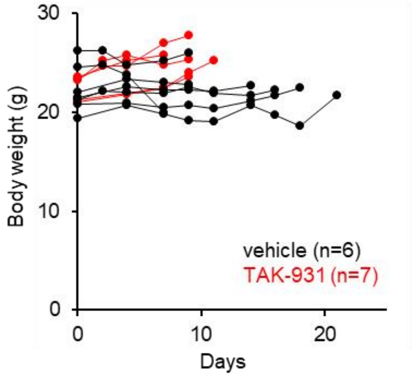

d

Group	Treatment Drugs	TAK-931		n	Mean Tumor Volume ± SEM	GRI (%)	GRI *p*-value	CR	Death	Nadir BWL(%)
		Dose (mg/kg)	Regimen							
1	Vehicle	0	-	10	2702.2 ± 224.8	-	-	0	0	0
2	TAK-931	60	QD x 21	10	622.2 ± 163.2	48	*p*<0.01	4	0	0.6

e

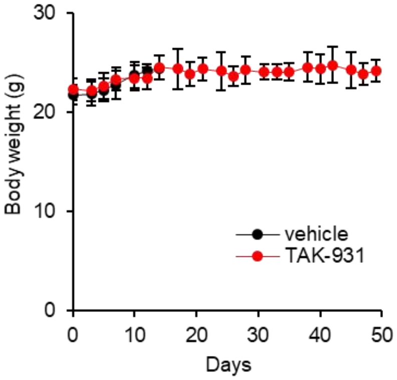

f

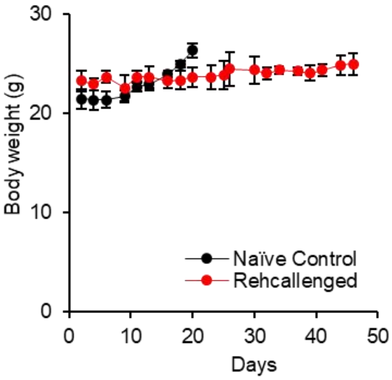

g

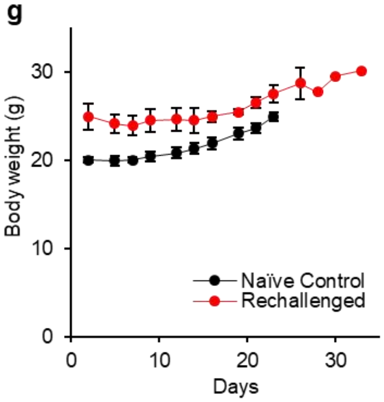

Supplementary Fig. 15. Antitumor efficacy studies using single-agent TAK-931 and tumor rechallenge studies

(a) Immunohistochemistry of γH2A/X and pMCM2 in the tumor sections from J558 allograft nude mice after efficacy studies, vehicle (left) for 4 days or TAK-931 (right) for 11 days. γH2A/X was used as RS and DNA damage marker. pMCM2 was used as target-engagement PD biomarker. Black bars indicate 50 μm. (b) Summary of single-agent treatment of TAK-931 in the J558 allograft nude mice model. (c) Bodyweight changes of TAK-931 orally administered at the indicated regimen in the J558 allograft nude mouse model. Black (n=6) and red (n=7) indicate vehicle and TAK-931 treatments, respectively. The data are plotted as mean body weight (g) ± SD. (d) Summary of antitumor efficacy studies using single-agent TAK-931 in the J558 allograft immune competent mouse model. (e)-(g) Bodyweight changes of TAK-931 orally administered at the indicated regimen in the J558 allograft mouse model. Black and red indicate vehicle and TAK-931 treatments, respectively. The data are plotted as mean body weight (g) ± SD; n = 10. First (f) and second (g) tumor-rechallenge studies in J558 and CT26 mouse syngeneic models, respectively. Black and red indicate naïve mice (n = 5) and rechallenged CRs (n = 4), respectively. The data are plotted as mean body weight (g) ± SD. Source data are provided as a Source Data file.

Supplementary Figure. 16

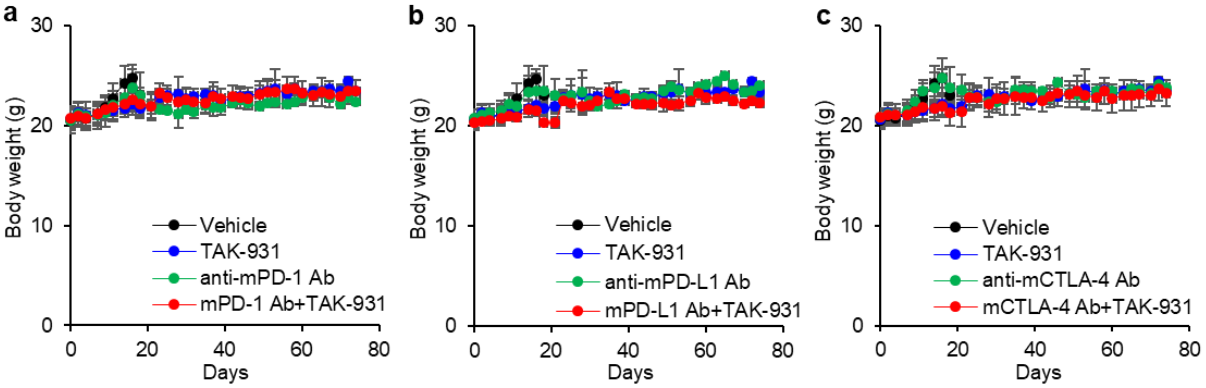

Supplementary Fig. 16. Body weight changes of the combination treatment with TAK-931 and ICIs in the J558 mouse allograft models

(a)–(c) Body weight data of J558 allograft model administered combination TAK-931 and ICI treatment. The J558-allograft mice were administered m-PD-1 (a), m-PD-L1 (b), or m-CTLA-4 (c) in combination with TAK-931 at the indicated regimens. Black, blue, green, and red indicate vehicle, TAK-931, ICIs, and the combination treatments, respectively. The data are plotted as mean body weight (g) ± SD; n = 10. Source data are provided as a Source Data file.

Supplementary Table. 1

		CCL5	CXCL1	CXCL8	CXCL10	IL1A	IL1B	IL6	TNFRSF1B	TNFSF15
**BRCA**	HR (High)	0.67	0.65	0.92	0.92	0.79	0.97	0.93	0.77	1.1
	Log rank (p)	**0.014**	**0.0094**	0.6	0.61	0.17	0.84	0.64	0.12	0.44
**COAD**	HR (High)	0.77	0.69	0.61	0.86	0.58	0.61	1.2	0.98	1
	Log rank (p)	0.29	0.13	0.05	0.56	**0.031**	**0.043**	0.45	0.94	0.88
**ESCA**	HR (High)	0.93	0.83	1.3	1.5	1.1	1.2	1.4	1	1.1
	Log rank (p)	0.76	0.43	0.3	0.071	0.67	0.45	0.12	0.85	0.68
**LUAD**	HR (High)	0.78	1	1.2	1.1	1.1	0.89	0.99	0.81	0.85
	Log rank (p)	0.1	1	0.28	0.64	0.71	0.43	0.92	0.16	0.28
**LUSC**	HR (High)	1	0.97	1.2	1.1	1.2	1.3	1.2	1.2	1.2
	Log rank (p)	0.91	0.79	0.3	0.67	0.14	0.055	0.17	0.29	0.17
**OV**	HR (High)	0.81	0.95	1	0.72	0.99	1.1	1.1	1	0.95
	Log rank (p)	0.081	0.68	0.98	**0.0079**	0.93	0.36	0.53	0.84	0.7
**PAAD**	HR (High)	1.1	0.91	1.1	1.8	1.3	1.1	1.5	0.99	0.99
	Log rank (p)	0.57	0.67	0.59	**0.0044**	0.21	0.8	**0.048**	0.97	0.98
**PRAD**	HR (High)	1.1	0.75	1.1	1.4	1.2	0.45	0.63	0.79	1.1
	Log rank (p)	0.84	0.66	0.9	0.57	0.8	0.24	0.47	0.71	0.86
**READ**	HR (High)	0.82	0.66	1	0.6	0.59	0.77	1.2	1.1	0.58
	Log rank (p)	0.68	0.41	0.96	0.29	0.3	0.59	0.76	0.8	0.26
**SKCM**	HR (High)	0.53	1.2	1	0.57	0.97	0.73	0.85	0.64	0.77
	Log rank (p)	**3.20E-06**	0.11	0.84	**2.80E-05**	0.83	**0.02**	0.25	**0.00099**	0.06

HR (High)

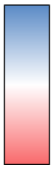

Supplementary Table. 1. Summary table of hazard ratios (HR) based on inflammatory-related gene expression in various tumor types

HRs for upregulated expression of the indicated genes at < 1 and ≥ 1 are highlighted in red and blue, respectively. Signifiance diference ($p < 0.05$) is higligted in bold . Source data are provided as a Source Data file.
