# Supplementary material for: CDC7 inhibition induces replication stress-mediated aneuploid cells with an inflammatory phenotype sensitizing tumors to immune checkpoint blockade
Source: Nat Commun. 2023 Nov 18;14:7490. doi: 10.1038/s41467-023-43274-3 (PMC10657413; doi:10.1038/s41467-023-43274-3)
Supplement: Supplementary file 2 — Reporting Summary [file 41467_2023_43274_MOESM2_ESM.pdf]

nature portfolio

Corresponding author(s): Dr Akihiro Ohashi

Last updated by author(s): Oct 9, 2023

Reporting Summary

Nature Portfolio wishes to improve the reproducibility of the work that we publish. This form provides structure for consistency and transparency in reporting. For further information on Nature Portfolio policies, see our Editorial Policies and the Editorial Policy Checklist.

Statistics

For all statistical analyses, confirm that the following items are present in the figure legend, table legend, main text, or Methods section.

n/a	Confirmed	
☐	☒	The exact sample size (*n*) for each experimental group/condition, given as a discrete number and unit of measurement
☐	☒	A statement on whether measurements were taken from distinct samples or whether the same sample was measured repeatedly
☐	☒	The statistical test(s) used AND whether they are one- or two-sided<br>*Only common tests should be described solely by name; describe more complex techniques in the Methods section.*
☐	☒	A description of all covariates tested
☐	☒	A description of any assumptions or corrections, such as tests of normality and adjustment for multiple comparisons
☐	☒	A full description of the statistical parameters including central tendency (e.g. means) or other basic estimates (e.g. regression coefficient) AND variation (e.g. standard deviation) or associated estimates of uncertainty (e.g. confidence intervals)
☐	☒	For null hypothesis testing, the test statistic (e.g. *F*, *t*, *r*) with confidence intervals, effect sizes, degrees of freedom and *P* value noted<br>*Give P values as exact values whenever suitable.*
☒	☐	For Bayesian analysis, information on the choice of priors and Markov chain Monte Carlo settings
☒	☐	For hierarchical and complex designs, identification of the appropriate level for tests and full reporting of outcomes
☒	☐	Estimates of effect sizes (e.g. Cohen's *d*, Pearson's *r*), indicating how they were calculated

Our web collection on statistics for biologists contains articles on many of the points above.

Software and code

Policy information about availability of computer code

Data collection

Bulk RNA sequencing (RNA-seq) and scRNA-seq were performed by NovaSeq 6000 (Illumina Inc., San Diego, CA, USA).

Data analysis

Network analysis for bulk RNA sequencing
Bioinformatics analysis for upregulated genes identified in the transcriptome analysis was performed as described previously (ref. 15). The upregulated genes were submitted to Metascape (metascape.org.) to identify the statistically enriched terms. The terms with the best p-values within each cluster were selected as representative terms and displayed in a dendrogram. The heatmap cells were colored according to their p-values: white cells indicate the lack of enrichment for that term. A subset of representative terms from the complete cluster was selected to convert them into a network layout. The network was visualized using Cytoscape (v3.1.2) with “force-directed” layout and edge bundled for clarity. The same enrichment network had its nodes colored according to p-value: the darker the color, the more statistically significant the node is (see legend for p-value ranges). The nodes for the same enrichment network were displayed as pies. Each pie sector is proportional to the number of hits originated from a gene list.

scRNA-seq experiment procedure
scRNA-seq was performed as described previously (ref. 57). Chromium Single Cell 3′ Solution (v3.1 Chemistry; 10×Genomics, Pleasanton, CA, USA) was used per the manufacturer’s recommendations.The cells were resuspended at 1×106 cells per mL. To generate gel bead-in emulsions (GEMs), the master mix was mixed with the cell suspension, and gel beads and partitioning oils were loaded on a Chromium chip. Next, GEM-reverse transcription (RT) reaction, cDNA amplification, and gene expression library generation were performed using Chromium kits and reagents. QC of constructed libraries were conducted with Agilent Bioanalyzer (Agilent technologies).

scRNA-seq data processing with Seurat

After library generation, sequencing was performed using a NovaSeq 6000 (Illumina Inc.). The fastq files were generated from the bcl files in Cell Ranger (version 6.0, 10×Genomics). The sequence reads were aligned to UCSC hg38, and Unique Molecular Identifiers were counted for each gene in each cell barcode using Cell Ranger count. The data were then processed by R package Seurat using Cell Ranger output files, and barcodes.tsv, genes.tsv, and matrix.mtx (Seurat version 4.0)(DOI:https://doi.org/10.1016/j.cell.2021.04.048). In HeLa cell study, cells were filtered based on unique feature counts (nFeature_RNA > 5000) and mitochondrial counts (percent.mt < 10) for quality control. In mouse model scRNA-seq analysis, cells were filtered based on unique feature counts (nFeature_RNA > 2000) and mitochondrial counts (percent.mt < 15) for quality control.

GSEA analysis using scRNA-seq dataset
To calculate gene set enrichment score, ssGSEA analysis were performed using R packages (R package "escape", version 1.0.058) (R package "dittoSeq", version 1.2.559). Gene set c "Human MSigDB Collections H:hallmark" was selected for analysis.

scRNA-seq-based copy number variation analyses
R package InferCNV (https://github.com/broadinstitute/inferCNV) was used to predict somatic large-scale CNV (gains or deletions of entire chromosomes or large segments of chromosomes) for scRNA-seq data in HeLa cells treated with DMSO or TAK-931(R version 4.1.0 "Camp Pontanezen", InferCNV version 1.9.1)33,34. Analyses were performed according to the instruction shown in inferCNV wiki "Using 10x data". First, raw count matrix of single-cell RNA-seq gene expression was extracted from Seurat object. In addition to the raw counts matrix, annotation file and gene/chromosome position files are prepared as input. Using those inputs, object was created using command "CreateInfercnvObject"(parameters were default). CNV scores were calculated using "infercnv::run" (cutoff score was 0.1, other parameters were default). Scores are returned to Seurat object and following analysis were conducted. A six-state model for HMM-based CNV prediction, i6 HMM, (https://github.com/broadinstitute/infercnv/wiki/inferCNV-HMM-based-CNV-Prediction-Methods) was used to predicts the CNV levels at the chromosome regions.

For manuscripts utilizing custom algorithms or software that are central to the research but not yet described in published literature, software must be made available to editors and reviewers. We strongly encourage code deposition in a community repository (e.g. GitHub). See the Nature Portfolio guidelines for submitting code & software for further information.

Data

Policy information about availability of data

All manuscripts must include a data availability statement. This statement should provide the following information, where applicable:

- Accession codes, unique identifiers, or web links for publicly available datasets
- A description of any restrictions on data availability
- For clinical datasets or third party data, please ensure that the statement adheres to our policy

The RNA-seq dataset of TAK-931 treated HeLa cells are available in the GEO database under accession code ZZ [Add hyperlink here]. The single cell RNA-seq dataset of TAK-931 treated HeLa cells are available in the GEO database under accession code ZZ [Add hyperlink here]. The RNA-seq dataset of J558 allograft mouse model treated with TAK-931 are available in the GEO database under accession code ZZ [Add hyperlink here]. The Single cell NRA-seq dataset of of J558 allograft mouse model treated with TAK-931 are available in the GEO database under accession code ZZ [Add hyperlink here]. The remaining data are available within the Article, Supplementary Information or Source Data file.

Research involving human participants, their data, or biological material

Policy information about studies with human participants or human data. See also policy information about sex, gender (identity/presentation), and sexual orientation and race, ethnicity and racism.

Reporting on sex and gender	No study with human participants or human data is included in the manuscript.
Reporting on race, ethnicity, or other socially relevant groupings	No study with human participants or human data is included in the manuscript.
Population characteristics	No study with human participants or human data is included in the manuscript.
Recruitment	No study with human participants or human data is included in the manuscript.
Ethics oversight	No study with human participants or human data is included in the manuscript.

Note that full information on the approval of the study protocol must also be provided in the manuscript.

Field-specific reporting

Please select the one below that is the best fit for your research. If you are not sure, read the appropriate sections before making your selection.

[x] Life sciences [ ] Behavioural & social sciences [ ] Ecological, evolutionary & environmental sciences

For a reference copy of the document with all sections, see nature.com/documents/nr-reporting-summary-flat.pdf

Life sciences study design

All studies must disclose on these points even when the disclosure is negative.

Sample size	Sample sizes in the preclinical studies in vitro and in vivo were determined based on previous experience (ref. 14, 15 and 56), providing enough statistic robustness and reproducibility.
Data exclusions	No data was excluded from the analyses.
Replication	We performed multiple independent in vitro experiments on a minimum of n=3 groups to ensure reproducibility. In vivo experiments were performed with n=4 or more sample groups for each of the two independent group.
Randomization	In all experiments, grouping was performed randomly.
Blinding	This study did not involve any clinical trials requiring blinding.

Reporting for specific materials, systems and methods

We require information from authors about some types of materials, experimental systems and methods used in many studies. Here, indicate whether each material, system or method listed is relevant to your study. If you are not sure if a list item applies to your research, read the appropriate section before selecting a response.

Materials & experimental systems

n/a	Involved in the study	
☐	☒	Antibodies
☐	☒	Eukaryotic cell lines
☒	☐	Palaeontology and archaeology
☐	☒	Animals and other organisms
☒	☐	Clinical data
☒	☐	Dual use research of concern
☒	☐	Plants

Methods

n/a	Involved in the study	
☒	☐	ChIP-seq
☐	☒	Flow cytometry
☒	☐	MRI-based neuroimaging

Antibodies

Antibodies used

The following antibodies were used in this study.
[Immunoblotting]
anti-pMCM2 (EPR4170(2)), 1:1000 (ab133243; Abcam)
anti-MCM2 (E-8), 1:1000 (sc-373702; Santa Cruz Biotechnology)
anti-pTBK1 (D52C2), 1:1000 (# 5483; Cell Signaling Technology)
anti-TBK1 (D1B4), 1:1000 (# 3504; Cell Signaling Technology)
anti-pSTING (D8K6H), 1:1000 (# 40818; Cell Signaling Technology)
anti-STING (D2P2F), 1:1000 (# 13647; Cell Signaling Technology)
anti-pIRF3 (4D4G), 1:1000 (#4947; Cell Signaling Technology)
anti-IRF3 (D83B9), 1:1000 (# 4302; Cell Signaling Technology)
anti-BRCA2 (D9S6V), 1:1000 (#10741; Cell Signaling Technology)
anti-GAPDH (14C10), 1:1000 (# 2118; Cell Signaling Technology).
[Immunohistochemistry]
anti-CD3 (SP7) (GeneTex, #GTX16669)
anti-CD4 (D7D2Z) (#25229, Cell Signaling Technology)
anti-CD8 (D4W2Z) (#98941, Cell Signaling Technology)
anti-CD11c (N418) (GeneTex, #GTX74940)
anti-pMCM2 (EPR4170(2)), 1:900 (Abcam, #ab133243)
anti-PD-1 (D7D5W) (#84651, Cell Signaling Technology)
anti-gH2AX (Ser139) (20E3), 1:600 (#9718, Cell Signaling Technology)
[FACS analysis]
Phospho-Histone H3 (Ser 10) (D2C8) (Alexa Fluor® 488 Conjugate) antibody, 1:50 (#3465; Cell Signaling Technology)
[Immunofluorescence assay]
anti-α tubulin (DM1A), 1:100 (T9026; Sigma-Aldrich)
anti-cGAS (D1D3G), 1:100 (# 15102; Cell Signaling Technology)
anti-Lamin B (C-20), 1:100 (sc-6216; Santa Cruz Biotechnology)

Validation

All antibodies were validated and purchased from commercial vendorsand were used according to the manufscturer's instructions.

anti-pMCM2 (ab133243; Abcam)
https://www.abcam.com/products/primary-antibodies/mcm2-phospho-s40-antibody-epr41702-bsa-and-azide-free-ab240052.html
anti-MCM2 (sc588 373702; Santa Cruz Biotechnology)
https://www.scbt.com/ja/p/mcm2-antibody-e-8
anti-pTBK1 (# 5483; Cell Signaling Technology)
https://www.cellsignal.jp/products/primary-antibodies/phospho-tbk1-nak-ser172-d52c2-xp-rabbit-mab/5483
anti-TBK1 589 (# 3504; Cell Signaling Technology)
https://www.cellsignal.jp/products/primary-antibodies/tbk1-nak-d1b4-rabbit-mab/3504
anti-pSTING (# 40818; Cell Signaling Technology)
https://www.cellsignal.jp/products/primary-antibodies/phospho-sting-ser366-d8k6h-rabbit-mab/40818
anti-STING (# 13647; Cell Signaling Technology)
https://www.cellsignal.jp/products/primary-antibodies/sting-d2p2f-rabbit-mab/13647
anti-pIRF3 (#4947; Cell Signaling Technology)
https://www.cellsignal.jp/products/primary-antibodies/phospho-irf-3-ser396-4d4g-rabbit-mab/4947
anti-IRF3 (# 4302; Cell Signaling Technology)
https://www.cellsignal.jp/products/primary-antibodies/irf-3-d83b9-rabbit-mab/4302
anti-BRCA2 (#10741; Cell Signaling Technology)
https://www.cellsignal.jp/products/primary-antibodies/brca2-d9s6v-rabbit-mab/10741
anti-GAPDH (# 2118; Cell Signaling Technology)
https://www.cellsignal.jp/products/primary-antibodies/gapdh-14c10-rabbit-mab/2118
anti-CD3 (GeneTex, #GTX16669)
https://www.genetex.com/Product/Detail/CD3-antibody-SP7/GTX16669
anti-CD4 (#25229, Cell Signaling Technology)
https://www.cellsignal.jp/products/primary-antibodies/cd4-d7d2z-rabbit-mab/25229
anti-CD8 (#98941, Cell Signaling Technology)
https://www.cellsignal.jp/products/primary-antibodies/cd8a-d4w2z-xp-rabbit-mab/98941
anti-CD11c (GeneTex, #GTX74940)
https://www.genetex.com/Product/Detail/CD11c-antibody-N418/GTX74940
anti-PD-1 (#84651, Cell Signaling Technology)
https://www.cellsignal.jp/products/primary-antibodies/pd-1-intracellular-domain-d7d5w-xp-rabbit-mab/84651
anti-Phospho-Histone H3 (Ser 10) (Alexa Fluor® 488 Conjugate)(#3465; Cell Signaling Technology)
https://www.cellsignal.jp/products/antibody-conjugates/phospho-histone-h3-ser10-d2c8-xp-rabbit-mab-alexa-fluor-488-conjugate/3465

Eukaryotic cell lines

Policy information about cell lines and Sex and Gender in Research

Cell line source(s)	HeLa and A549 cells were purchased from RIKEN BRC. COLO205, J558 and CT26 cells were purchased from ATCC. PBMC was purchased from COSMO BIO CO., LTD. A549-Dual Cells were purchased from InvivoGen.
Authentication	Cells were authenticated using STR profiling by the vendor or depositor and no further authentication was performed in the laboratory.
Mycoplasma contamination	Cells were regularly screened and confirmed negative for mycoplasma contamination. .. All cell lines were negative for mycoplasma infection.
Commonly misidentified lines (See ICLAC register)	No commonly misidentified cell lines were used.

Animals and other research organisms

Policy information about studies involving animals; ARRIVE guidelines recommended for reporting animal research, and Sex and Gender in Research

Laboratory animals	BALB/cAJcl and BALB/cAJcl-nu/nu, 4 weeks of age, female. Mice were housed in cages under specific pathogen free conditions, provided with standard food, given free access to hypochlorous weak-acid water, and maintained in a 12 h light/ 12 h dark cyclic environment with the temperature 24±1°C and humidity at 50±10%.
Wild animals	No wild animal was used in this study.
Reporting on sex	No sex-based analysis was performed
Field-collected samples	No field collected samples were used in the study.
Ethics oversight	The protocol and any amendments or procedures involving the care and use of animals in this study were reviewed and approved by the Institutional Animal Care and Use Committee of Medicilon Inc., Takeda Pharmaceutical Company ltd., or National Cancer Center Japan before study initiation (K20-09, K-21-015, study protocol#17054, #17081, and#17098).

Note that full information on the approval of the study protocol must also be provided in the manuscript.

Flow Cytometry

Plots

Confirm that:

- [x] The axis labels state the marker and fluorochrome used (e.g. CD4-FITC).
- [x] The axis scales are clearly visible. Include numbers along axes only for bottom left plot of group (a 'group' is an analysis of identical markers).
- [x] All plots are contour plots with outliers or pseudocolor plots.
- [x] A numerical value for number of cells or percentage (with statistics) is provided.

Methodology

Sample preparation	Flow cytometry experiments were performed on cell lines and tumor samples. For in vitro experiments, cells were collected, fixed with 70% ethanol, washed in PBS containing 4% fetal bovine serum and stained with antibodies according to standard procedures. For in vivo experiments, J558 allografted tumor samples were sliced into small pieces and then isolated into single cells using gentle MACS Dissociator (Miltenyi Biotec, Bergisch Gladbach, Germany) at 37 °C for 40 min using themouse tumor dissociation kit (Miltenyi Biotech, 130-096-730). The digested tumors were filtered through a 70-µm strainer to remove cell aggregates. The cells were resuspended with 30% Percoll and were carefully layered on 70% Percoll. The cell suspension was centrifuged at 400 × g for 30 min at room temperature, and then the cell layer between 30% and 70% Percoll was harvested. After being washed with PBS, the cells were resuspended in BD staining buffer, and the cell numbers were counted on a counting plate under a microscope. The isolated cells were aliquoted into a 96-well V-bottom plate, and after the indicated antibody mixture was added, the samples were incubated for 30–60 min at 4 °C in the dark. After fixation with BD fixation buffer at 4 °C for 30 min in the dark, the samples were resuspended in 200–400 µL of staining buffer and incubated at 4 °C for 0–24 h in the dark for flow cytometry analysis.
Instrument	Flow cytometry analysis was performed on either a BD FACSCanto II (BD biosciences), a FACSCelesta (BD biosciences).
Software	Data were collected using BD FACSDiva software (BD Biosciences). Data were analyzed using FlowJo 10.7.1
Cell population abundance	Purity for viable cells was >90%
Gating strategy	For all experiments, all cells were gated by FSC area vs. SSC area, and singlets were gated by FSC area vs. FSC height.

- [x] Tick this box to confirm that a figure exemplifying the gating strategy is provided in the Supplementary Information.
